# The Impact of Patterns in Linkage Disequilibrium and Sequencing Quality on the Imprint of Balancing Selection

**DOI:** 10.1093/gbe/evae009

**Published:** 2024-02-01

**Authors:** Tristan J Hayeck, Yang Li, Timothy L Mosbruger, Jonathan P Bradfield, Adam G Gleason, George Damianos, Grace Tzun-Wen Shaw, Jamie L Duke, Laura K Conlin, Tychele N Turner, Marcelo A Fernández-Viña, Mahdi Sarmady, Dimitri S Monos

**Affiliations:** Division of Genomic Diagnostics, Department of Pathology and Laboratory Medicine, Children's Hospital of Philadelphia, Philadelphia, PA, USA; Department of Pathology and Laboratory Medicine, Perelman School of Medicine, University of Pennsylvania, Philadelphia, PA, USA; Division of Genomic Diagnostics, Department of Pathology and Laboratory Medicine, Children's Hospital of Philadelphia, Philadelphia, PA, USA; Department of Pathology and Laboratory Medicine, Perelman School of Medicine, University of Pennsylvania, Philadelphia, PA, USA; Division of Genomic Diagnostics, Department of Pathology and Laboratory Medicine, Children's Hospital of Philadelphia, Philadelphia, PA, USA; Quantinuum Research LLC, Philadelphia, PA, USA; Division of Genomic Diagnostics, Department of Pathology and Laboratory Medicine, Children's Hospital of Philadelphia, Philadelphia, PA, USA; Division of Genomic Diagnostics, Department of Pathology and Laboratory Medicine, Children's Hospital of Philadelphia, Philadelphia, PA, USA; Division of Genomic Diagnostics, Department of Pathology and Laboratory Medicine, Children's Hospital of Philadelphia, Philadelphia, PA, USA; Division of Genomic Diagnostics, Department of Pathology and Laboratory Medicine, Children's Hospital of Philadelphia, Philadelphia, PA, USA; Division of Genomic Diagnostics, Department of Pathology and Laboratory Medicine, Children's Hospital of Philadelphia, Philadelphia, PA, USA; Department of Pathology and Laboratory Medicine, Perelman School of Medicine, University of Pennsylvania, Philadelphia, PA, USA; Department of Genetics, Washington University School of Medicine, St. Louis, MO 63110, USA; Department of Pathology, Stanford University School of Medicine, Palo Alto, CA, USA; Histocompatibility and Immunogenetics Laboratory, Stanford Blood Center, Palo Alto, CA, USA; Division of Genomic Diagnostics, Department of Pathology and Laboratory Medicine, Children's Hospital of Philadelphia, Philadelphia, PA, USA; Department of Pathology and Laboratory Medicine, Perelman School of Medicine, University of Pennsylvania, Philadelphia, PA, USA; Division of Genomic Diagnostics, Department of Pathology and Laboratory Medicine, Children's Hospital of Philadelphia, Philadelphia, PA, USA; Department of Pathology and Laboratory Medicine, Perelman School of Medicine, University of Pennsylvania, Philadelphia, PA, USA

**Keywords:** balancing selection, statistical genetics, Bayesian, population genetics, sequencing platform, linkage disequilibriumhuman, human leukocyte antigen genes

## Abstract

Regions under balancing selection are characterized by dense polymorphisms and multiple persistent haplotypes, along with other sequence complexities. Successful identification of these patterns depends on both the statistical approach and the quality of sequencing. To address this challenge, at first, a new statistical method called LD-ABF was developed, employing efficient Bayesian techniques to effectively test for balancing selection. LD-ABF demonstrated the most robust detection of selection in a variety of simulation scenarios, compared against a range of existing tests/tools (Tajima's *D*, HKA, *D*_ng_, BetaScan, and BalLerMix). Furthermore, the impact of the quality of sequencing on detection of balancing selection was explored, as well, using: (i) SNP genotyping and exome data, (ii) targeted high-resolution HLA genotyping (IHIW), and (iii) whole-genome long-read sequencing data (Pangenome). In the analysis of SNP genotyping and exome data, we identified known targets and 38 new selection signatures in genes not previously linked to balancing selection. To further investigate the impact of sequencing quality on detection of balancing selection, a detailed investigation of the MHC was performed with high-resolution HLA typing data. Higher quality sequencing revealed the HLA-DQ genes consistently demonstrated strong selection signatures otherwise not observed from the sparser SNP array and exome data. The HLA-DQ selection signature was also replicated in the Pangenome samples using considerably less samples but, with high-quality long-read sequence data. The improved statistical method, coupled with higher quality sequencing, leads to more consistent identification of selection and enhanced localization of variants under selection, particularly in complex regions.

**Significance**

Understanding evolutionary selection is critical to disentangling the connections between genetic variation and response to environmental exposures. Both analytical approach and quality of sequencing impact the ability to detect balancing selection. Our new statistical model, LD-ABF, leverages phased data to improve detection of balancing selection signatures by looking for patterns of linkage disequilibrium and density of polymorphisms on haplotypes. A total of 38 new selection signatures were identified in genes that were not previously known as being associated with balancing selection. Of the 38 new selection signals, fourteen were exclusively detected by LD-ABF whereas the remaining 24 were replicated by two or more methods. Notably, in the context of the canonical example of the HLA genes, we were able to better isolate the strong selection signal in HLA-DQ genes.

This, DQ-related signal, is not always observed in SNP array and exome sequencing but, is replicated consistently across world populations with targeted genotyping (IHIW) and in long-read samples (Pangenome). Further, we demonstrate that with improved sequencing, it is possible to detect the same evolutionary selection with considerably smaller sample sizes.

## Introduction

**Fig. 1.**
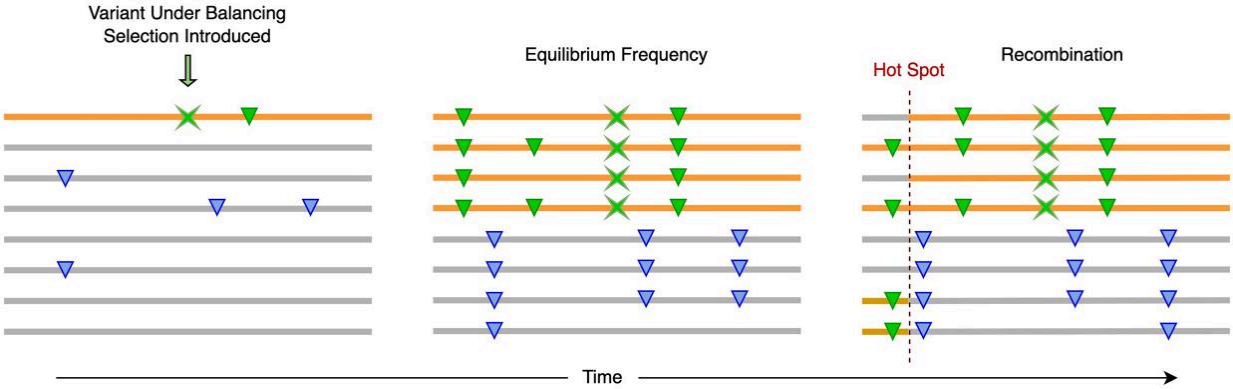
Evolutionary diagram depicting the progression of an allele under balancing selection. The X denotes the variant under selection, triangles are variants originating on the same haplotype denoted by the top lines with balancing selection variant. In the first pane, the variant is introduced on a single haplotype. Then after some time has passed evolutionary pressures favoring multiple alleles at the position of focus maintaining both haplotypes with and without the polymorphism, where hitchhiking effects are observed around the variant under balancing selection–inducing LD patterns. Recombination breaks the strong LD resulting in mosaics of the haplotypes, where strong hotspots will diffuse the LD effects of hitchhiking.

Improved detection and understanding of balancing selection in the human genome can provide valuable insight into heritable diseases and our species' adaptation to varying environmental exposures (Sabeti et al. 2007; Andrés et al. 2009; Davydov et al. 2010; Gussow et al. 2016; Bitarello et al. 2018; Johnson and Voight 2018; Palamara et al. 2018; Hayeck et al. 2019, 2022). Balancing selection takes place when evolutionary pressures maintain multiple alleles across a population. This stands in contrast to the process of negative selection (Davydov et al. 2010; Gussow et al. 2016; Hayeck et al. 2019, 2022), which alone eliminates alleles harmful to fitness whereas positive selection drives favorable alleles toward fixation (Sabeti et al. 2007; Johnson and Voight 2018; Palamara et al. 2018). Negative selection or a full selective sweep toward fixation may result in overall depletion in variation over genomic regions under selective pressure whereas balancing selection results in more variation. When balancing selection occurs, it not only affects the frequency of the variant directly under evolutionary pressure, but surrounding variants on the same haplotypes will also rise in frequency, in a process known as hitchhiking (Fig. 1). As a result, neutral regions near a locus associated with balanced polymorphism will undergo an extended coalescence period. This will unveil denser clusters of closely positioned variants and impacts the local linkage disequilibrium (LD) patterns. Linkage disequilibrium refers to the correlation among variants, and over time, recombination diminishes the original LD, concentrating it more locally around the variants under selection (Slatkin 2008). Making inference on haplotypic patterns can improve power to detect selection (DeGiorgio et al. 2014; Tennessen and Duraisingh 2021). The most accurate detection of LD requires phased data to make inference on the probability of observing multiple alleles on the same haplotype.

However, phased data are not always available. Another approach to detect balancing selection is to test for deviations in the site frequency spectrum to find regions with elevated allele frequencies or deviation from expectation under neutral evolution (Tajima 1989; Wright and Charlesworth 2004). These tests for detecting deviation from expected neutral drift may be under powered in identifying selective signals though. While there are tests specifically designed to directly assess LD, they are primarily tailored toward detecting positive selection and often focus on extended haplotypes that may have gone to fixation (Voight et al. 2006; Sabeti et al. 2007; Ferrer-Admetlla et al. 2014; Johnson and Voight 2018; Palamara et al. 2018). Consequently, these tests are less well-suited to detect balancing selection.

To test for transspecies cases of balancing selection, alternative approaches utilize data from two closely related species to look for relative differences in sequence context (Hudson et al. 1987; DeGiorgio et al. 2014; Siewert and Voight 2017, 2020; Bitarello et al. 2018; Cheng and Degiorgio 2019). However, relying on polymorphism analysis across closely related species primarily captures ancient signals that potentially affect the fitness of multiple species. So, these approaches may have limited sensitivity in detecting signatures or more recent selective pressures. While the identification of transspecies polymorphisms and the development of methods to detect such selective signals provide strong evidence of balancing selection, they likely represent only a small fraction of the overall balanced polymorphisms (Asthana et al. 2005; Tennessen and Duraisingh 2021). Methods that train their models in part with simulated and real data (Sheehan and Song 2016; Isildak et al. 2021) have the potential for high power, however they require considerably more resources like large grids of simulation or difficult to acquire training data along with specific model and evolutionary assumptions which may result in overfitting.

We concentrated on creating a test statistic that utilizes phased data to make direct inference on LD and investigate the role sequencing quality plays on such methods ability to detect selection. We developed LD approximate Bayesian factor (LD-ABF), a new robust statistical method that directly investigates balancing selection by testing for both, density of polymorphisms and strength of LD on haplotypes. LD-ABF builds on the population genetics models of Siewert and Voight, which tested for patterns of balancing selection by looking for an excess of proximal SNPs that have very similar allele frequencies to the core SNP. Advancing from their approach LD-ABF requires phased haplotypes to make direct inference on LD which improves power of detection of more subtle or recent selection signals.

Since almost all methods (including LD-ABF) will be impacted by the density of polymorphisms, patterns of balancing selection were investigated using three distinct datasets derived from varying sequencing technologies to understand how different types of sequencing data impact the ability to detect selection signatures. First, we conducted a genome-wide selection scan using phased high-quality SNP array and exome sequence data derived from 468 clinical samples, including 334 probands from trios (Table 1). Utilizing clinical trios provided improved phasing accuracy as the haplotypes of the children could be directly inferred from their parents (related samples removed after phasing), enhancing both the phasing and downstream statistical inference. Second, we focused in on the major histocompatibility complex (MHC). This region is of significant interest since it is critical to our immune response and is known to be under strong evolutionary pressure. However, the complex genomic nature of the MHC poses challenges for effective sequencing, often resulting in its neglect. To address this, we used targeted genotyping data focused on the HLA genes from thousands of unrelated haplotypes worldwide in the 17th International HLA and Immunogenetics Workshop (IHIW) (Creary et al. 2021). Lastly, we validated our findings and identified complex signal artifacts using an independent set of high-quality long-read whole-genome sequencing (WGS) samples from the Human Pangenome Reference Consortium (Liao et al. 2023). This additional validation step helped ensure the reliability and accuracy of our results.

**Table 1:** Detailed counts for CHOP trios and individuals collected for analysis that include both SNP array data and whole exome sequence data

Population	Individuals	Duo	Trio	Totals
AFR	11	9	34	54
AMR	8	12	44	64
EAS	10	1	17	28
EUR	47	33	221	301
SAS	1	2	18	21
Totals	77	57	334	468

The analysis of these samples involved performing genome-wide scans for balancing selection within the population, employing the different test statistics: LD-ABF, *D*_ng_, Tajima's *D*, and B2. Related individuals were removed to avoid biasing the analysis and the proband counts are listed here.

## Results

### Overview of LD-ABF

Approaches to assess balancing selection by quantifying local polymorphisms and LD patterns are complicated by both rare variants (resulting in sparse data) and instances of close or perfect LD among variants (resulting in quasi or fully separated data). To address this, we implemented a Bayesian logistic regression model using logF priors (the conjugate family for binomial logistic regression) which have been shown to be effective in settings of both sparse and fully separated data without making major assumptions (Greenland and Mansournia 2015; Rahman and Sultana 2017). The model with logF priors can be fit using established data augmentation techniques to efficiently estimate posterior coefficients (Greenland 2003, 2007; Greenland and Mansournia 2015). Then to test how well a SNP predicts its neighboring variants, we derived an ABF (Raftery 1995; Kass and Raftery 1995), where nested models are fit with and without a logistic regression coefficient for the test SNP being associated with its neighboring variant. Finally, the log of the products of ABFs for every base in a set window (here 1 kilobase [kb] was used) is taken to derive a combined score that measures both the density of polymorphisms and degree of LD around the test SNP (Methods). It is important to note this Bayesian approach scales with the sample size, meaning across populations with different sample sizes the test statistics will be on different scales.

### Balancing Selection Simulations

Forward time simulations were implemented in SLiM 3.0 (Haller and Messer 2019a, 2019b) and different statistics' ability to detect variants under selection, versus neutral drift, was compared. Primary focus was on the first two sets replicating scenarios as described in previous studies as benchmarks (Siewert and Voight 2017, 2020) to demonstrate relative utility of the new method. The last scenarios investigate more recent balancing selection. The simulation framework, adapted from Siewert and Voight, is designed to approximate three specific timescales: (i) the point of divergence between humans and chimpanzees (equivalent to around 250,000 generations, labeled “older”); (ii) the period when the Homo clade underwent diversification (approximately 100,000 generations, labeled “younger”); and (iii) the emergence of *Homo sapiens* (about 10,000 generations ago, labeled “recent”). For events categorized as “older,” the selection mutation arises and progresses through 250,000 generations in the simulation. Conversely, for “younger” events, the balancing selection mutation is introduced, and the simulation advances through 100,000 generations. The supplementary section also delves into an additional scenario involving more “recent” balancing selection, occurring 10,000 generations in the past.

For each simulation scenario, sample sizes of 10,000 were generated across 10 kb windows, assuming mutation rate and recombination rates of 2.5 × 10^−8^. In all cases, the balancing selection variant is introduced at the center of the 10 kb region in simulation. An ancestral population is simulated for 100,000 generations and then a split occurs (to compare against B2 and *β*_2,std_, which requires closely related species) then three different balancing selection scenarios are simulated. In all time settings, three different equilibrium frequencies scenarios were simulated, {0.25, 0.5, 0.75}, assuming heterozygous fitness of 1+hs using a selection coefficient *s* of 10^−2^ and over dominance coefficient *h* dependent on the desired equilibrium allele frequency corresponding to {−0.5, 100, 1.5} (Hartl and Clark 2007). An equilibrium frequency of 0.75 indicates the derived allele is under enough positive selection that it becomes more common than the ancestral allele. These simulations therefore also indicate some level of detection of positive selection as well, assuming the evolutionary pressure is not a selective sweep that is strong enough to induce full fixation of the allele. Another set of additional simulations were run with “younger” mutations and an equilibrium frequency of 50% with a dominance coefficient *h* at 100 but instead a selection coefficient of 10^−4^. This gave another scenario keeping the relative *s* ∗ *h* ratio closer across simulations, relative to the equilibrium frequencies of 25% and 75%. Further, for this set test statistics were computed for window sizes of (i) 100, (ii) 500, (iii) 1,000, and (iv) 5,000 bp to look for impact of window size on test statistics. For each of the ten total scenarios (three-time points vs. three equilibrium frequencies and one looking at window sizes), two thousand simulations were run along with an additional neutral set where no balancing selection variant was introduced after the split.

The LD-ABF is compared to the HKA statistic (Hudson et al. 1987), Tajima's *D* (Tajima 1989), BetaScan *β*_2,std_, (Siewert and Voight 2017, 2020), B2 (Cheng and DeGiorgio 2020), and *D*_ng_ statistic (Tennessen and Duraisingh 2021). Both HKA and Tajima's *D* are classic population genetics tests, where HKA detects signatures of excess polymorphism and Tajima's *D* tests shifts in the site frequency spectrum. Beta Scan's *β* looks at a test statistic and compares the weighted regional mutation rate relative to a neutral estimate. Beta Scan's *β*_2,std_ and BalLerMix's B2 makes inference across species leveraging ancestral similarities and differences of closely related species (both *β* and *β*_2,std_ were run and reported but, we only discuss *β*_2,std_ since it consistently outperforms *β*). *D*_ng_ is the sum of the correlation of the test variant with each neighboring variant in the window. Both the new method, LD-ABF and *D*_ng_ require phase data. So, power analysis includes haplotypes (to allow for LD-ABF and *D*_ng_ to be fit) and cross species data are generated as well (to allow for *β*_2,std_ and B2 to be fit). While not a comprehensive comparison of all available methods, our analysis draws on recent studies that demonstrate the comparable or superior performance of these methods (Siewert and Voight 2017, 2020; Tennessen and Duraisingh 2021). Additionally, our study highlights a range of conceptual approaches. In order to evaluate the methods, we calculated the area under the curve (AUC) and examined precision, recall, and F1 score at a false positive rate (FPR) of 5%. The F1 score is a measure of accuracy and is the harmonic mean of precision and recall (supplementary fig. S2, Supplementary Material online and supplementary table S1, Supplementary Material online). When the balancing selection variant is more recent in origin, the younger set, the improvement in predictive performance is greater for LD-ABF relative to the other methods: LD-ABF with AUC = 94.4% and F1 = 80.8%; *D*_ng_ with AUC = 92.4% and F1 = 73.4%; Tajima's *D* with AUC = 91.9% and F1 = 71.9%; *β*_2,std_ with AUC = 90.5% and F1 = 68.0%; B2 with AUC = 79.5% and F1 = 57.7%; and HKA with AUC = 68.4% and F1 = 39.6%. Although the classic Tajima's *D* appears third best in several cases, its performance appears inconsistent. For example, when examining younger variants at an equilibrium frequency of 25%, LD-ABF has an AUC = 93.4% and F1 = 76.8%, where Tajima's *D* has an AUC of 82.1% and F1 = 37.9%, corresponding to an AUC improvement of 11.3% and F1 improvement of 38.9% with our new method. All of the methods appear to perform best for variants of more ancient origin. For example, in the simulations where the balancing selection variant appears 250,000 generations before completion at an equilibrium allele frequency of 50%, the LD-ABF appears to perform best based on AUC and F1: LD-ABF with AUC = 98.3% and F1 = 93.3%; *D*_ng_ with AUC = 97.4% and F1 = 90.5%; *β*_2,std_ with AUC = 96.9% and F1 = 88.8%; Tajima's *D* with AUC = 96.3% and F1 = 86.8%; B2 with AUC = 92.7% and F1 = 83.9%; and HKA with AUC = 83.0% and F1 = 64.3%. Generally, most of the methods other than HKA appear to perform well. Tajima's *D* does better toward an equilibrium frequency of 50% and worse at lower and higher frequencies; whereas *D*_ng_ appears to show the flip performance, it performed better away from an equilibrium frequency of 50% and worse around 50%. *D*_ng_ appears to be the pretty comparable to LD-ABF away from MAF of 50%, for example in the older set at MAF of 25 they are both effective predictors with nearly identical AUC LD-ABF = 98.1% versus *D*_ng_ = 98.2% and F1 for both 92.3%. They are similar for other settings as well. LD-ABF and *D*_ng_ are constructed in similar manners to leverage phased samples and perform comparably, although LD-ABF tends to do noticeably better for more recent subtler signal and around MAF of 50%. In “recent” balancing selection (supplementary fig. S2, Supplementary Material online and supplementary table S1, Supplementary Material online), LD-ABF appears to outperform the *D*_ng_ more consistently, showing up to 4.1% (LD-ABF = 67.4% and *D*_ng_ = 63.3%) improvement in AUC and 7.0% (LD-ABF = 19.7 and 12.7) improvement in F1 at an equilibrium frequency of 50%.The B2 and BetaScan methods utilize cross species inference and perform well with older selection, but they still consistently underperform LD-ABF. This may denote the limitations of cross species selection analysis, especially for detecting more recent evolutionary events (Asthana et al. 2005). Both of these tests, B2 and *β*_2,std_, are likely to do better if the variant under selection is a transspecies polymorphisms. It should be noted that B2 allows for adaptive window sizes, which would likely improve its performance, but for consistency we restricted it in this analysis. For the scenario with varying window sizes (supplementary table S2, Supplementary Material online supplementary fig. S3, Supplementary Material online), at small window size of 100 bp all methods do poorly, and it appears the granularity of test windows are not appropriate for any test. While a window size of 500 bp is a bit worse than 1,000 bp, they are similar; however, when extending further to 5,000 bp there's a noticeable drop in performance, again this seems really consistent across all of the methods. Generally, the optimum window size appears around 1,000 bp consistent with the previous literature (Siewert and Voight 2017, 2020). LD-ABF at a window size of 1,000 performs best across all methods and window sizes.

Among the compared methods (Tajima's *D*, HKA, *D*_ng_, *β*, *β*_2,std_, and B2), LD-ABF stands out as the most robust, consistently predicting signals of balancing selection with the top or within 0.1% of the top of the AUC for each scenario. Moreover, LD-ABF performs the best or second best in terms of accuracy in the replicated set of simulation scenarios adapted from Siewert and Voight. Other methods have more variability depending on the scenario. Tajima's *D* and HKA have been shown to be outperformed by newer methods (Bitarello et al. 2023) where here this is especially seen with HKA. Further, all methods appear to either explicitly or implicitly take local SNP density into account in testing for selection; interestingly, this dependence on SNP density indicates that all tests are likely to be similarly hindered by real data in settings of limited or missing variants in large part due to platform limitations which we explore next.

### Genome-Wide Scan for Balancing Selection in Clinical Trios

**Fig. 2.**
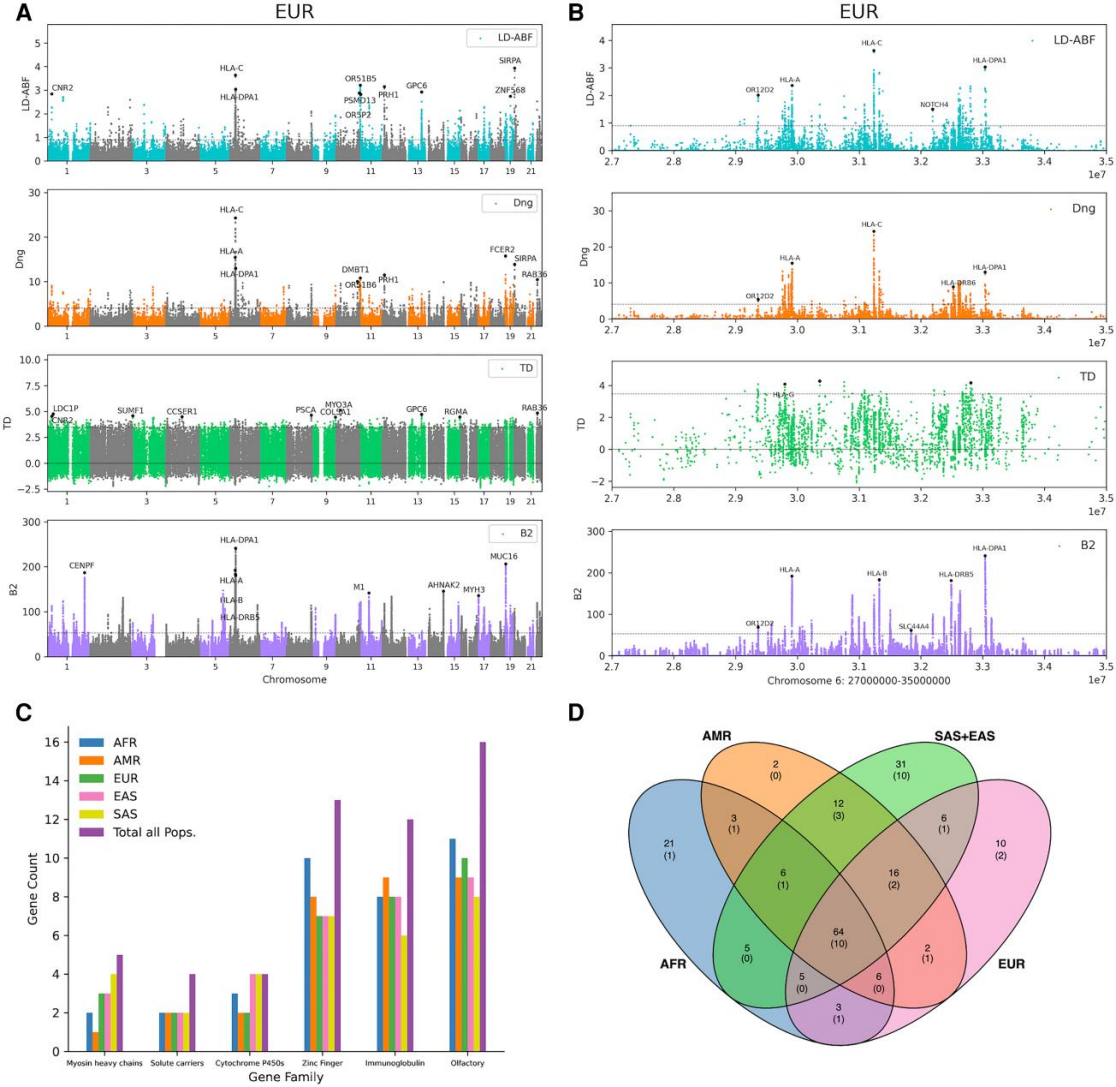
Genome-wide scan for balancing selection in clinical samples and gene patterns. Clinical samples were clustered based on 1KGP super-populations: African (AFR), American (AMR), East Asian (EAS), Southern Asian (SAS), and European (EUR). Genome-wide scans were performed within population to detect balancing selection, here in A) EUR genome wide comparing LD-ABF, *D*_ng_, Tajima's *D*, and B2 (other populations shown in supplementary fig. S4, Supplementary Material online) and B) a zoomed in plot across the MHC with class I and II HLA genes in the EUR clinical samples with different test statistics. The top ten peaks (where 1 Mb around a peak are ignored to determine subsequent peaks) are denoted with a dot and gene label, when it falls within a gene. Each statistic is plotted along with the line denoting the top 99.9% percentile for that test. Looking across the entire MHC, there appears to be several clusters of balancing selection signals centered around HLA genes. Three of these clusters (1. HLA-C, HLA-B; 2. HLA-DRB1, HLA-DQA1, HLA-DQB1; and 3. HLA-DPA1, HLA-DPB1) are separated by previously noted recombination hotspots (Cullen et al. 1997; Miretti et al. 2005; Nordin et al. 2020). Then restricting to the top 100 peaks, where LD-ABF scores in the immediate 1 Mb window around a peak are ignored to determine subsequent peaks, within each population is intersected with different C) HGNC gene families to get gene counts and the D) Venn diagram of unique and shared top 100 peak genes between populations with the two Asian populations combined with novel gene counts shown in parenthesis.

First, we analyzed 468 clinical samples from the Children's Hospital of Philadelphia (CHOP) with SNP array data and matching high coverage whole exome sequencing, including 334 trios (Table 1). By using clinical trios, there is improved phasing for children because when the sequence of the parents is known, then the child's haplotypes can more directly be inferred. This results in higher accuracy of phasing, and in turn test statistics, relative to computational phasing using software leveraging population level reference panels. Related individuals may have the potential to bias the scans; to prevent this, all individuals related to the proband were removed following phasing. Filtering on mapping quality, coverage, segmental duplications, repeats, allelic transmission disequilibrium, and other quality control was performed on samples (details in Methods). The clinical samples were phased using SHAPEIT2 and then combined using 1000 Genomes Project (1KGP) (Auton et al. 2015) super-populations in order to cluster into ancestral super-populations based on PCA (supplementary fig. S1, Supplementary Material online) (Auton et al. 2015) (see Methods). Afterwards the 1KGP samples were removed from the subsequent analysis. The 1KGP and PCA were used to assign CHOP samples to super-populations and these CHOP super-populations were analyzed separately. The super-populations designated here do not represent distinct local genetic populations, as they may possess unknown levels of substructure, thereby imposing limitations on the analysis. The choice of super-populations stems from the constrained availability of larger sample sizes from more geographically specific genetic ancestral groups. Ideally, a more comprehensive dataset comprising larger sample sizes from well-defined, localized populations would enhance the robustness of the analysis. By using LD-ABF, *D*_ng_, Tajima's *D*, and B2 were calculated genome wide for each super-population to determine where different balancing selection events occurred and in what super-populations (Fig. 2A and B, supplementary fig. S4, Supplementary Material online). These three additional methods beyond the new test statistic were chosen because Tajima's *D* is a classic statistic widely used for decades, *D*_ng_ is the most similar in construction, and B2 compares against closely related species, chimps. Although LD will dissipate further away from a selection event, there is some spread beyond the immediate window to neighboring regions. To identify unique selection events, when a local peak was identified for each test statistic, bases within a set neighborhood were excluded from additional peak determination. To be conservative in avoiding double counting peaks within long extended LD, the analysis was first performed using neighborhoods of 1 megabase (Mb) around the highest local scores. A follow-up analysis was then performed using 100 kb neighborhoods to detect peaks at a finer granularity (supplementary Online Data, Supplementary Material online). To further validate new selection signatures identified in genes that were not previously known as being associated with selection, a minimum of 50% of the polymorphisms found in the 1 kb region of the peak in the clinical trios needed to also be found in the Pangenome samples. This helps remove regions with possibly mappability issues. For the clinical trios, within each super-population labeled group, coordinates of the 100 highest peaks were used to identify candidate genes under balancing selection (supplementary Online Data, Supplementary Material online). Although LD-ABF is closely related to other statistics and could also be approximated to be asymptotically chi-squared distributed (Methods), instead for LD-ABF and comparator methods we investigate signal peaks to better isolate strong patterns of possible evolutionary events. Among these, 64 genes were shared across super-populations (Fig. 2D), including key HLA genes. Furthermore, we investigated the top 10 peaks of each super-population in detail (Fig. 2A and supplementary tables S3 to  S6, Supplementary Material online).

When comparing the methods, it was found that the top 10 peaks identified by each test statistic typically overlapped with at least one other method. However, the rank of the overlapping peak may be relatively lower, possibly outside of the top 10, but still within the top 100 (supplementary tables S5 and S6, Supplementary Material online). For instance, for at least one super-population *OR2T4*, *OR51F1*, *GBP4*, *OR51Q1*, *MMP26*, *ZNF280A*, *SP110*, *UGT1A5*, *UGT1A6*, *UGT1A7*, *OR52E6*, *ZNF568*, *UGT1A8*, *UGT1A10*, *UGT1A9*, and *FNDC1* fall in a top 10 peak for LD-ABF and are not in the top 10 peaks of any other method; however, all of these were in the top 100 peaks of at least one other method.

Focusing on the top peaks using LD-ABF, the top peak for the AFR super-population is in *OR51B6* of the olfactory receptor (OR) gene cluster; for the SAS super-population, the top peak appears in *HLA-DPA1*, an MHC class II gene; and for AMR, EUR, and EAS super-populations, the top peak is in *SIRPA*, which encodes for a signal regulatory protein of the immunoglobulin superfamily. In fact, peaks in *SIRPA* rank among the top 4 for each super-population. When looking at the other methods, it is also picked up as a top 100 peak at least one super-population. Among all top 100 peaks across super-populations detected by LD-ABF, a total of 38 genes not previously known to be under selection (Table 2 and supplementary table S7, Supplementary Material online and Fig. 2D) (Hayeck et al. 2024) were tagged by signals of balancing selection (Table 2) (Hayeck et al. 2024), including 10 shared between all super-populations (Fig. 2D): *TRMT9B*, *COL5A1*, *SNRPN*, *OR1S1*, *QRICH2*, OR2T4, *SNHG14*, *HCG20*, *KRTAP10-9* and *PGAP6*. Of the 38 new genes with selection signals, 14 were only found by LD-ABF: *AADACL3*, *ARHGEF19*, *CCDC50*, *CFAP61*, *CRNKL1*, *FAM214A*, *KCNQ2*, *LRRC32*, *OR13G1*, *OR52Z1*, *PAX2*, *PCARE*, *CYP4F2*, and *MRGPRX4*, whereas the remaining 24 were replicated by two or more methods. The 38 candidate genes identified resulted from inferences made at the super-population level. It is important to acknowledge that signatures of fine-scale local adaptation may resemble those identified here. While this is less probable in, for example, European samples, it is not improbable in Africa, where high differentiation and local adaptation may be more common. In all instances, it is important to recognize this as a potential limitation of the results.

**Table 2:** Novel signals of balancing selection in genes from genome-wide scan of clinical trios

Genes	LD-ABF					*D*_ng_					B2					TD				
	AF	AM	EU	EA	SA	AF	AM	EU	EA	SA	AF	AM	EU	EA	SA	AF	AM	EU	EA	SA
*AADACL3*	…	92	94	58	…	…	…	…	…	…	…	…	…	…	…	…	…	…	…	…
*ARHGEF19*	…	…	…	…	77	…	…	…	…	…	…	…	…	…	…	…	…	…	…	…
*CCDC50*	92	89	…	…	…	…	…	…	…	…	…	…	…	…	…	…	…	…	…	…
*CFAP61*	…	…	49	…	…	…	…	…	…	…	…	…	…	…	…	…	…	…	…	…
*CRNKL1*	65	91	49	96	44	…	…	…	…	…	…	…	…	…	…	…	…	…	…	…
*FAM214A*	…	…	…	78	…	…	…	…	…	…	…	…	…	…	…	…	…	…	…	…
*KCNQ2*	…	…	…	69	…	…	…	…	…	…	…	…	…	…	…	…	…	…	…	…
*KRTAP10–9* ^a^	51	49	55	47	39	…	…	…	…	…	…	…	…	…	…	…	…	…	…	…
*LRRC32*	…	76	88	…	…	…	…	…	…	…	…	…	…	…	…	…	…	…	…	…
*MRGPRX4*	…	…	…	53	81	…	…	…	…	…	…	…	…	…	…	…	…	…	…	…
*OR13G1*	…	…	…	…	85	…	…	…	…	…	…	…	…	…	…	…	…	…	…	…
*OR1S1* ^a^	…	12	…	23	18	…	…	…	…	…	…	…	…	…	…	…	…	…	…	…
*PAX2*	57	…	100	…	…	…	…	…	…	…	…	…	…	…	…	…	…	…	…	…
*PCARE*	…	…	64	45	53	…	…	…	…	…	…	…	…	…	…	…	…	…	…	…
*SELENOO* ^a^	…	…	…	64	71	…	…	…	…	…	…	…	…	…	…	…	…	…	…	…
*OR52Z1* *	96	…	…	…	91	…	…	…	…	…	…	…	…	…	…	…	…	…	…	…
*CYP4F2* *	…	…	…	…	86	…	…	…	…	…	…	…	…	…	…	…	…	…	…	…
*HLA-H* *	…	…	83	…	…	8	8	8	9	32	…	…	…	…	…	…	…	…	…	…
*TRMT9B*	58	66	56	46	38	…	…	…	81	…	…	…	…	…	…	…	…	…	…	…
*ZNF778* *	65	…	…	…	…	…	…	…	…	…	…	…	54	…	69	…	…	…	…	…
*CMYA5*	…	…	…	72	…	…	…	…	…	…	29	20	55	11	10	…	…	…	…	…
*KLHDC7A*	…	…	…	…	70	…	…	…	…	…	…	50	77	…	…	…	…	…	…	…
*MYH3*	…	83	…	100	76	…	…	…	…	…	31	16	10	20	17	…	…	…	…	…
*QRICH2*	39	53	53	24	56	…	…	…	…	…	…	…	100	…	…	…	…	…	…	…
*ZNF45*	90	50	36	…	37	…	…	…	…	…	…	…	43	…	…	…	…	…	…	…
*HCG20*	26	…	…	…	…	…	…	…	…	…	…	…	…	…	…	79	…	…	…	…
*KRTAP7-1*	…	…	…	95	93	…	…	…	…	…	…	…	…	…	…	…	…	74	…	83
*ONECUT2*	93	57	…	41	…	…	…	…	…	…	…	…	…	…	…	…	28	…	79	…
*PLEKHG4B*	…	99	83	81	62	…	…	…	…	…	…	…	…	…	…	…	80	…	…	…
*SNHG14*	55	46	45	38	48	…	…	…	…	…	…	…	…	…	…	…	…	99	…	…
*SNRPN*	55	46	45	38	48	…	…	…	…	…	…	…	…	…	…	…	…	99	…	…
*OR10G9*	…	…	…	71	…	95	…	…	94	…	25	49	…	…	37	…	…	…	…	…
*OR2T4*	15	10	57	5	4	21	42	28	18	26	…	51	…	39	…	…	…	…	…	…
*PGAP6*	99	41	35	73	29	74	43	42	…	24	…	…	70	…	…	…	…	…	…	…
*PLEC*	…	…	71	…	…	…	…	31	…	…	…	13	31	68	97	…	…	…	…	…
*ADGRF2*	…	87	…	66	…	94	…	…	…	…	…	…	98	…	…	…	39	48	…	…
*COL5A1*	22	34	30	20	22	89	…	19	69	16	…	74	37	83	69	42	81	10	13	30
*HCG17* *	…	72	…	63	62	…	27	…	25	36	…	83	67	44	74	…	…	…	3	30

The set of new selection signals not previously found in these genes before with their corresponding peak rank for each statistic using 1 Mb peak finding listed for the given statistic and corresponding population. Genes marked with the superscript “a” were also recognized as part of the top 100 peaks using methods other than LD-ABF, where peak detection was conducted with a different window size of 100 kb.

^*^Denotes a gene that corresponds previously unknown gene under selection but, found using LD-ABF with the other peak finding window size of 100 kb.

As expected, several other top peaks are in HLA genes. In fact, peaks in *HLA-C*, and *-DPA1* are top 10 for LD-ABF in at least one super-population and shared among the top 100 peaks across all super-populations. Additionally, all three are captured by the top peaks of every method except for Tajima's *D* (supplementary tables S5 and  S6, Supplementary Material online). Looking across the entire MHC (Fig. 2B), the three newer statistics (LD-ABF, *D*_ng_, and B2) effectively identify clear and consistent peaks clustering throughout the MHC, separating the class I, II, and III HLA genes. Their relative rankings, however, vary from super-population to super-population and across statistics. In the super-populations with the largest sample sizes, EUR and AMR, the highest HLA peak is found in *-C*, while for AFR, EAS, and SAS, the highest HLA peak is found in *-DPA1*. In total, 13 HLA and other immunoglobulin superfamily genes are marked by top 100 LD-ABF peaks across all super-populations (Fig. 2B and supplementary table S8, Supplementary Material online). Immune related and cell surface receptor signaling genes are expected candidates for balancing or positive selection as their functionality is often directly tied to environmental interactions. Consistent with this, we also detected LD-ABF peaks across 16 OR genes and several taste receptor genes (Fig. 2B and supplementary Online Data, Supplementary Material online). In addition, peaks were also seen across members of several other gene families (Tweedie et al. 2021), including zinc fingers (ZF) (13), cytochromes (4), solute carriers (4), and myosin heavy chains (5) (Fig. 2C).

Bases scoring in the top 99.9% LD-ABF genome wide were then intersected with known GWAS catalog significant SNPs (Buniello et al. 2019) to find overlap between strong signals of selection and known disease associated variants (Table 3 and supplementary table S9, Supplementary Material online). Using 99.9% coincides with a more restrictive threshold than the cutoff for top 100 peaks while still allowing for consideration of multiple variants of interest within the same peak. Many of the SNPs overlapping high LD-ABF scores were found to be associated with blood and immune related traits. Among these, the strongest signal for EAS was at rs17855611 in *SIRPA* associated with blood protein levels, and for SAS, at rs1126506 in HLA associated with Anti-rubella virus IgG levels, influencing the immune response against the rubella virus. In contrast, the strongest signals in AFR, AMR, and EUR were seen in *OR51B6*, which corresponds to rs5006884 with known association to fetal hemoglobin (HbF) levels in sickle cell anemia, a classical example of balancing selection driven disease (Solovieff et al. 2010). This SNP lies upstream of the β-globin locus control region and is in close proximity to several candidate enhancers of *HBG2* (Safran et al. 2021), which codes for the gamma-2 subunit of HbF.

**Table 3:** Top balancing selection signals in clinical samples at GWAS significantly associated SNPs

Pop	Chr	ID	LD-ABF	Genes	Disease/trait	Sequence context
AFR	11	rs5006884	0.69	*OR51B6*	Fetal hemoglobin levels	Missense variant
	6	rs9277354	0.43	*HLA-DPB1*	Antineutrophil cytoplasmic antibody-associated vasculitis	Frameshift variant
	6	rs9277356	0.43	*HLA-DPB1*	Response to hepatitis B vaccine	Missense variant
	6	rs1126506	0.42	*HLA*	Anti-rubella virus IgG levels	Splice region variant
	20	rs17855611	0.39	*NR*	Blood protein levels	Missense variant
	2	rs4988958	0.36	*IL1Rl1*, *IL1RL2*, *IL18R1*	Asthma (childhood onset)	Synonymous variant
	6	rs1042151	0.29	*HLA-DPB1*	Aspirin exacerbated respiratory disease in asthmatics, severe aplastic anemia	Missense variant
	6	rs520692	0.26	*C4A*	Feeling worry	Missense variant
	19	rs602662	0.26	*FUT2*	Folate pathway vitamin levels, pediatric autoimmune diseases, vitamin B12 levels	Missense variant
	10	rs2249694	0.25	*CYP2E1*	Obesity-related traits	Intron variant
	6	rs2858331	0.24	*HLA-DQA2*	IgE levels	Regulatory region Variant
AMR	11	rs5006884	0.67	*OR51B6*	Fetal hemoglobin levels	Missense variant
	6	rs1126506	0.50	*HLA*	Anti-rubella virus IgG levels	Splice region variant
	6	rs9277354	0.49	*HLA-DPB1*	Antineutrophil cytoplasmic antibody-associated vasculitis	Frameshift variant
	6	rs9277356	0.49	*HLA-DPB1*	Response to hepatitis B vaccine	Missense variant
	6	rs2894204	0.47	*NR*	Waist–hip ratio	Intron variant
	20	rs17855611	0.43	*NR*	Blood protein levels	Missense variant
	2	rs4988958	0.38	*IL1Rl1*, *IL1RL2*, *IL18R1*	Asthma (childhood onset)	Synonymous variant
	6	rs9264638	0.38	*HLA-C*	Beta-2 microglubulin plasma levels	Intron variant
	1	rs4525	0.37	*F5*	Blood protein levels	Missense variant
	6	rs1050451	0.35	*HLA-B*, *HLA-C*	IgG galactosylation phenotypes (multivariate analysis)	Missense variant
	1	rs4524	0.34	*F5*	Venous thromboembolism	Missense variant
	6	rs34794906	0.34	*HLA-C*	Reticulocyte count	Synonymous variant
	6	rs2516703	0.31	*HCG17*	Itch intensity from mosquito bite	Intron variant
	19	rs602662	0.30	*FUT2*	Folate pathway vitamin levels, pediatric autoimmune diseases, vitamin B12 levels	Missense variant
	6	rs1042133	0.28	*HLA-DPB1*	Monocyte count	Missense variant
EUR	11	rs5006884	3.01	*OR51B6*	Fetal hemoglobin levels	Missense variant
	2	rs4988958	2.27	*IL1Rl1*, *IL1RL2*, *IL18R1*	Asthma (childhood onset)	Synonymous variant
	6	rs9277354	2.14	*HLA-DPB1*	Antineutrophil cytoplasmic antibody-associated vasculitis	Frameshift variant
	6	rs9277356	2.14	*HLA-DPB1*	Response to hepatitis B vaccine	Missense variant
	6	rs1126506	2.13	*HLA*	Anti-rubella virus IgG levels	Splice region variant
	17	rs1864325	1.69	*MAPT*	Lumbar spine bone mineral density	Intron variant
	17	rs12373142	1.67	*SPPL2C*	Chronic obstructive pulmonary disease	Missense variant
	6	rs2894204	1.63	*NR*	Waist–hip ratio	Intron variant
	6	rs1050451	1.63	*HLA-B*, *HLA-C*	IgG galactosylation phenotypes (multivariate analysis)	Missense variant
	19	rs602662	1.57	*FUT2*	Folate pathway vitamin levels, pediatric autoimmune diseases	Missense variant
	20	rs17855611	1.56	*NR*	Blood protein levels	Missense variant
	8	rs56117011	1.51	*PLEC*	Post-bronchodilator FEV1	Synonymous variant
	8	rs35916068	1.51	*PLEC*	Post-bronchodilator FEV1	Synonymous variant
	6	rs520692	1.50	*C4A*	Feeling worry	Missense variant
	6	rs9264638	1.50	*HLA-C*	Beta-2 microglubulin plasma levels	Intron variant
	1	rs4525	1.47	*F5*	Blood protein levels	Missense variant
	8	rs55646585	1.44	*PLEC*	Post-bronchodilator FEV1	Synonymous variant

SNPs that are both found to be significantly associated with a phenotype in the GWAS catalog and also have a strong selection signal in the top 99.9%. The results for clinical samples in the EUR, AFR, and AMR populations are here with the EAS and SAS populations continued in supplementary table S9, Supplementary Material online.

The analysis of the SNP array and exome sequencing data indicated strong selection signatures in genes associated with the immune and sensory systems, generally showing good consistency across methods (less so with Tajima's *D*). The presence of strong balancing selection in the MHC region was expected, but the SNP array analysis revealed inconsistent signals in the HLA genes among various super-populations. To further investigate this inconsistency, we look to targeted genotyping of the HLA genes.

#### Detailed Investigation of HLA Genes Using High-Quality Typing

Diversity in HLA genes have long been recognized as key examples of balancing selection (Parham 2005; Barreiro and Quintana-Murci 2010; Lenz et al. 2016). Moreover, even though the MHC accounts for only 0.16% of the genome, 39% of all GWAS SNPs that overlapped top LD-ABF scores occurred within the MHC. So, despite accounting for a fraction of a percent of the genome, over 2% of GWAS variants are found in the MHC. Furthermore, when testing the odds ratio of comparing the top 99.9% under balancing selection versus not under selection (GWAS variants within the MHC to those outside the MHC), there is an enrichment of over 30-fold (Fishers exact *P* < 10^−10^). Despite these observations and its profound importance to the fields of immunology, immunogenetics, and evolutionary biology, detailed follow-up and characterization of the MHC and its HLA genes have been limited. Fortunately, due to the importance of HLA matching for avoiding rejection and graft versus host disease in organ and stem cell transplants, detailed typing of selective HLA genes is routinely performed in the clinical setting (Petersdorf et al. 2014; Wiebe et al. 2018; Shieh et al. 2021). Taking advantage of this, we utilized high-resolution HLA typing data from the IHIW to take a closer investigation of balancing selection across these genes. This dataset consists of over 3,500 samples, each providing 2 alleles per HLA gene typed at 4 field resolution and represents a diverse set of world populations (Methods).

**Fig. 3.**
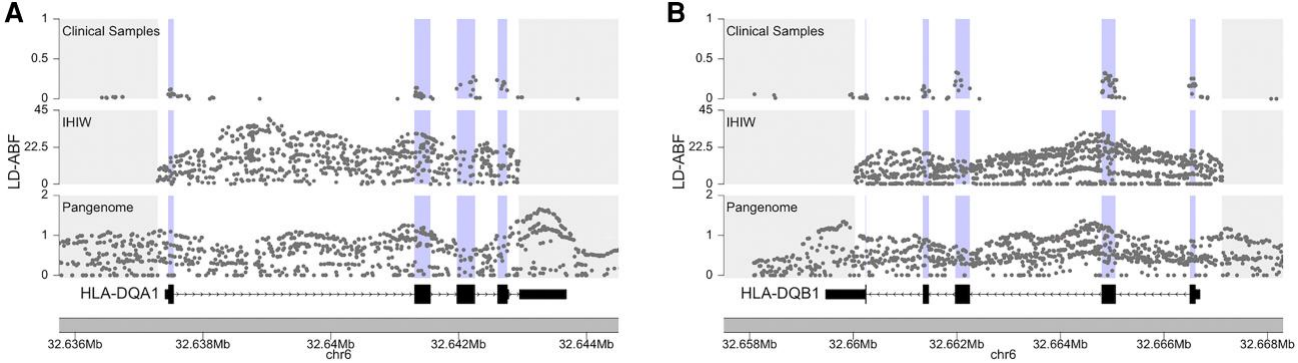
Balancing selection in HLA-DQA1 and DQB1 comparing the clinical samples, 17th IHIW, and Pangenome. LD-ABF scores over A) DQA1 and B) DQB1 from independent samples of African ancestry are compared. Exonic regions are highlighted in purple. Exons are shaded in blue. The relative magnitude of the LD-ABF signals reflects the sample size of the population as any standard test statistic would. Clinical samples used a combination of SNP array and high coverage exome sequencing, the IHIW came data are high-resolution HLA genotyping, and the Pangenome are whole-genome long-read data. sequencing data.

**Fig. 4.**
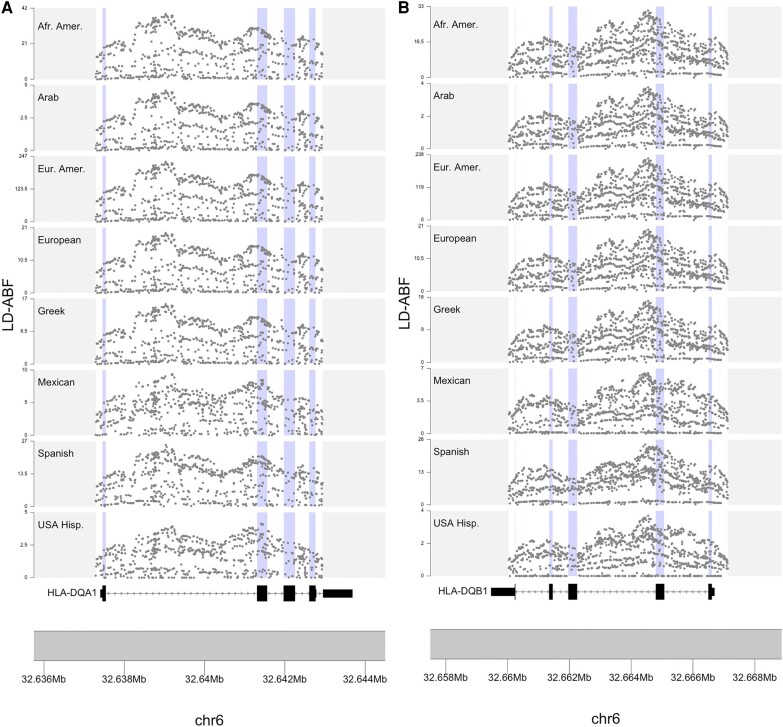
Comparison of LD-ABF across IHIW populations for HLA-DQA1 and HLA-DQB1. Detailed look at consistent patterns of LD-ABF across the HLA-DQ* genes (with the largest signal in the IHIW samples) and eight world populations. Exons are shaded in blue. Different scales are proportional to the relative within-population sample sizes.

Strikingly, the strongest LD-ABF signals were consistently observed in -*DQA1*, -*DQB1*, and *DRB1* across all IHIW populations and in Pangenome samples (Figs. 3 and 4 and supplementary figs. S6 to S10, Supplementary Material online). This contrasts with scans of the clinical samples, where either *-C* or *-DPA1* were the top hits across the MHC depending on the population. Furthermore, within each HLA gene, consistent patterns of balancing selection were observed across all populations, including strong signals in the intronic regions (Fig. 4 and supplementary figs. S6 to S9, Supplementary Material online). Similar observations are made when using other methods (not displayed here) where inferior sequencing coverage seems to diminish detection ability and lead to less reliable results, underscoring the significance of sequencing quality in terms of inferring selective signals. Not surprisingly, these regions with the highest LD-ABF scores corresponds to regions with the highest concentration of GWAS trait associated SNPs. A review of SNPs overlapping top LD-ABF scores revealed associations with traits like red blood cell count, leukemia, autism, schizophrenia, and asthma (supplementary table 10, Supplementary Material online). The sequence context of the majority of these SNPs was either intronic or missense, which is expected in the context of balancing selection; as opposed to nonsense or loss of function variants, which would be expected in settings of purifying selection (Petrovski et al. 2013; Karczewski et al. 2020). Looking over the exons of HLAs, the highest LD-ABF signals for both *-DQA1* and *-DQB1* were found in exon 2, which encode for extracellular domains key to peptide presentation. Diversity in the peptide-binding pocket ensures effective immune recognition of a wide range of foreign pathogens, in tune with mechanisms driving balancing selection.

### Validation With Long-Read Pangenome Samples

To further validate LD-ABF testing and assess the impact of sequencing, we next looked at whole-genome HiFi PacBio sequencing data gathered by the Pangenome Consortium. Along with understanding how quality and coverage impact testing, these high-quality long-read samples are expected to help remove artifacts introduced by inaccurate assembly and alignment of other platforms. This is especially applicable for genomic regions of high homology and complexity that are difficult or impossible to properly align and map when using short-read sequencing, including the MHC. Although these samples offer superior sequencing quality, the largest population consists of just 23 African samples; so, they are presented here predominantly for selective verification and not as part of the broader analysis. The other Pangenome populations were too small to perform statistical inference (Methods).

**Fig. 5.**
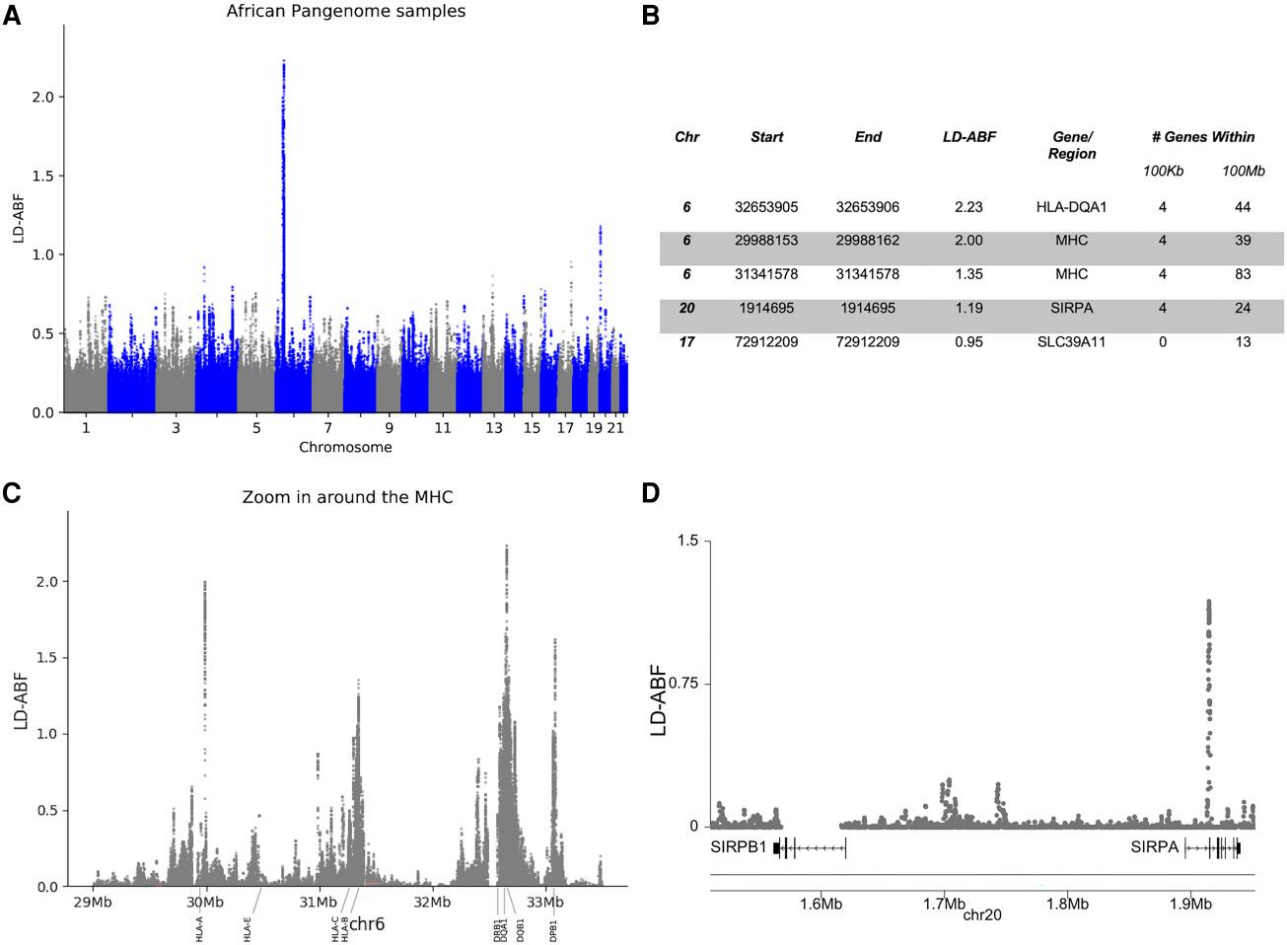
Signals of balancing selection detected in the Pangenome samples. LD-ABF scores calculated from long-read HiFi PacBio data are shown a) genome wide and with a B) table detailing the top 5 LD-ABF peaks C) zoom in around the MHC D) zoom in around the SIRP genes.

A key reason for exploring the Pangenome samples was to further study the *SIRP* region, which demonstrated surprisingly strong signal in the clinical samples both from LD-ABF and other methods (supplementary table S6, Supplementary Material online). The magnitude of the *SIRPA* LD-ABF signal is second only to the MHC in the Pangenome data, confirming strong balancing selection (Fig. 5A). However, the observed high signal of balancing selection in clinical samples at *SIRPB1* was not replicated in the long-read samples and is likely artifactual, due to platform limitations (Fig. 5D). The signal was much smaller over the Pangenome and it also did not pass filtering criteria for matching at least half of the polymorphisms found in the clinical samples. There is a known copy number variation in *SIRPB1* potentially causing mapping or alignment issues that likely led to strong misleading signals in the clinical samples (Royo et al. 2018) (supplementary fig. 12, Supplementary Material online).

Beyond the MHC and *SIRPA*, the top 100 peaks in the Pangenome samples (supplementary online data, Supplementary Material online) included *OR51B5*, *MYO3A*, and *OR6J1*, which were also found to be top hits for clinical samples along with other studies (Asthana et al. 2005; Andrés et al. 2009; DeGiorgio et al. 2014; Bitarello et al. 2018).

Signals of LD-ABF in HLA genes from African populations were compared across datasets. As the scale of LD-ABF signal is a function of sample size, for this comparison, we focus on the relative peaks and shapes of the distributions as opposed to the absolute LD-ABF scores. Since the data for the clinical samples are limited by the exome sequencing and variants on the SNP arrays, it became clear how incomplete the data were as compared to the IHIW and the Pangenome (Fig. 3 and supplementary fig. 11, Supplementary Material online). The patterns of LD-ABF from the IHIW samples largely matched those of the Pangenome samples, with the exception of a problematic subregion within the *HLA-DRB1* (supplementary fig. 10, Supplementary Material online). A dramatic peak centered on intron 5 of -*DRB1* seen in the IHIW dataset was completely absent in the Pangenome analysis. This intronic region of *DRB1* with the strong signal in the IHIW samples is known to contain an Alu and a LINE, long transposable elements that hinder accurate mapping of shorter sequencing reads. The known structural variation and disparate repeat elements in this region of *DRB1* in short-read settings can result in issues when performing multiple sequence alignment and therefore likely causes artifactual LD. These challenges are reconciled when using the Pangenome and consequently the false LD-ABF peak dissipated across the Pangenome samples. The Pangenome, and long-read sequencing in general, offers an invaluable resource for reconciling such artifacts while also providing dramatic replication of surprisingly strong signals, like that seen in *SIRPA*.

## Discussion

LD-ABF improves detection of evolutionary selective pressures by evaluating both the magnitude of LD and the density of variation making direct inference on phased haplotypes. LD-ABF requires known haplotypes and like other test statistics is most effective when there are large sample sizes with good coverage. Leveraging LD-ABF, we analyzed three independent datasets representing different sequencing technologies, each with unique advantages and limitations. Our comparative analysis revealed that sequencing strategies significantly influence the detection of selection patterns, implying that any population genetics study relying on polymorphism density and LD modeling may introduce biases due to sequencing limitations.

The objective was to develop a model that utilizes phased haplotypes to enhance the prediction of selection signatures. Analysis can be challenging in regions where phased data are sparse or some SNPs are in perfect LD, which are both common occurrences. However, incorporating these factors directly into the model can improve statistical inference, which is precisely what LD-ABF achieves. Penalization methods offer valuable solutions to address issues related to separation (i.e. perfect LD) and data sparsity, and they have foundations in both frequentist and Bayesian approaches. Penalty functions are commonly employed to drive parameter estimates toward zero by incurring a cost for including parameters in the model. These methods are particularly effective in high-dimensional settings that involve parameter selection. In frequentist settings, this corresponds to maximizing likelihood estimation, $\hat{\beta }=\mathrm{argmax}\{l(\beta )+r(\beta )\}$, where l(β) is the log likelihood and r(β) is the penalty. A common example is the Lasso penalty, r(β)=λ|β|, which is the absolute value of effect estimates with *λ* as a tuning parameter that modulates the coefficients by adding a cost for including the term in the model.

Greenland and coauthors (Greenland 2003, 2007; Greenland and Mansournia 2015; Mansournia et al. 2018) have extensively investigated penalized functions and their Bayesian equivalents. Greenland proposes using a class of loss functions proportional to the information matrix, $r(\beta )=\ln(\left|I(\beta )\right|{)}^{m}$, where *I* is the fisher information and *m* is a hyper parameter. In particular, a form of this penalty function in binary outcome settings is equivalent to employing logF priors and has been shown to mitigate bias and mean square error (MSE) in scenarios involving separation and data sparsity (Discacciati et al. 2015). Similar to the Cauchy or t-distribution, logF priors increase the tail weight or skewness of the prior distribution. The logF distribution provides heavier tails than a multivariate normal distribution but lighter tails than the Cauchy distribution. An additional advantage of using logF priors is that estimation with data augmentation techniques can be performed with calculations on the order of running a standard logistic regression model. Moreover, the logF prior belongs to the conjugate family for binomial logistic regression, making it a natural choice in such settings.

The MHC is a complicated region with notably strong LD that benefits from such analytical approaches. The MHC is a genomic region of particular interest both from a medical perspective and in terms of understanding evolutionary pressures. Numerous studies have established connections between over 700 diseases or traits and the MHC, making it the genomic region with the highest number of associations of comparable size (Clark et al. 2015; Shieh et al. 2018). While the MHC represents around 0.1% of the genome, it corresponds to nearly 2% of all GWAS catalog associations (Buniello et al. 2019).

The MHC has been extensively studied as a prominent example of balancing selection (Parham 2005; Barreiro and Quintana-Murci 2010; Lenz et al. 2016), with a specific focus on class I genes exhibiting stronger selection (Alter et al. 2017). Agreeing with previous literature strong signal was seen of the class II region and even focusing in on the *HLA-D* gene clusters (Leffler et al. 2013; DeGiorgio et al. 2014; Teixeira et al. 2015; Siewert and Voight 2017; Bitarello et al. 2018; Meyer et al. 2018; Cheng and DeGiorgio 2020); however, to our knowledge other studies did not consistently revealed the strongest signals in *HLA-DQA1* and *-DQB1* across all populations as in this study, as supported by the IHIW and Pangenome data (Figs. 3 to 5 and supplementary figs. S6 to S10, Supplementary Material online). This discrepancy could be attributed to the utilization of low resolution typing or older SNP array data in earlier studies, including our own analysis of SNP array and exome sequence data. The more recent insights provided by the IHIW and Pangenome initiatives challenge these earlier observations.

In addition to emphasizing the robust balancing selection signals observed in class II HLAs, the IHIW data demonstrated strong signals within intronic regions of the HLA genes, while the Pangenome data revealed strong signals within intergenic regions of the MHC as well (Figs. 3 and 5, supplementary fig. 11, Supplementary Material online, and supplementary Online Data, Supplementary Material online). These regions, which have received limited attention in previous studies, were found to exhibit notable selection signatures. Many GWAS disease associated SNPs fall within these noncoding regions; our analysis here begins to offer some clues regarding the evolutionary forces that contributed to these polymorphisms. Although the clinical samples also showed strong signals across HLA genes, it alone would have missed much of these interesting intricacies due to the sparseness of the data, especially over introns and intergenic regions. Furthermore, the consistent patterns of balancing selection observed in the HLA genes across diverse populations in the IHIW data (supplementary figs. S6 to S10, Supplementary Material online) suggest the possibility of convergent evolution, a phenomenon previously documented in HLAs (O’Huigin et al. 2011; Creary et al. 2021).

Similar to the MHC, one of the strongest signals of balancing selection across the genome was observed in *SIRPA* within the clinical samples. This signal was further validated in the independent sample set of the Pangenome (Fig. 5). Previous studies (Bitarello et al. 2018; Tennessen and Duraisingh 2021) also noted selection around *SIRPA*, but the strength of the signal was not as pronounced as in this study, finding it as consistently a top 5th gene. Notably, in our study Tennessen's method, *D*_ng_, identified *SIRPA* as one of the top five genes across all clinical super-populations, similarly to LD-ABF. These findings suggest that discrepancies in rank ordering of *SIRPA* was due to sequencing platforms, potentially resulting in poorer coverage, and differences in sample sources may have influenced previous results.

SIRPα plays a crucial role as an inhibitory receptor for CD47 and is a key component of the “do-not-eat-me” signaling pathway, with potential implications in transplantation (Garcia-Sanchez et al. 2021). Similar to the HLA genes, the sequences encoding the extracellular domain of SIRPα exhibit the strongest signal. Interestingly, structural analysis revealed that most polymorphisms in *SIRPA* do not affect CD47 binding, unlike the variation observed in the complementary determining regions of the HLA molecules and immunoglobulins. Instead, these polymorphisms cluster away from the CD47 binding footprint and are believed to be under selection to minimize pathogen binding and manipulation of the “do-not-eat-me” signal (Hatherley et al. 2014).

In addition to HLAs and *SIRPA*, our analysis of top LD-ABF peaks across all super-populations (Table 2) revealed several other notable genes and gene families. OR genes formed the largest gene family under balancing selection, this is to be expected because the OR are responsible for the detection of odors. The ability to sense the environment detecting odors related to hazards, food, or social interactions, significantly influences the survival and adaptation of a species. Notably, both HLAs and ORs are thought to have diversified through gene duplications and consequently both families reside in regions of high gene density. These observations, along with the high homology among members of HLAs, ORs, and other gene families identified in our study suggests that balancing selection and gene duplications are often the result of similar evolutionary pressures.

Furthermore, our analysis highlighted additional genes and gene families that showed evidence of balancing selection. These included taste receptor genes (*TAS2R*), genes associated with psychoactive and anti-inflammatory responses (*CNR2*), zinc finger genes (*ZNF280A* and *ZNF568*) that serve as binding molecules with DNA and RNA, and several cytochrome P450 genes. The cytochrome P450 enzymes play a crucial role in drug metabolism and lipid synthesis, catalyzing a wide range of reactions (Sayers et al. 2019). The identification of these diverse gene families suggests that balancing selection operates on genes involved in various biological processes, reflecting the intricate interplay between evolutionary pressures and functional adaptations.

Upon analyzing the top 100 peaks (within 1 Mb and 100 kb neighborhoods) across all super-populations, we identified a total of 38 new selection signatures in genes that were not previously recognized to be under selection. Out of the 38 newly identified signals, the majority (24) were also detected by existing methods. However, several signals would have been missed without employing LD-ABF. Notably, 14 genes were solely detected by LD-ABF, including members of expected gene families such as olfactory receptor genes and keratin-associated genes. While these specific genes had not been previously recognized as under selection, other genes in the same gene families have been identified in prior studies, further supporting their significance in evolutionary processes. This work has several limitations that present opportunities for future investigations. Firstly, due to limited sample availability, we had to utilize 1KGP super-populations. However, for a more comprehensive understanding, it would be ideal to incorporate more distinct subpopulations in future studies and this may have impacted the detection of the 38 candidate genes. Splitting our EUR samples into subpopulations we saw similar signals of selection across the MHC and across HLA genes (supplementary fig. 14, Supplementary Material online), although we hasten that our population is not of an ideal size and representation of diverse populations to truly test that this is not an issue. We are currently working on follow-ups to characterize the relative divergence in selection across more granular subpopulations. Additionally, future research should aim to explore local adaptations at the sub-population level. Moreover, the current method focuses on selection within populations without explicitly testing for relative differentiation. Future work could expand the existing statistical framework to directly assess and compare such differences.

It is important to acknowledge that the datasets used in this study may have limitations related to sequencing quality and potential underrepresentation of certain populations. It is worth noting that the clinical samples were obtained from individuals visiting the Children's Hospital of Philadelphia for clinical assessment and were not specifically curated for the study of evolutionary selection. Therefore, the representativeness of these datasets should be considered.

The presence of extensive repeat regions, structural variations, ectopic recombination, and other complex sequences may introduce biases or generate artifact signals. The current analysis focuses on LD within a 1 kb window and does not examine long-range LD. To address these challenges, the utilization of long-read sequencing (Logsdon et al. 2020) will play an increasingly crucial role in deciphering the complexity of the MHC and other regions of the genome with high homology or extensive LD. The future advancement of studies using novel technologies, like long-read sequencing, with large sample sizes hold the promise to uncover selection signal in regions that have traditionally been overlooked due to sequence complexity (supplementary fig. 13, Supplementary Material online). Additionally, employing advanced mapping and alignment techniques, such as population reference graphs (Dilthey et al. 2016), can enhance the genetic characterization of diverse human populations (Eichler 2019). These methodological advancements, when coupled with tools like LD-ABF, will contribute to a better understanding of the impact of evolutionary pressures on genomic functionality.

In conclusion, the limitations associated with sequencing platforms, including low coverage or incomplete sequencing, as well as challenges in mapping and alignment within complex regions, can hinder the accurate detection of evolutionary selection. This is because all methods directly or indirectly assess polymorphism density and the strength of LD. Our study highlights that a smaller yet high-quality long-read sequencing datasets have the potential to offer a more comprehensive understanding of evolutionary patterns compared to larger datasets generated using alternative sequencing platforms. Furthermore, by utilizing LD-ABF in conjunction with a combination of sequencing technologies, we were able to enhance the identification of selection signals and uncover novel targets of selection. Moving forward, in addition to ongoing statistical methodological advancements, a cost-effective approach for comprehensive characterization of complex genomic regions may involve the strategic utilization of high-quality sequencing data and a carefully curated set of samples. This integrated approach has the potential to provide valuable insights into the intricate dynamics of evolutionary selection and improve our understanding of the genetic underpinnings of various traits and diseases.

## Materials and Methods

### Linkage Disequilibrium Approximate Bayesian Factor (LD-ABF)

The model aims to detect selection by testing for both level of linkage between the test variant with neighboring variants and density of polymorphisms around the test variant (Fig. 1). Phased individual level haplotype data was used to enable the clearest detection that the test variant of interest is in strong linkage with neighboring variants. To test for association between a given variant and neighboring variants first consider just testing the association between haplotypes of one variant versus one other neighboring variant. Take test variant $x_{i}$, $x_{i}=\{0,1\}$ where 0 corresponds to the major allele and 1 minor allele and *i* is the index for the individual, and a neighboring variant $y_{i}=\{0,1\}$, again corresponds to the major or minor allele. A logistic regression model is a natural choice for the binary outcomes.


$$P(y_{i}=1|x_{i},\beta )=\mathrm{logi}t^{-1}(\beta_{0j}+\beta_{1j}x_{i})$$

where $\beta_{1j}$ corresponds to the log odds ratio of observing the alternate allele for neighboring variant *j* given we observe the alternate allele for the test variant. A standard frequentist approach may run into issues because it is common to see some SNPs in perfect LD or near perfect LD, this creates complete or quasi-complete separation—or rare variants lead to sparsity which can also results in non-identifiability of the model.

Taking likelihood given the data *D*, L(D|β) and log likelihood l(β) as the standard Bernoulli log likelihood for logistic regression, $\log\{L(D|\beta )\}=l(\beta )=\sum [y_{i}\log(\pi_{i})+(1-y_{i})\log\;(1-\pi_{i})]$ with $\pi_{i}=\frac{\exp(X_{i}\beta )}{1+\;\exp(X_{i}\beta )}$, the penalized log likelihood can be written in the form:


(1) $$p(y_{i}|x_{i},\beta )=l(\beta )+\frac{m}{2}\beta -m\log(1+e^{\beta }).$$

It can be easily seen that at m=0 is equivalent to the maximum likelihood estimate (MLE)—further at m=1 includes Jeffrey's prior in the one parameter model, which was used in this setting based on the recommendations of Greenland and Mansournia (2015). This penalty is proportional to the posterior distribution with logF(m,m) priors, meaning estimating the function of the penalized log likelihood is equivalent to finding the posterior mode. We implemented a Bayesian logistic regression model using logF priors which enabled us to utilize established data augmentation techniques to efficiently estimate posterior coefficients (Greenland 2003, 2007; Greenland and Mansournia 2015).

To test for association between a test SNP and neighboring variant, we use the ABF. The Bayes factor (Kass and Raftery 1995) has been used in a variety of settings including in extensive use GWAS (Wakefield 2007, 2009; Maller et al. 2012; Chen et al. 2020a) and the ABF in this setting plugging in equation (1) is defined as:


$$\begin{array}{rl} & \log\{BF_{j}\}=\log\left\{\frac{\;p(y|M_{1,j})}{\;p(y|M_{0,j})}\right\}\approx \log\left\{\frac{L(D|{\tilde{\beta }}_{0},{\tilde{\beta }}_{1})p({\tilde{\beta }}_{0})p({\tilde{\beta }}_{1})\;}{L(D|{\tilde{\beta }}_{0})p({\tilde{\beta }}_{0})}\right\}\\ & =\left\{l({\tilde{\beta }}_{0},{\tilde{\beta }}_{1})+\frac{m}{2}{\tilde{\beta }}_{0}-m\log(1+e^{{\tilde{\beta }}_{0}})+\frac{m}{2}{\tilde{\beta }}_{1}-m\log(1+e^{{\tilde{\beta }}_{1}})\right\}\\ & \;-\left\{l({\tilde{\beta }}_{0})+\frac{m}{2}{\tilde{\beta }}_{0}-m\log(1+e^{{\tilde{\beta }}_{0}})\right\}. \end{array}$$

(2)The generalized connection between the ABF and other common statistics is further described below in the supplement (Connection to Other Statistics). This test compares the posterior of the intercept only model $M_{0,j}=\mathrm{logi}t^{-1}(\beta_{0j})$ versus the model with the neighboring variant $M_{1,j}=\mathrm{logi}t^{-1}(\beta_{0j}+\beta_{1j}x_{i})$. If *m* were to be set to zero, and the data augmentation omitted, that would be proportional to a simple likelihood ratio test, $\log\left\{\frac{L(\beta_{0},\beta_{1})}{L(\beta_{0})}\right\}$ (see Connection to Other Statistics). To get the test statistics across the entire neighboring region the product of these ABF between the test SNP and each neighboring SNP in a window of a thousand bases (five hundred bases up and downstream) were used, where the log is taken for computational ease. The final statistic is the log product of the ABF across the entire window (here 1 kb was used) then divided by the window size, where both taking the log and dividing by the window size are done for interpretability, plugging in equation (2).


(3) $$\mathrm{LD}\text{-}\mathrm{ABF}=\frac{1}{W}\log\overset{W}{\underset{\;j=1}{\prod }}\;\left[\frac{L(D|{\tilde{\beta }}_{0},{\tilde{\beta }}_{1})p({\tilde{\beta }}_{0})p({\tilde{\beta }}_{1})\;}{L(D|{\tilde{\beta }}_{0})p({\tilde{\beta }}_{0})}\right].$$

Monomorphic sites are considered to have uninformative ABF of 1, meaning regions that are denser with polymorphisms tend to have higher test statistics. Since LD-ABF is equivalent to the sum of the log of the ABF over a window the denser regions will tend have a larger sum over a window with less or no variants. The fast approximation means the computational complexity scales with the number of logistic regression steps, *S*, and the window size O(WS) while the memory scales with the sample size *N* and window size O(WN). Additionally, since SNPs outside a window do not contribute to the test—the software is set up to be parallelized over SNPs. For a detailed example walking through the calculations and data augmentation techniques for fast Bayesian estimation, see our online resources toy example at https://tris-10.github.io/LD-ABF/documentation/LD_ABF_toyExample and code is available online at https://github.com/tris-10/LD-ABF.

### Children's Hospital of Philadelphia Clinical Samples

In the clinical samples, 834 samples underwent quality control and outlier filtering, leaving 468 with SNP array data and matching whole exome sequencing, including 334 trios (Table 1). For samples to be included in the final analysis they needed to have both SNP array and whole exome sequence data. For both platforms, phasing was done using SHAPEIT2 (Choi et al. 2018; Delaneau et al. 2019) and then the cross platform samples were merged maximizing overlapping alternate allele matches. Since signals of selection can often be obscured or confounded by demographic shifts across populations, inference on each sample's ancestry was completed to facilitate within-population analysis. Using the first 10 principle components (PCs) calculated from the SNP array data, K-nearest neighbors clustering algorithm was run to group samples by their best matching 1000 Genomes Project (1KGP) (Auton et al. 2015) super-population—Africa (AFR), East Asia (EAS), Europe (EUR), South Asia (SAS), or the Americas (AMR) (supplementary fig. S1, Supplementary Material online). Sixteen outliers whose PC positions are more than six standard deviations away from the mean of any ancestral group were removed (Price et al. 2006; Galinsky et al. 2016). Such inference is expected to have limitations since the samples were not collected prospectively with ancestry or ethnicity assessments.

For the SNP array data, 832,381 SNPs common to the 3 SNP arrays were extracted. SNPs were then removed if they had genotyping call rate < 0.95, minor alleles frequency < 0.01. Individuals were removed if they had individual missing genotypes rates > 0.05. For the whole exome sequence data, within each family indels were separated from SNPs. Indels are excluded if QD < 2 or FS > 200 or ReadPosRankSum < −20. SNPs are excluded if QD < 2 or FS > 60 or MQ < 40 or MQRankSum < −12.5 or ReadPosRankSum < −8. Also within each family, genotypes variants were excluded if any individual had a variant with DP < 5 or GQ < 10. Exome data had to pass internal clinical filtering criteria including call quality by depth (QD) < 10 and Phred scaled *P*-value using Fisher's exact test (FS) > 5 with coverage on average at 80×. VCF files were then merged across families and missing genotypes were assumed to be reference. Monomorphic, multi-allelic and variants with Mendel error rates > 0.01 were removed. In some cases, edges of telomeric regions appeared to cause errors in phasing using SHAPEIT2, so the last 4 variants on chromosome 1 and chromosome 3 were also removed to finish the phasing.

Several transmission filters were incorporated leveraging family relatedness because certain variants appeared to be incorrectly called due to homologous sequence stretches. Several filtering steps were performed to remove regions exhibiting higher heterozygosity than expected based on equilibrium frequencies. Two tests were implemented to remove individuals that were excessively heterozygous. Relative to filtering out repeat masker regions entirely, this gives another way to remove potential artifacts by leveraging family data without having to remove close to 20% of variants. In settings where over 95% of families, either trios or duos, consisted of entirely heterozygous individuals those variants were filtered out. Then looking at complete trios, if both parents are heterozygous at an allele, the transmission of either homozygous variant is expected to be 25%. So, looking at each trio where both parents are heterozygous at a variant, a binomial test with a *P*-value threshold of 0.005 is constructed so the probability of success (i.e. seeing a homozygous proband) is *P* = 25% and for the number of observations, *n*, is equal to the number of families with heterozygous parents; variants that do not pass the threshold are then filtered out. Sometimes the reference allele was not the major allele, i.e. the major and minor allele were flipped, in which case if the minor allele occurred more than 95% it was removed, this is the same as a 5% MAF threshold.

Additional regional filters included removing regions that fell in the ENCODE black list regions https://github.com/Boyle-Lab/Blacklist/ (Amemiya et al. 2019) low complexity repeat regions (LCR): https://raw.githubusercontent.com/lh3/varcmp/master/scripts/LCR-hs37d5.bed.gz, removing centromeres (acen) and telomers (gvar) UC genome browser and taking http://hgdownload.cse.ucsc.edu/goldenPath/hg19/database/cytoBand.txt.gz and any remaining indels. The ENCODE blacklist represent a large number of repeat elements in the genome or more generally regions that have anomalous, unstructured, or high signal in next-generation sequencing experiments independent of cell line or experiment. Furthermore, the filtration process entailed the exclusion of segmental duplicates displaying a fraction of matching bases (fracMatch) exceeding 95%, long terminal repeats (LTRs), and repeats possessing a 100mer mappability index below 1 (obtained from https://hgdownload.cse.ucsc.edu/goldenPath/hg19/encodeDCC/wgEncodeMapability/wgEncodeCrgMapabilityAlign100mer.bigWig) via the UCSC Genome Browser. When running LD-ABF, the within-population variants were restricted to MAF > 0.05.

Similar setups across the different comparator methods were run as noted in the simulation study. B2 required additional handling. Whole-genome pairwise alignments from the UCSC Genome Browser were used to perform a comparative analysis between human and chimpanzee genomes (http://hgdownload.cse.ucsc.edu/goldenpath/hg19/vsPanTro6/hg19.panTro6.net.axt.gz). To generate input files containing relevant genetic information, the “getChrAxt.sh” script was employed to extract individual chromosomes followed by the use of the “parse_ballermix_input_v2.py” script. The output files contained information on physical positions, genetic positions, and the number of derived and total observed alleles for each variant in human chromosomal VCF files. To ensure a consistent sample size, positions with smaller sample sizes were removed from the input files. The generation of a site frequency spectrum file was achieved by concatenating all input files and running BalLeRMix_v2.py with the “–getSpect” flag. Finally, B2 statistics for each variant on every chromosome were estimated using the chromosomal input files and the site frequency spectrum.

Top 100 peaks for each population are reported online. To be conservative in avoiding double counting peaks within long extended LD, the analysis was first performed using neighborhoods of 1 Mb around the highest local scores. A follow-up analysis was then performed using 100 kb neighborhoods to detect peaks at a finer granularity (supplementary Online Data, Supplementary Material online). The gene families are defined using Human Genome Organization (HUGO) gene naming HUGO Gene Nomenclature Committee (HGNC) (https://www.genenames.org/).

### 17th IHIW and IMGT

Samples were taken from the 17th IHIW, using reported high-resolution allele frequencies characterized by next-generation sequencing in unrelated populations (i.e. no known familiar relationship between samples) (17th IHIW Table 1). This dataset consists of over 3,500 samples, each providing 2 alleles per HLA gene typed at 4 field resolution and represents a diverse set of world populations: European Americans, African Americans, US Hispanics, Spanish, Mexican, Italian, Greek, Asian Pacific Islanders, Thai, Indian, Arab, and Europeans (taken from the 17th IHIW Table 1). Since the samples reported include allele frequencies using classic HLA nomenclature, to perform analysis the data required matching on consensus sequencing then lifting over to reference. The observed alleles in 17th IHIW were matched with their established sequences, as described in the interational ImMunoGeneTics (IMGT) HLA database version 3.25.0, which is the version that most directly corresponds to the 17th IHIW and lifted over to Hg19. Indels, short tandem repeats (STRs), and missing variants were ignored for this analysis. In the 17th IHIW dataset, alleles ending with “SG” in their name refer to STR allele ambiguity groups; when encountering such alleles, we have removed the suffix to enable matching with a corresponding and representative IMGT allele. If an allele reported in 17th IHIW did not match up with a fully sequenced HLA allele in IMGT 3.25.0 then it is omitted. This typically only occurred with rare alleles, where all but DPB1*01:01:01 had allele frequencies below 5%. Low frequency alleles are expected to have less of an impact on the analysis than higher frequency alleles since LD is typically less strong for rare alleles. Genes without genomic alignment file for IMGT 3.25.0 were also omitted. Alleles were 4 field typed except where amplicons do not extend the full length of the gene where ambiguities are noted by the 17th IHIW (http://17ihiw.org/wp-content/uploads/2018/10/Readme-Unrelated-HLA-allele-and-haplotypes-FQ-tables_072318.pdf).

### Pangenome Samples

Freeze 1 version 2 assembly data was downloaded from the Human Pangenome Reference Consortium (HPRC) repository. The assemblies were aligned to hg38 chromosome 6 using minimap2 (v2.21) in asm20 mode. All contigs with a total alignment length exceeding 500 K were retained for variant calling. Filtered contigs were processed with Dipcall (v0.3), adjusted to use modified minimap2 alignment settings accounting for the high variability in the MHC region (*-x asm20 -m 10000 -z 10000,50 -r 50000 –end-bonus=100 –secondary=no –cs -O 5,56 -E 4,1 -B 5*). The reference sequence was hg38 chr6 masked between the HLA-DRA and HLA-DRB1 regions (32,494,000 to 32,565,000). The validity of the alignment settings was checked by extracting the contig sequences across each of the canonical HLA genes and typing with GenDx (v2.20.2) in PacBio Consensus mode. The resulting variant calls were restricted to SNPs between 29,657,092 to 33,323,016 and merged into a single VCF using vcftools (v0.1.16). Public Dipcall variant calls across the entire genome were downloaded from the HPRC repository. Calls were restricted to SNPs outside of the MHC region and merged into a single VCF. The two sets of variant calls were combined, and non-variant positions were set to homozygous reference if the position was within a region reported as callable by Dipcall. The same filters for encode black list regions, LCR, centromere/telomere, and indels as were used on the clinical samples, just with LiftedOver to hg38. Samples were restricted to the African individuals and the two PC outliers were removed (supplementary fig. 14, Supplementary Material online). The largest population consists of just 23 African samples (after removing two PC outliers) and other populations were too small to perform statistical inference for this study. A scan was run filtering on segmental duplications and another without.

## Supplementary Material

Supplementary material is available at *Genome Biology and Evolution* online.

## Supplementary Material

evae009_Supplementary_Data

## Data Availability

The IHIW samples allele frequencies were downloaded from the IHIW data website (http://17ihiw.org/17th-ihiw-ngs-hla-data/) and corresponding reference sequence was downloaded and matched to IMGT (https://www.ebi.ac.uk/ipd/imgt/hla/). Pangenome assemblies were downloaded from their website (https://s3-us-west-2.amazonaws.com/human-pangenomics/index.html?prefix=working/HPRC/HG01361/assemblies/). In addition to the code, data files can be downloaded from online data (https://github.com/tris-10/LD-ABF Readme.md section Download LD-ABF supplemental files): (i) CHOP Trios: Genome Wide LD-ABF test statistics and peaks detailed for all included populations in Hg19, (ii) All 17th IHIW: HLA LD-ABF test statistics for all included populations, tab delimited sequence data generated from 17th IHIW and IMGT 3.25 with lifted over alignments to Hg19 performed. Plots across all genes for all included populations, (iii) Pangenome Freeze 1 African samples: LD-ABF test statistics and variant calling vcfs in Hg38 for samples. Individual gene information was found in the NCBI gene database https://www.ncbi.nlm.nih.gov/gene/ and also through GeneCards www.genecards.org. HUGO Gene Name Committee was downloaded from https://www.genenames.org/data/genegroup/#!/group/589. In addition to the code, data files (Hayeck et al. 2024) can be downloaded from online data (https://github.com/tris-10/LD-ABF Readme.md section Download LD-ABF supplemental files).
