# Supplementary material for: The Impact of Patterns in Linkage Disequilibrium and Sequencing Quality on the Imprint of Balancing Selection
Source: Genome Biol Evol. 2024 Feb 1;16(2):evae009. doi: 10.1093/gbe/evae009 (PMC10853003; doi:10.1093/gbe/evae009)
Supplement: evae009_Supplementary_Data [file evae009_supplementary_data.docx]

Supplement: The Impact of Patterns in Linkage Disequilibrium and Sequencing Quality on the Imprint of Balancing Selection

Tristan J. Hayeck^1,2*^, Yang Li^1,2^, Timothy L. Mosbruger^1^, Jonathan P Bradfield,^3^ Adam G. Gleason^1^, George Damianos^1^, Grace Tzun-Wen Shaw^1^, Jamie L. Duke^1^, Laura K. Conlin, Tychele N. Turner^4^, Marcelo A. Fernández-Viña^5,6^, Mahdi Sarmady^1,2^, Dimitri S. Monos^1,2^

**^1^**Division of Genomic Diagnostics, Department of Pathology and Laboratory Medicine, Children’s Hospital of Philadelphia, Philadelphia, Pennsylvania, USA.

^2^Department of Pathology and Laboratory Medicine, Perelman School of Medicine, University of Pennsylvania, Philadelphia, Pennsylvania, USA.

^3^Quantinuum Research LLC, Philadelphia, PA, USA

^4^Department of Genetics, Washington University School of Medicine, St. Louis, MO 63110, USA

^5^Department of Pathology, Stanford University School of Medicine, Palo Alto, CA, USA

^6^Histocompatibility and Immunogenetics Laboratory, Stanford Blood Center, Palo Alto CA, USA

*Corresponding author

Table of Contents

[Online Methods 2](#_Toc157767392)

[Connection to Other Statistics 2](#_Toc157767393)

[Simulation of Balancing Selection from Evolutionary Simulations 3](#_Toc157767394)

[Bibliography 5](#_Toc157767395)

[Supplementary Figures: 6](#_Toc157767396)

[20](#_Toc157767397)

[Supplementary Tables 21](#_Toc157767398)

# Online Methods

## **Connection to Other Statistics**

The connection between the Bayes factor (BF), Bayesian Information Criteria (BIC), the likelihood ratio test, and the approximate Bayes factor (ABF) used will be described here. Consider some set of data, $D$, with a set of parameters $\boldsymbol{\theta}$ for some model $M_{j}$ with probability of that observed model $\pi_{j}$. Then the posterior probability of the model $M_{j}$ is then defined as

$${P(M}_{j}\left| D \right)=\frac{p\left( D | M_{j} \right)\pi_{j}}{p\left( D \right)}$$

and

$$p\left( D | M_{j} \right)=\int p\left( D | \theta_{j} \right)p\left( \theta_{j} \right)d\theta_{j}$$

To compare two different models, $M_{1}$ and $M_{0}$, the ratio of the posteriors can be taken:

$$\frac{{P(M}_{1}\left| D \right)}{{P(M}_{0}\left| D \right)}=\frac{p\left( D | M_{1} \right)\pi_{1}}{p\left( D | M_{0} \right)\pi_{0}}$$

The BF is then defined as the ratio of the probability of the observed data given model one versus model two or integral over the product of the likelihood and the priors:

$$BF=\frac{p\left( D | M_{1} \right)}{p\left( D | M_{0} \right)}=\frac{\int\mathcal{L}\left( D | \theta_{1} \right)p\left( \theta_{1} \right)d\theta_{1}}{\int\mathcal{L}\left( D | \theta_{0} \right)p\left( \theta_{0} \right)d\theta_{0}}$$

Next, it’s helpful to connect the BF to the BIC. Raftery shows the derivation of the BIC, under certain regularity conditions, which is the Taylor series approximation of $g\left( \theta_{j} \right)=\log\left\{ \mathcal{L}\left( D | \theta_{j} \right)p\left( \theta_{j} \right) \right\}$ about $\tilde{\theta}_{j}$ the value that maximizes $g\left( \theta_{j} \right)$, the posterior mode (Kass and Raftery 1995; Raftery 1995).

$$g\left( \theta_{j} \right)\approx g\left( \tilde{\theta}_{j} \right)+\frac{1}{2}\left( {\theta_{j}-\tilde{\theta}}_{j} \right)g^{''}\left( \tilde{\theta}_{j} \right)\left( {\theta_{j}-\tilde{\theta}}_{j} \right)+o(\left\| {\theta_{j}-\tilde{\theta}}_{j} \right\|^{2})$$

So then if this is plugged back into $p\left( D | M_{j} \right)$:

$$p\left( D | M_{j} \right)=\int exp[g\left( \theta_{j} \right)]d\theta_{j}\approx\exp\left[ g\left( \tilde{\theta}_{j} \right) \right]*\int\exp\left[ \frac{1}{2}\left( {\theta_{j}-\tilde{\theta}}_{j} \right)g^{''}\left( \tilde{\theta}_{j} \right)\left( {\theta_{j}-\tilde{\theta}}_{j} \right) \right]d\theta_{j}$$

The integrand is proportional to a multivariate normal (MVN). Since the integral of a probability density function is by definition one, Raftery was then able to solve the integral for the proportional terms. Under certain regularity conditions in large sample sizes the posterior mode is approximately equal to the maximum likelihood estimate $\tilde{\theta}_{j}\approx\hat{\theta}_{j}$ and the expected value for the information matrix for one observation $\left| I\left( \theta_{j} \right) \right|\approx-E\left[ \frac{\partial^{2}\log\left\{ p\left( D | \theta_{j} \right) \right\}}{\partial\theta_{j}\partial\theta_{j}^{T}} \right]\left. \right|_{\theta_{j}=\hat{\theta}_{j}}=n\left| I\left( \hat{\theta_{j}} \right) \right|$. So, going back and taking the $-2\log\{p\left( D | M_{j} \right)\}$ and plugging in this approximation:

$$-2\log\left\{ p\left( D | M_{j} \right) \right\}\approx log\left\{ \exp\left[ g\left( \tilde{\theta}_{j} \right) \right]\frac{2\pi^{\frac{d_{j}}{2}}}{n^{\frac{d_{j}}{2}}\left| I\left( \hat{\theta_{j}} \right) \right|^{\frac{1}{2}}} \right\}$$

$$=-2*log \left\{ \mathcal{L}\left( D | \tilde{\theta}_{j} \right) \right\}-2 log \left\{ p\left( \tilde{\theta}_{j} \right) \right\}-d_{j}\log\left\{ 2\pi\right\}+d_{j}\log\left\{ n \right\}+log\left\{ I\left( \hat{\theta_{j}} \right) \right\}$$

Where the BIC for $M_{j}$ ignores the terms of lower order that will contribute less:

$$BIC\left( M_{j} \right)=\log\left\{ \mathcal{L}\left( D | \theta_{j} \right) \right\}-\frac{d_{j}}{2}\log\left\{ n \right\}$$

So, the difference between two models is an approximation of the BF:

$$\log\left\{ BF \right\}=\log\left\{ \frac{p\left( D | M_{1} \right)}{p\left( D | M_{0} \right)} \right\}\approx BIC\left( M_{1} \right)-BIC\left( M_{0} \right)+O(n^{-\frac{1}{2}})$$

The LD-ABF test statistic similarly ignores the information matrix term to give:

$$\log\left\{ BF \right\}=\log\left\{ \frac{p\left( D | M_{1} \right)}{p\left( D | M_{0} \right)} \right\}\approx\log\left\{ \frac{\mathcal{L}\left( D | \tilde{\theta}_{1} \right)p\left( \tilde{\theta}_{1} \right)}{\mathcal{L}\left( D | \tilde{\theta}_{0} \right)p\left( \tilde{\theta}_{0} \right)} \right\}=\log\left\{ \mathcal{L}\left( D | \tilde{\theta}_{1} \right) \right\}+2 log \left\{ p\left( \tilde{\theta}_{1} \right) \right\}-\log\left\{ \mathcal{L}\left( D | \tilde{\theta}_{0} \right) \right\}-2 log \left\{ p\left( \tilde{\theta}_{0} \right) \right\}$$

We chose to ignore the $\frac{d_{j}}{2}\log\left\{ n \right\}$ which will be approximately constant for a study with constant sample sizes (subject to missing data at variants) and include the prior term$\log\left\{ p\left( \tilde{\theta}_{j} \right) \right\}$. Similarly, the likelihood ratio test (LRT) is $-2*log \left\{ \frac{\mathcal{L}\left( D | \tilde{\theta}_{1} \right)}{\mathcal{L}\left( D | \tilde{\theta}_{0} \right)} \right\}$, which would be under a setting of completely uninformative priors. The LRT is asymptotically chi-squared distributed so, the ABF will also be approximately chi-square distributed asymptotically as well. LD-ABF scales with samples size, so as a rule of thumb taking the LD-ABF divided by the samples size and testing if it is above 0.005 appears a reasonable threshold for detecting signatures of selection (Supplemental Table 11); although, we caution to interpret in similar settings, within population, with large samples sizes, and in settings where model assumptions hold. Generally, it is preferable to characterize relative signal as shown in the results looking at different peaks.

## **Simulation of Balancing Selection from Evolutionary Simulations**

To test the effectiveness of LD-ABF in detecting balancing selection, a series of forward time simulations were run using SLiM 3.0(Haller and Messer 2019a, 2019b) and the statistics ability to detect variants under selection, versus neutral drift, was compared with other test statistics (see results for overview). For consistency, all methods used a window size of 1Kb. For the different methods: To calculate Tajima’s D scikit-allel was used (Alistair Miles, pyup.io bot, Murillo R., Peter Ralph, Nick Harding, Rahul Pisupati, Summer Rae 2021), β and β_2,std_ BetaScan was run with <https://github.com/ksiewert/BetaScan>, B2 using https://github.com/bioXiaoheng/BallerMixPlus, while D_ng_ and HKA were explicitly calculated by our software. Most methods could be run using default settings and limited additional parsing by our scripts. B2 required additional special processing. Genome alignment files in ATX format were generated by scanning the minor allele frequencies of each position in the human and chimp SLiM simulation files. The genome sequence was set to the alternate allele if the minor allele frequency was greater than 75%, otherwise the reference allele was retained. BalLeRMix input files were generated using the helper script ‘parse_ballermix_input.py’ provided by BalLerMix+, specifying the custom ATX file, the SLIM output file, and a recombination rate of 2.5e-8. BalLerRMix_v2.3.py was run on the full set of neutral drift simulation files using the ‘--getSpect’ flag to generate a site frequency spectrum file. Finally, BalLeRMix_v2.3.py was used to generate B2 statistics on each simulation using a recombination rate of 2.5e8, physical variant positions, and the spectrum file generated in the previous step (--rec 2.5e-8 --physPos --fixSize -w 1000).

In the new supplementary set of simulations, looking at more recent balancing selection (Supplemental Figure 2 and Supplemental Table 1) LD-ABF appears to outperform the D_ng_ more consistently showing up to 4.1% improvement in AUC and 7.0% improvement in F_1_ at an equilibrium frequency of 50%. The Tajima’s D outperforms all though at an allele frequency of 50% with AUC = 71.7%; however, is again somewhat volatile in its ability to detect selection showing only 54.5% AUC, almost a coin flip, at an equilibrium frequency of 75%. All methods have a harder time picking up on selection signal when the balancing selection variant arises more recently, this is to be expected that over a shorter time scale the ability to detect selection is more difficult since it has not had time to propagate through the population. As sample sizes increase or the relative selection coefficients are stronger it is expected all methods would also perform better. Generally, the trend appears to show LD-ABF better predicts recent variants under selection.

## **Bibliography**

Alistair Miles, pyup.io bot, Murillo R., Peter Ralph, Nick Harding, Rahul Pisupati, Summer Rae, & Tim Millar. 2021. “No Title.” *Zenodo*. cggh/scikit-allel: v1.3.3 (v1.3.3).

Bitarello, Barbara D. et al. 2018. “Signatures of Long-Term Balancing Selection in Human Genomes.” *Genome Biology and Evolution* 10(3): 939–55.

Chen, William S. et al. 2020. “Autoantibody Landscape in Patients with Advanced Prostate Cancer.” *Clinical Cancer Research* 26(23): 6204–14.

Cheng, Xiaoheng, and Michael Degiorgio. 2019. “Detection of Shared Balancing Selection in the Absence of Trans-Species Polymorphism.” *Molecular Biology and Evolution* 36(1): 177–99.

Haller, Benjamin C., and Philipp W. Messer. 2019a. “Evolutionary Modeling in SLiM 3 for Beginners.” *Molecular Biology and Evolution* 36(5): 1101–9.

———. 2019b. “SLiM 3: Forward Genetic Simulations Beyond the Wright-Fisher Model.” *Molecular Biology and Evolution* 36(3): 632–37.

Kass, Robert E., and Adrian E. Raftery. 1995. “Bayes Factors.” *Journal of the American Statistical Association* 90(430): 773–95.

Palamara, Pier Francesco, Jonathan Terhorst, Yun S. Song, and Alkes L. Price. 2018. “High-Throughput Inference of Pairwise Coalescence Times Identifies Signals of Selection and Enriched Disease Heritability.” *Nature Genetics* 50(9): 1311–17. http://dx.doi.org/10.1038/s41588-018-0177-x.

Raftery, Adrian E. 1995. “Bayesian Model Selection in Social Research.” *Sociological Methodology* 25(1995): 111. https://www.jstor.org/stable/271063?origin=crossref.

Siewert, Katherine M., and Benjamin F. Voight. 2017. “Detecting Long-Term Balancing Selection Using Allele Frequency Correlation.” *Molecular Biology and Evolution* 34(11): 2996–3005. https://academic.oup.com/mbe/article/34/11/2996/3988103.

Siewert, Katherine M, and Benjamin F Voight. 2020. “BetaScan2: Standardized Statistics to Detect Balancing Selection Utilizing Substitution Data.” *Genome Biology and Evolution*: 1–20.

Tennessen, Jacob A, and Manoj T Duraisingh. 2021. “Three Signatures of Adaptive Polymorphism Exemplified by Malaria-Associated Genes” ed. Rebekah Rogers. *Molecular Biology and Evolution* 38(4): 1356–71. https://academic.oup.com/mbe/article/38/4/1356/5981112.

# Supplementary Figures:

| 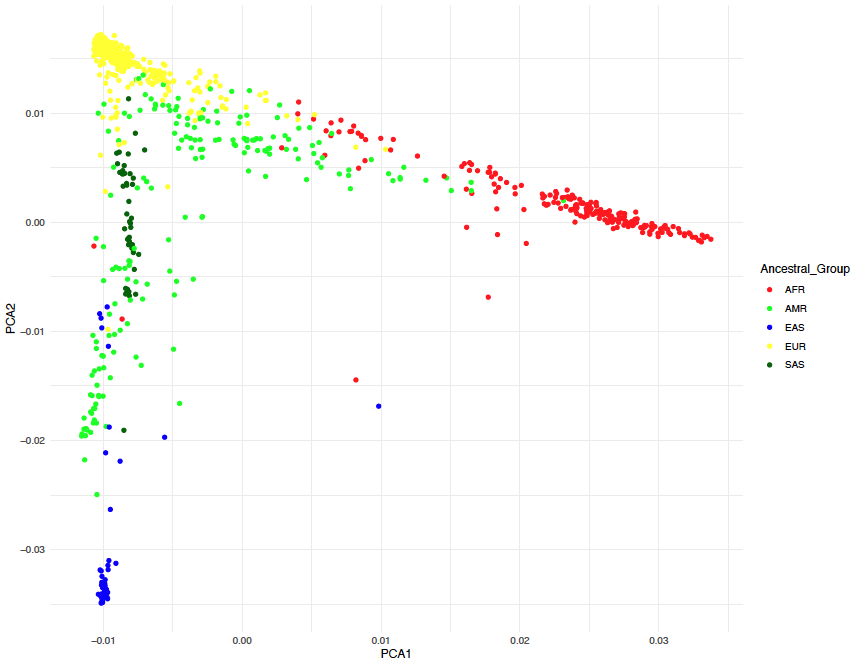 |
| --- |
| 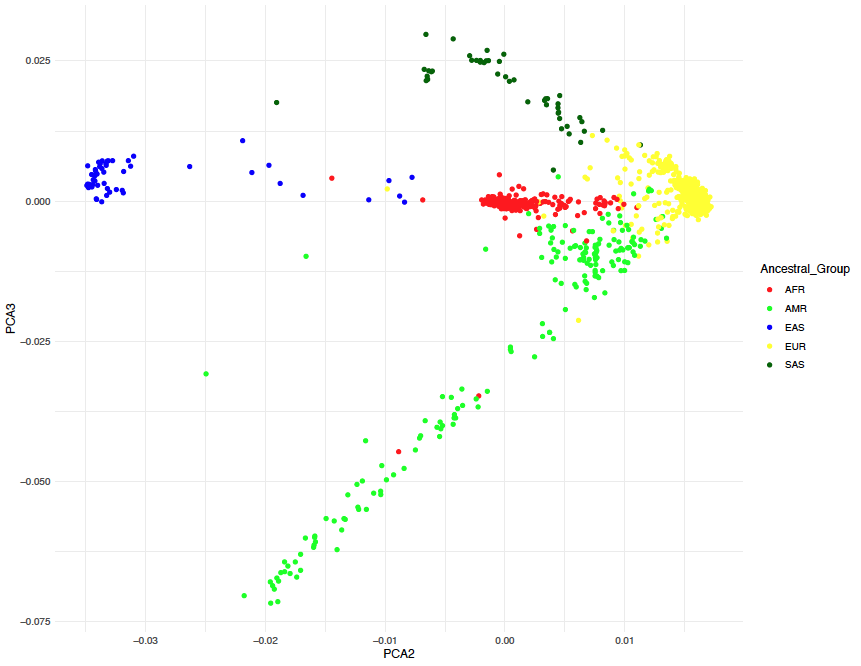 |
| 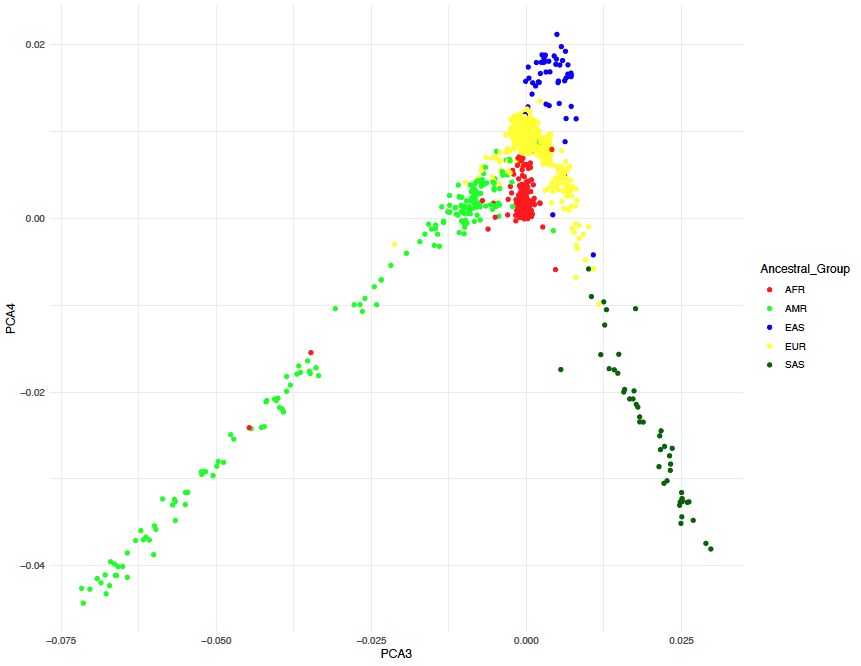 |
| **Supplemental Figure 1 Principal component analysis used for ancestry inference for clinical.** Samples are color coded based on closest Thousand Genomes super population they are clustered in where this is the PC analysis before the outliers were removed. |

| 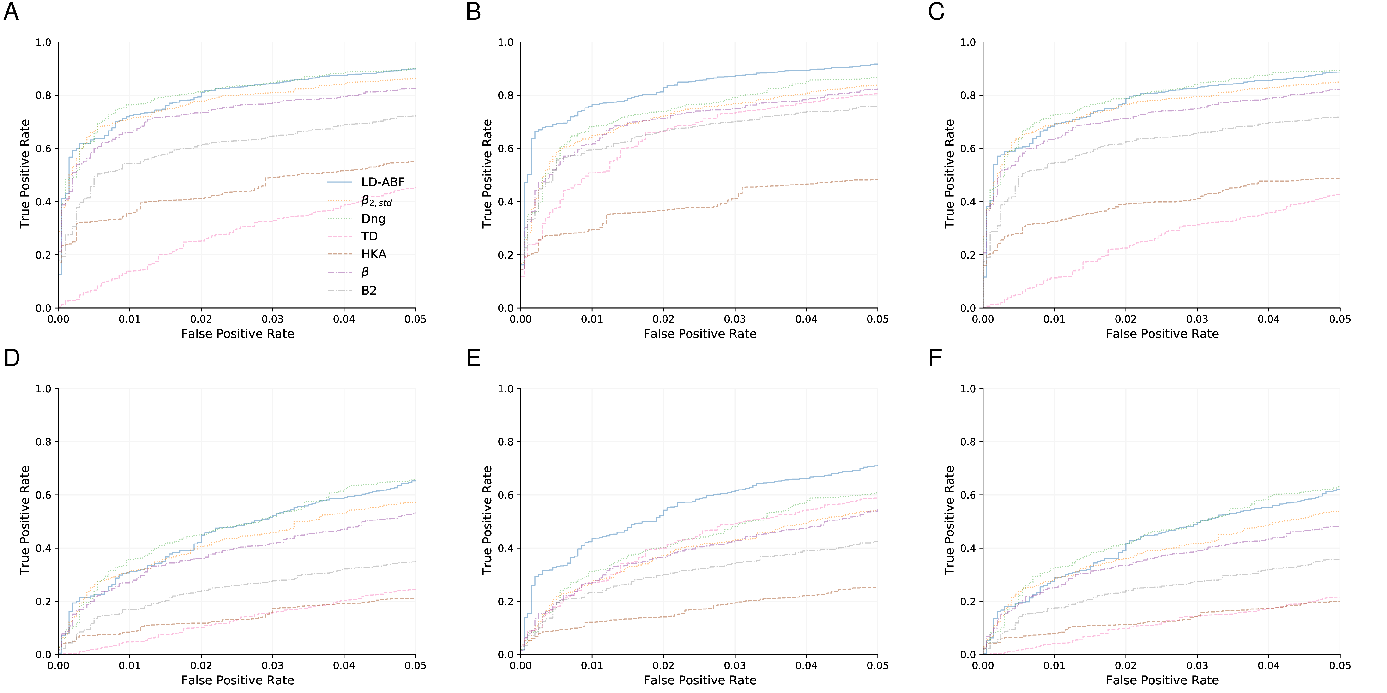 |
| --- |
| **Supplemental Figure 2 Evolutionary simulation comparison of methods’ ability to detect balancing alleles relative to neutral alleles in different scenarios of equilibrium allele frequency and time when mutation is introduced.** The top row (A, B, C) corresponds to older mutations (250,000 generations before completion) versus the second row (D, E, F) corresponding to younger mutations (100,000 generations back) replicating similar setups described by Siewert and Voight (Siewert and Voight 2017, 2020). The left column corresponds to a derived allele frequency of 0.25 (A and D), then 0.5 for the middle (B and E), and 0.75 (C and F) for the right column. True positives are taken from the 2,000 balancing selection simulations for each plot and a random common neutral mutation (with MAF>5%) is used from each of the 2,000 neutral simulations to compare as a false positive. |

| 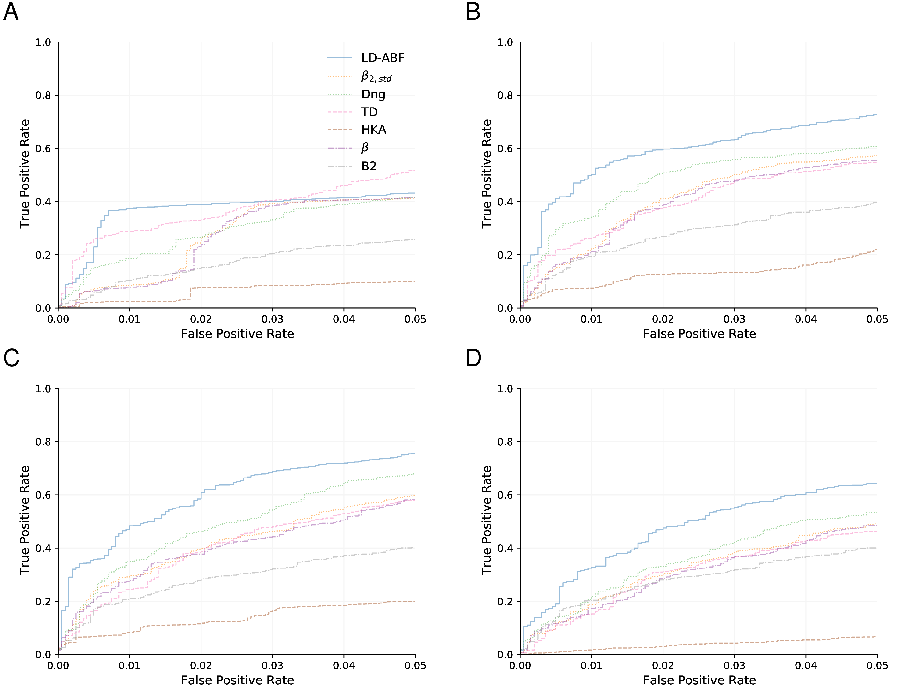 |
| --- |
| **Supplemental Figure 3 Evolutionary simulation comparison of methods’ ability to detect balancing alleles relative to neutral alleles at different window sizes.** All test statistics were calculated using window size of A) 100, B) 500, C) 1,000 or D) 5,000 base pairs and corresponding simulations with younger mutations (100,000 generations back) at an equilibrium frequency of 50 and a selection coefficient of 10^-4^. True positives are taken from the 2,000 balancing selection simulations for each plot and a random common neutral mutation (with MAF>5%) is used from each of the 2,000 neutral simulations to compare as a false positive. This corresponds to the same sets of simulations found in Supplemental Table 2. |

| 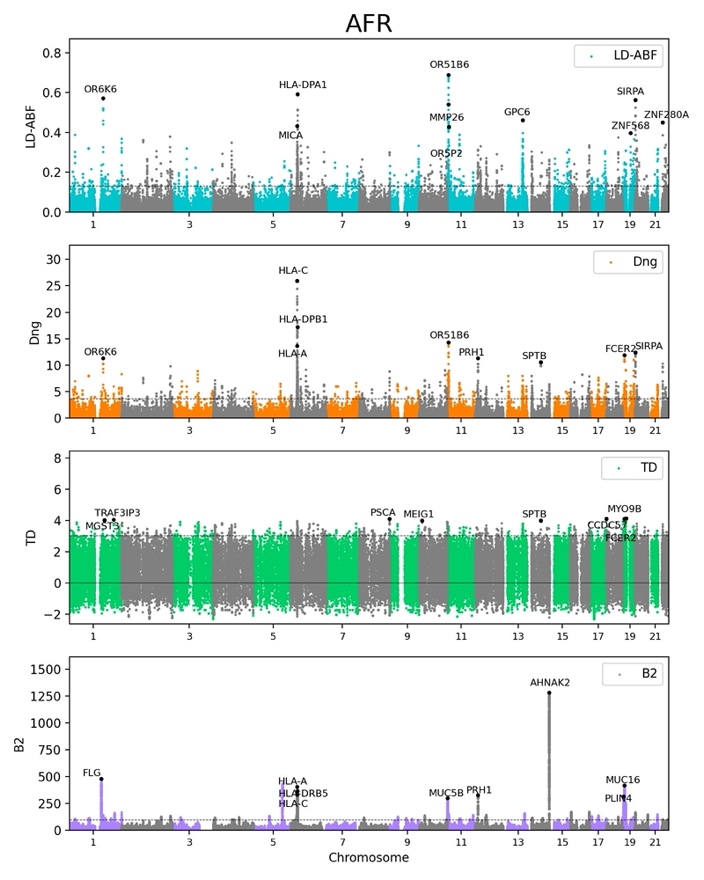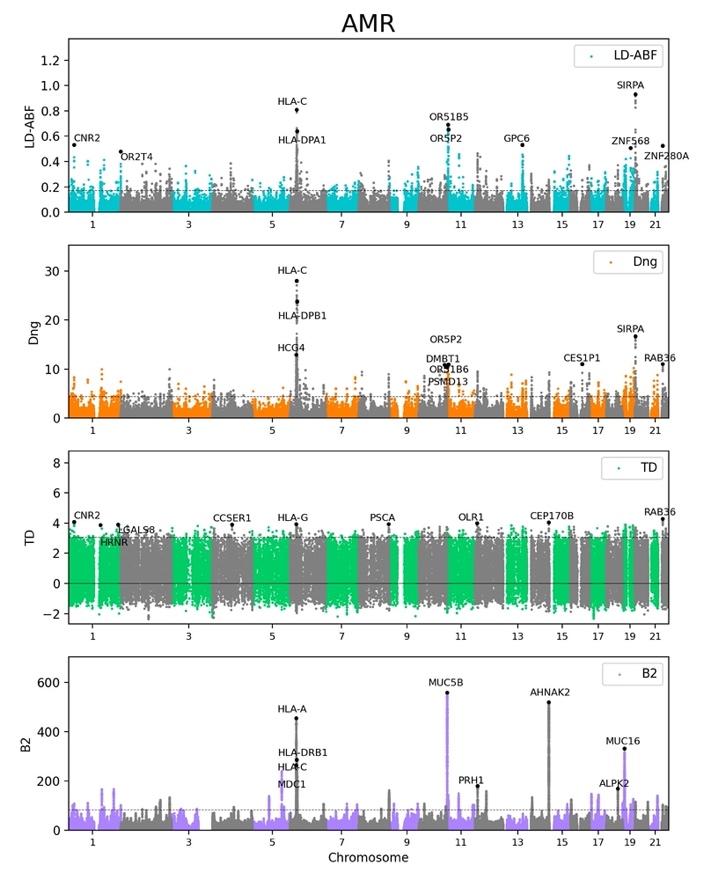  **B**  **A** |
| --- |
| 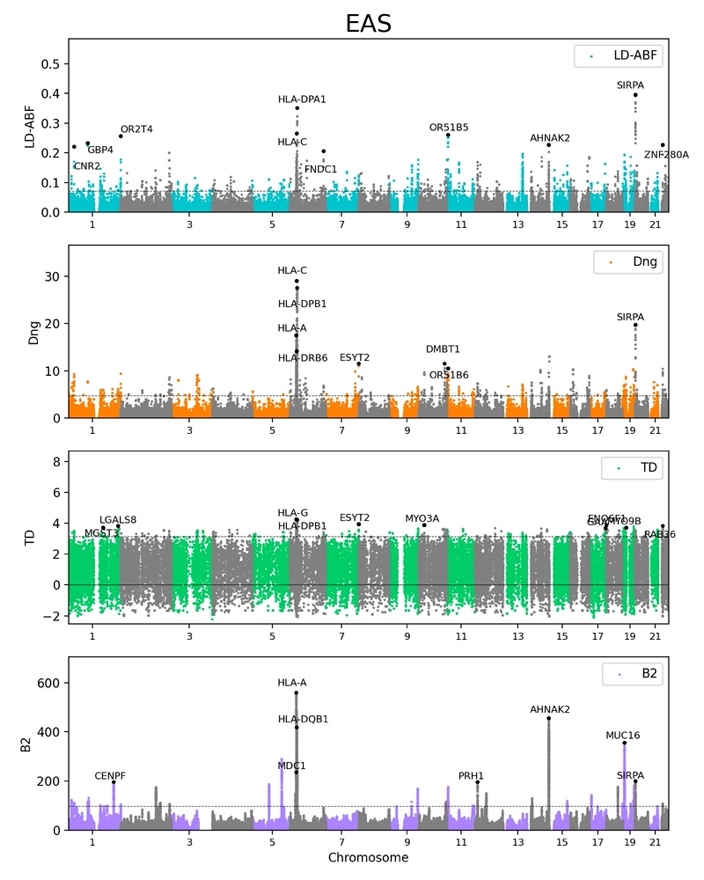 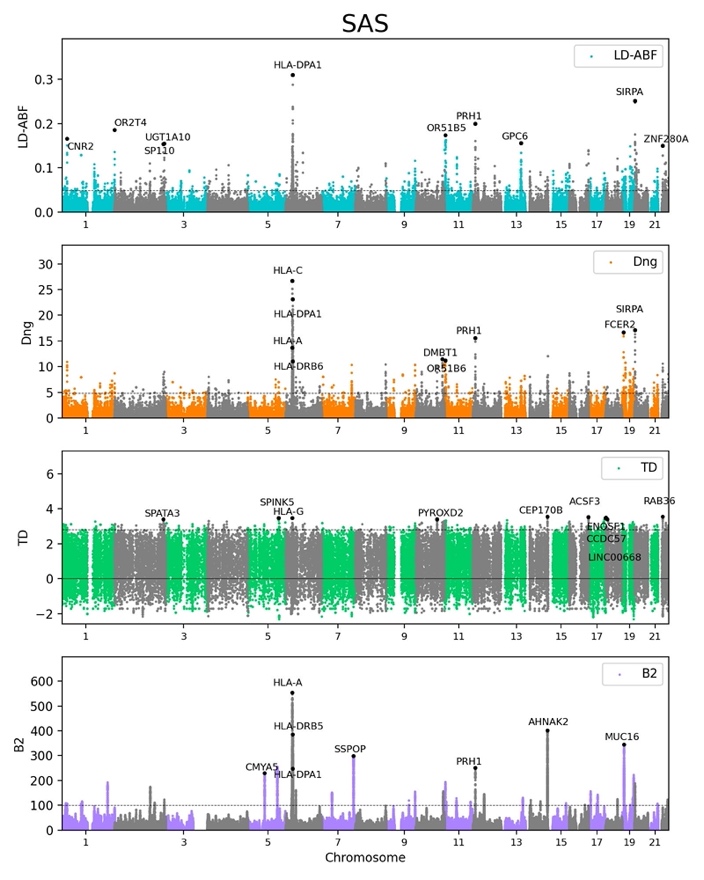  **C**  **D** |
| **Supplemental Figure 4 Genome wide scan for balancing selection looking across three different clinical populations. A) AFR B.) AMR C.) EAS and D.) SAS.**  The top ten peaks are denoted with a dot and gene label, when it falls within a gene, for each statistic are plotted along with the line denoting the top 99.9% percentile. The relative magnitude of the LD-ABF and B2 signals reflect the sample size of the population as any standard test statistic would. |

| 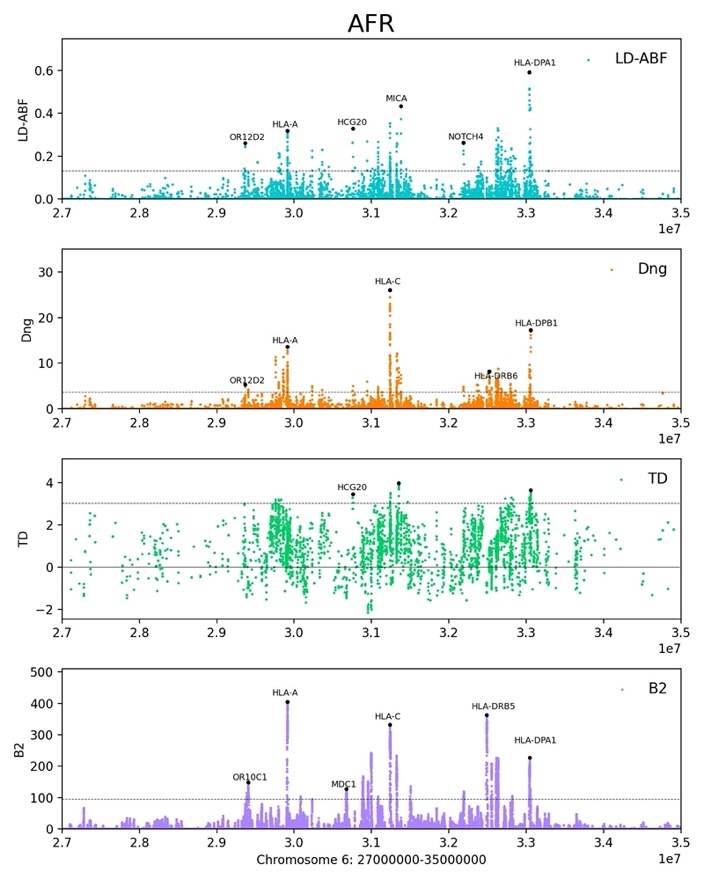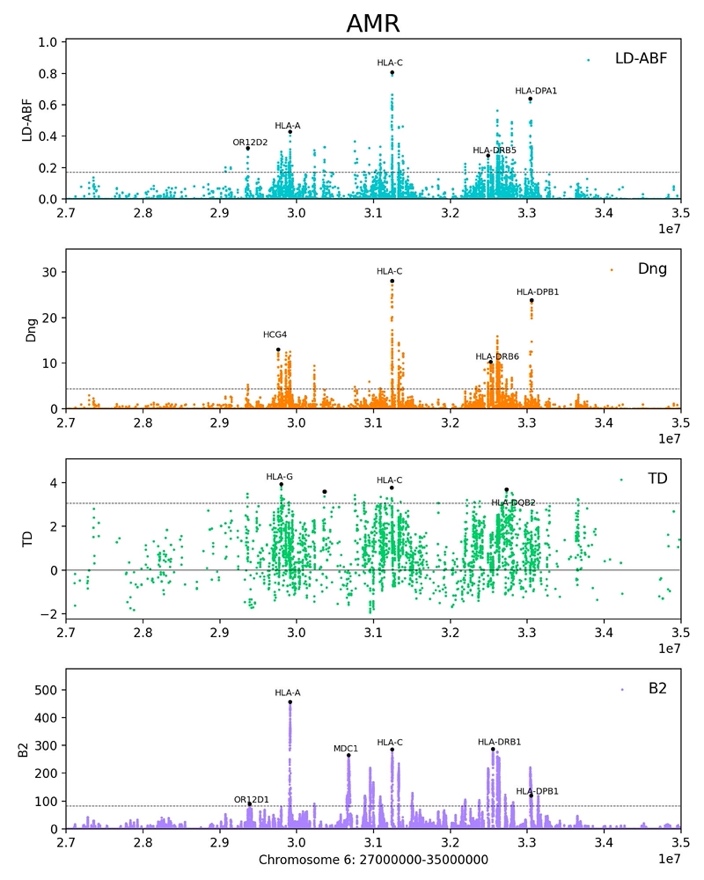  **B**  **A**  **D** |
| --- |
| 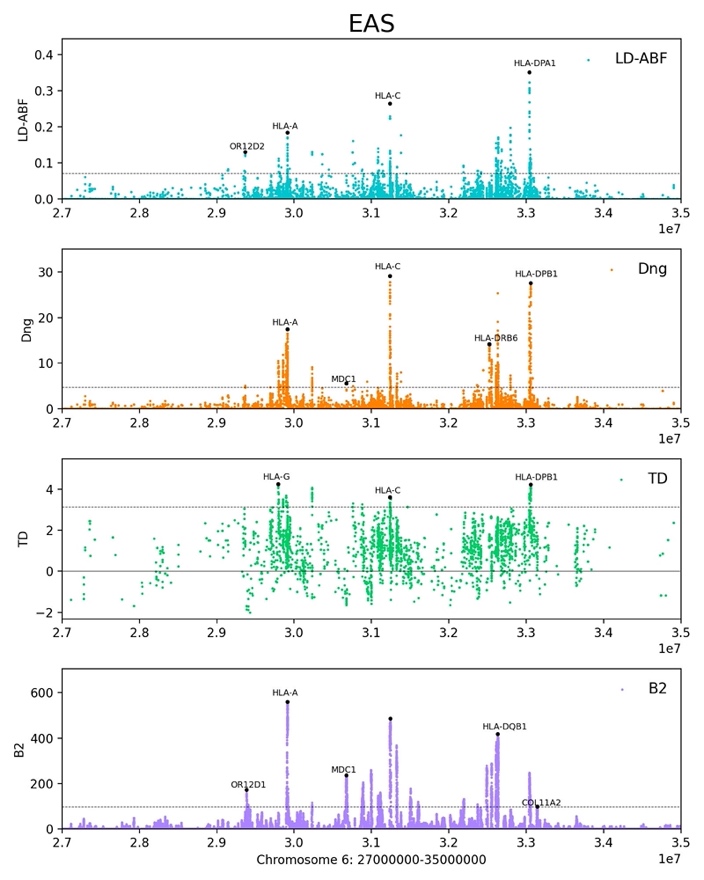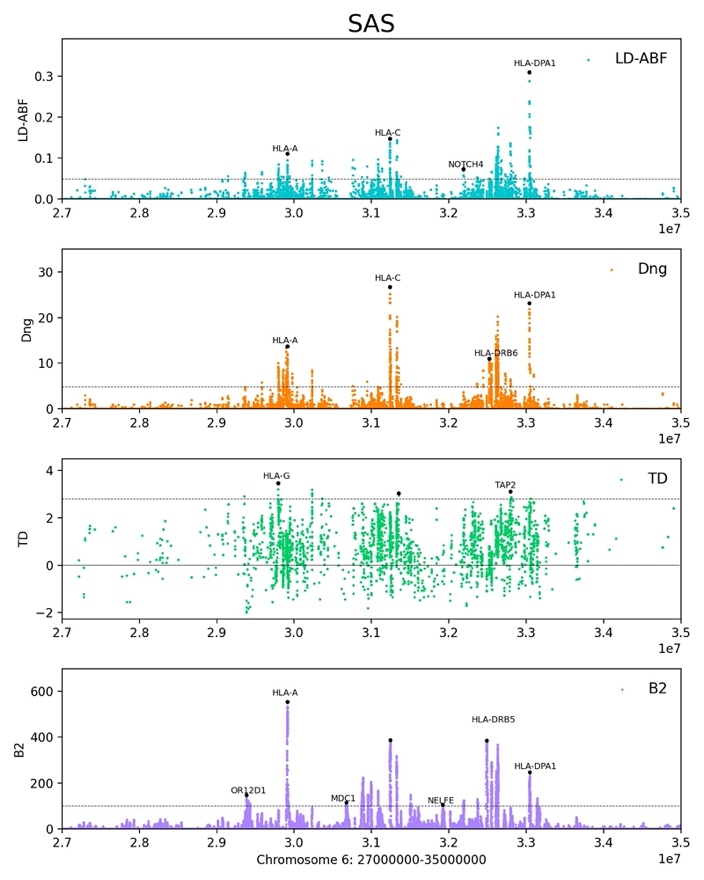  **C** |
| **Supplemental Figure 5 Detailed look at balancing selection chromosome 6 and the MHC in clinical samples.** Looking at the A) AFR, B) AMR, C) EAS, and D) SAS LD-ABF scan across the clinical samples both over the MHC (A, C, E, G) and zooming in around the MHC region (B, D, E, F). The top ten peaks are denoted with a dot and gene label, when it falls within a gene, for each statistic are plotted along with the line denoting the top 99.9% percentile. |

| 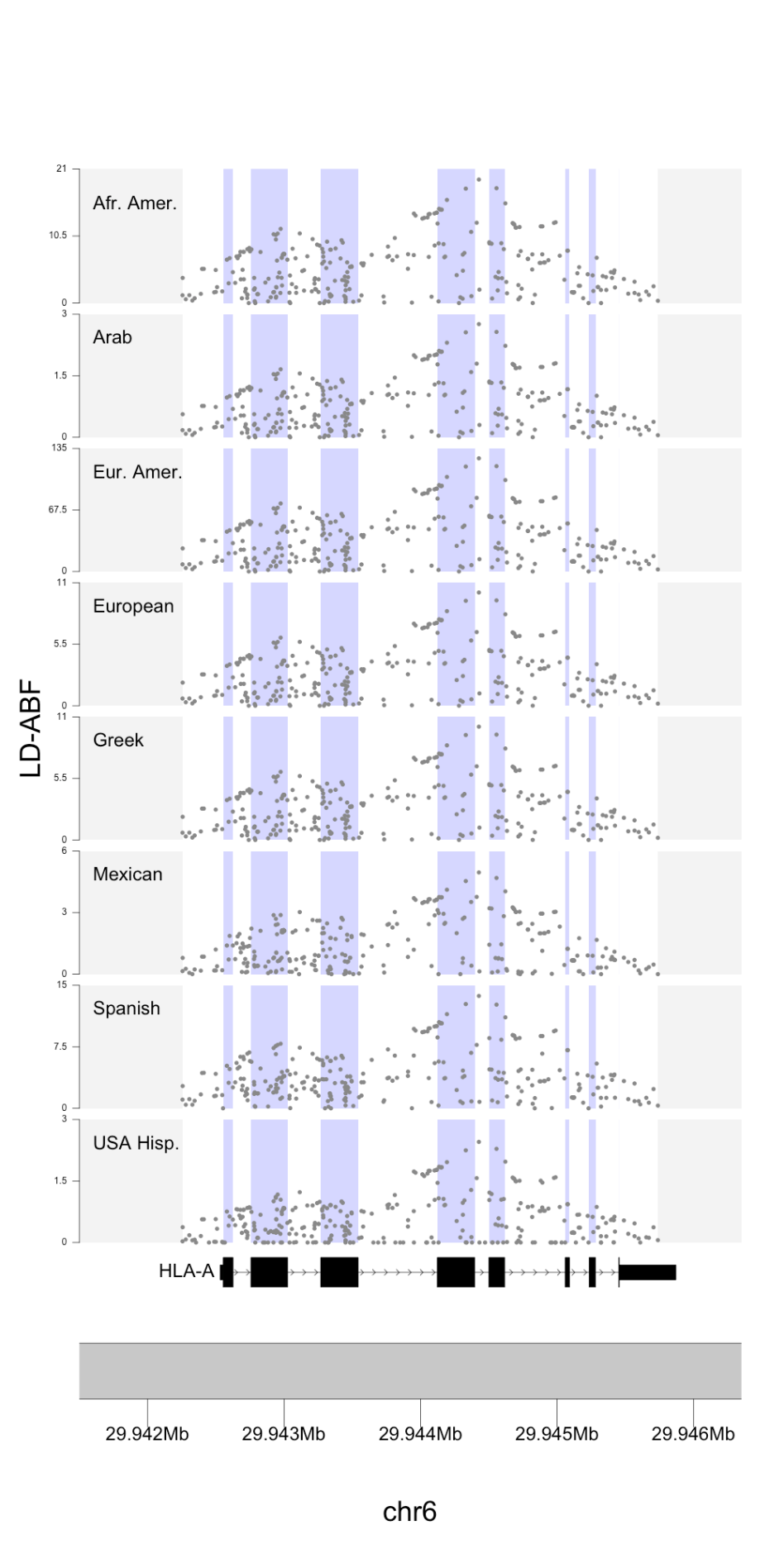 |
| --- |
| **Supplemental Figure 6 Comparison of LD-ABF across 17^th^ IHIW populations for HLA-A** |

| 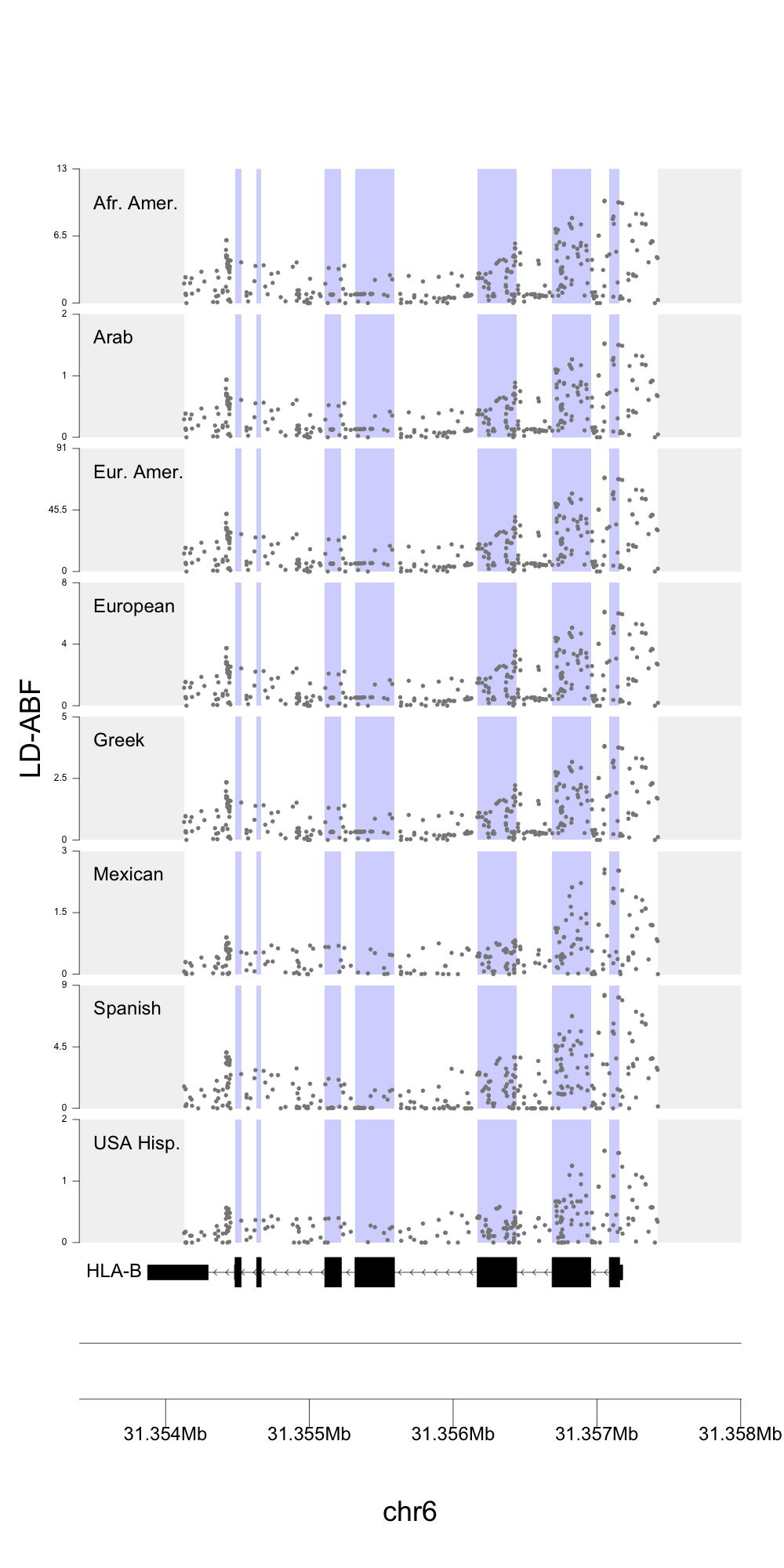 |
| --- |
| **Supplemental Figure 7 Comparison of LD-ABF across 17^th^ IHIW populations for HLA-B** |

| 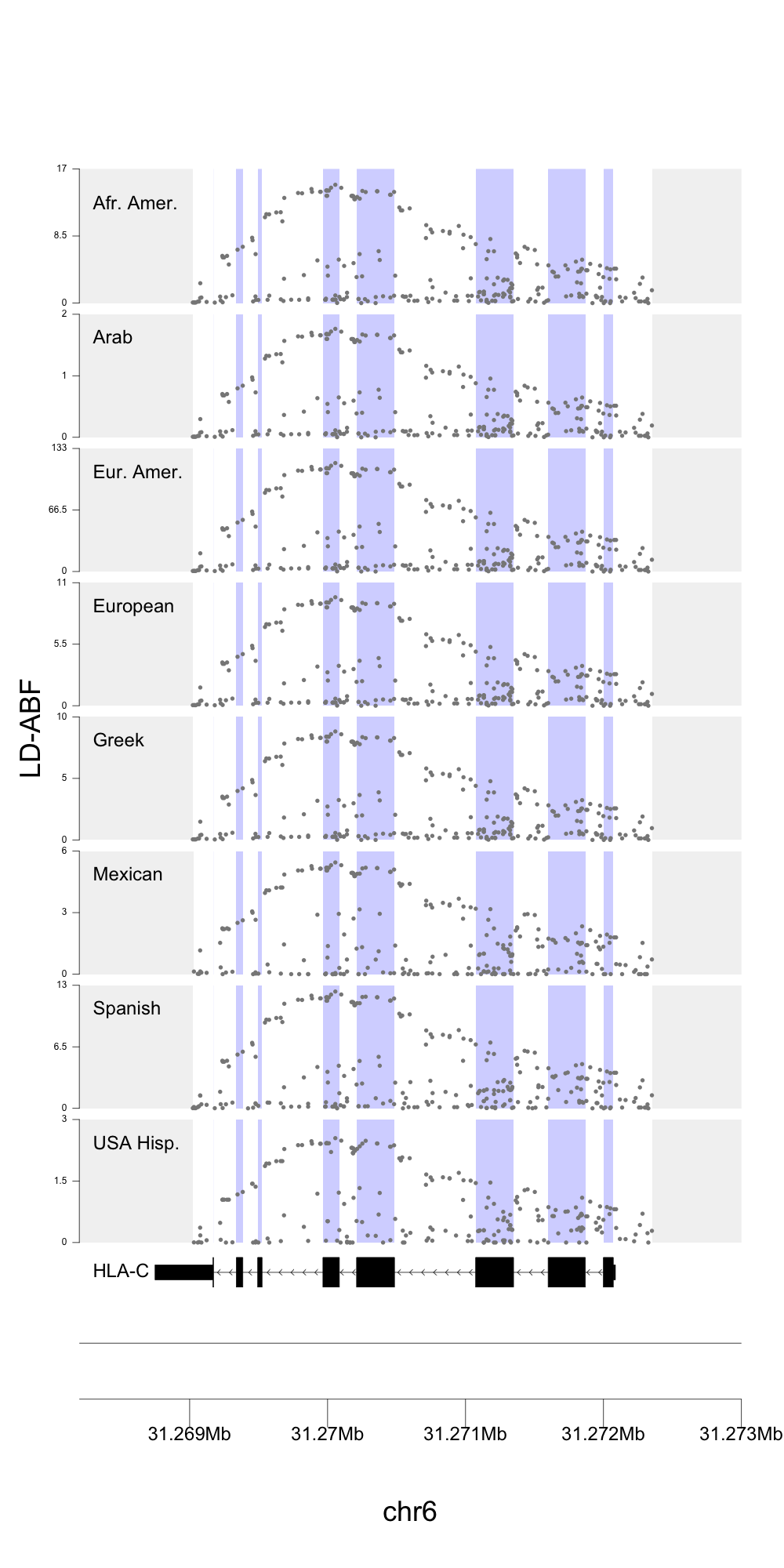 |
| --- |
| **Supplemental Figure 8 Comparison of LD-ABF across 17^th^ IHIW populations for HLA-C** |

| 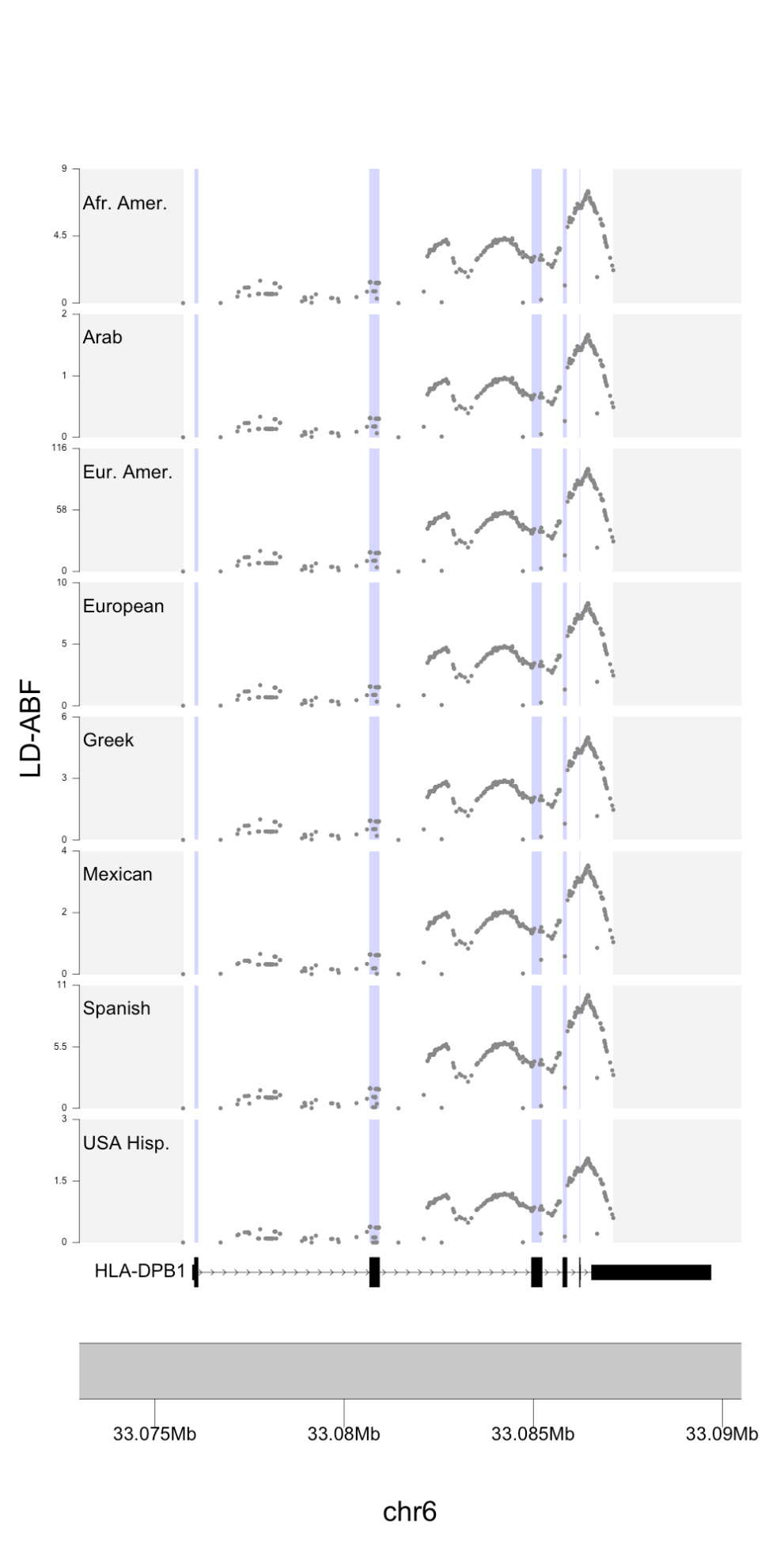 |
| --- |
| **Supplemental Figure 9 Comparison of LD-ABF across 17^th^ IHIW populations for HLA-DPB1** |

| 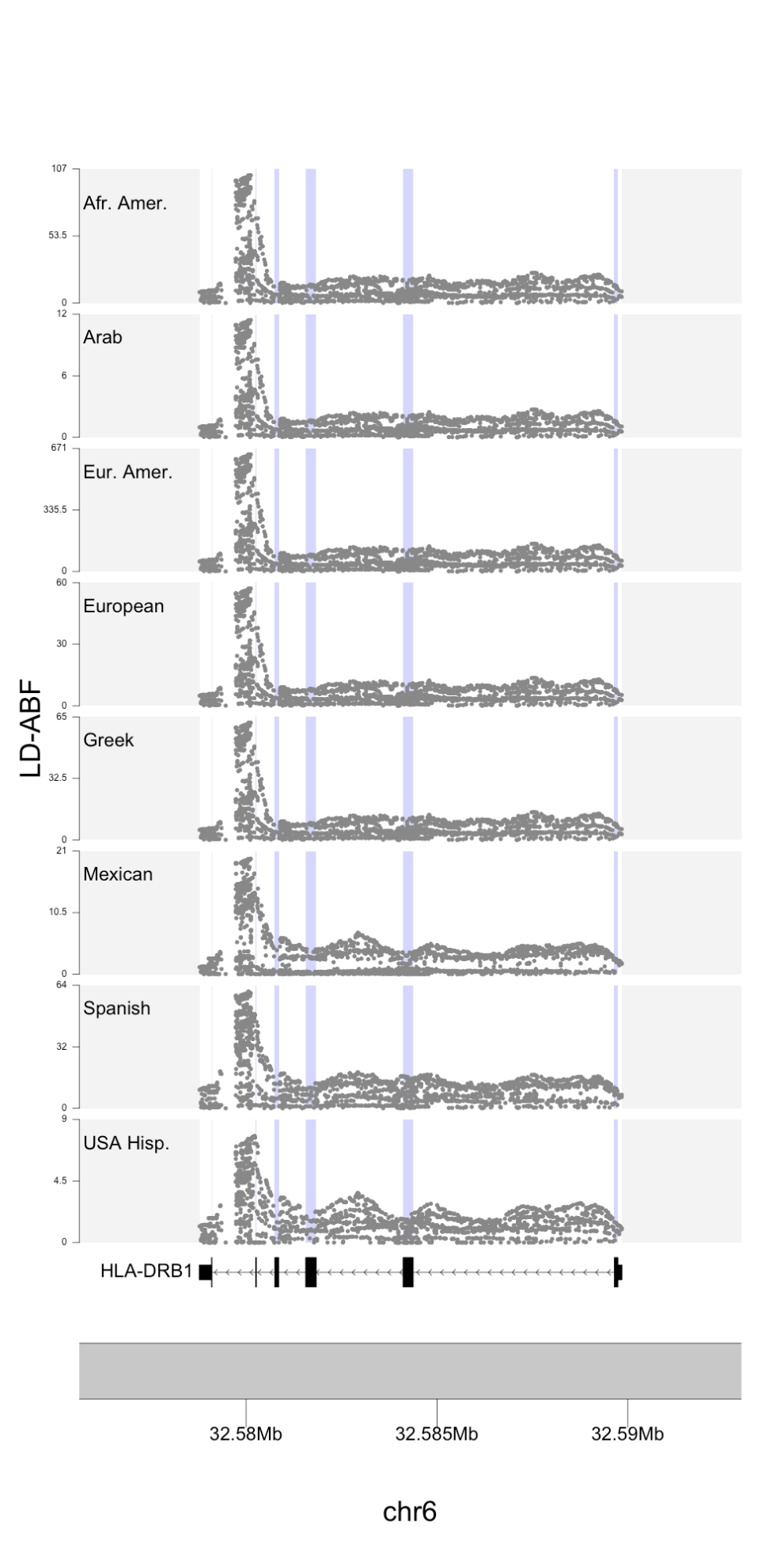 |
| --- |
| **Supplemental Figure 10 Comparison of LD-ABF across 17^th^ IHIW populations for HLA-DRB1** |

| **C**  **B**  **A**   \| 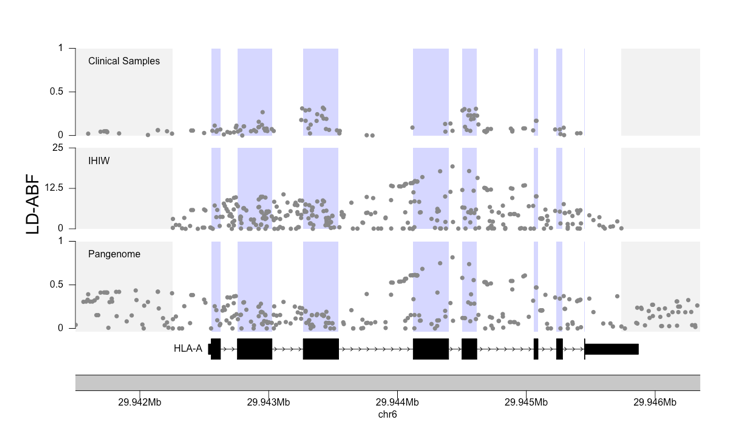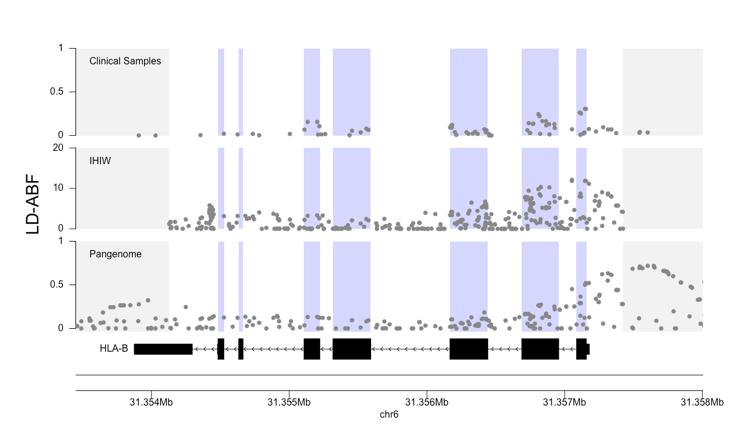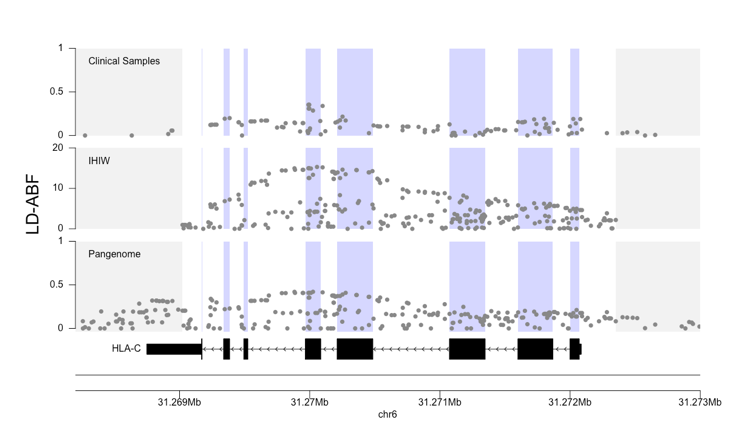  **A**  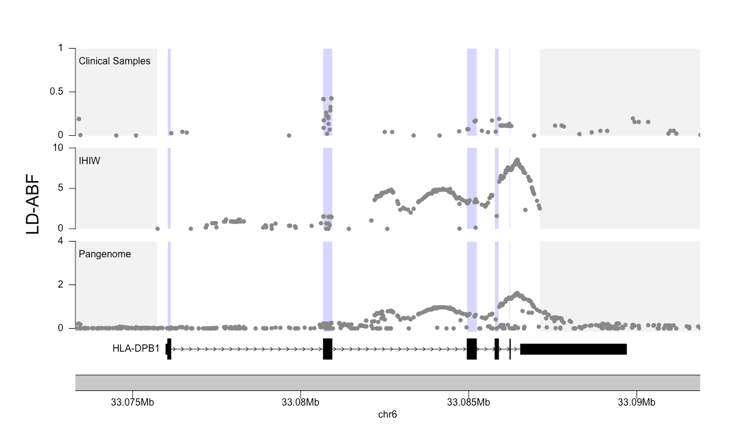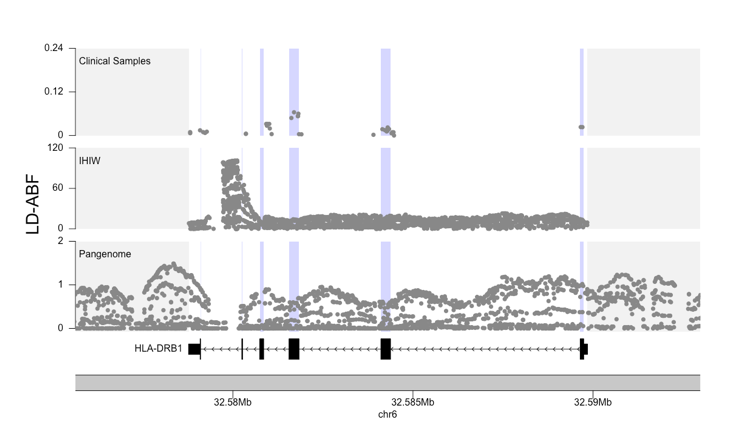  **E**  **D** \| \| --- \| |
| --- | --- |
| **Supplemental Figure 11 Balancing selection comparison over HLA genes in the clinical samples, 17^th^ IHIW, and Pangenome**. Looking in the African American samples within the three different data sets in the 17^th^ IHIW HLA genes A) HLA-A, B) HLA-B, C) HLA-C, D) DPB1, and E) DRB1, while DQA1 and DQB1 can be found in Figure 3. |

| 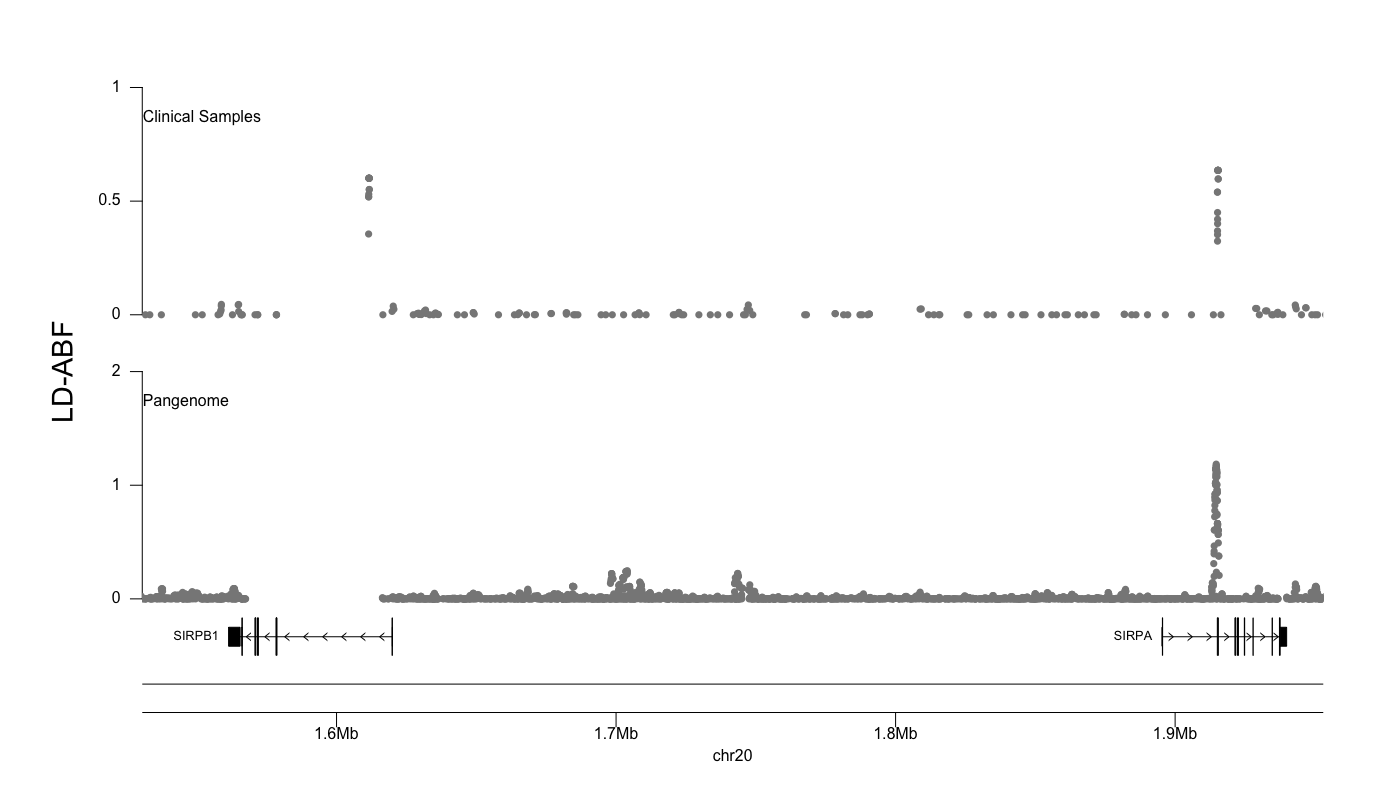 |
| --- |
| **Supplemental Figure 12 Comparison of LD-ABF over the SIRP region between clinical samples and the Pangenome.** The clinical samples are in the top pane which is based off combined exome and SNP array sequencing versus the Pangenome with high quality long-read sequencing. The signal over SIRPB1 does not appear in the Pangenome samples, coupled with the knowledge that the gene has structure variation within it suggesting the signal in the clinical samples is likely artifactual due to mapping or alignment issues. |

| 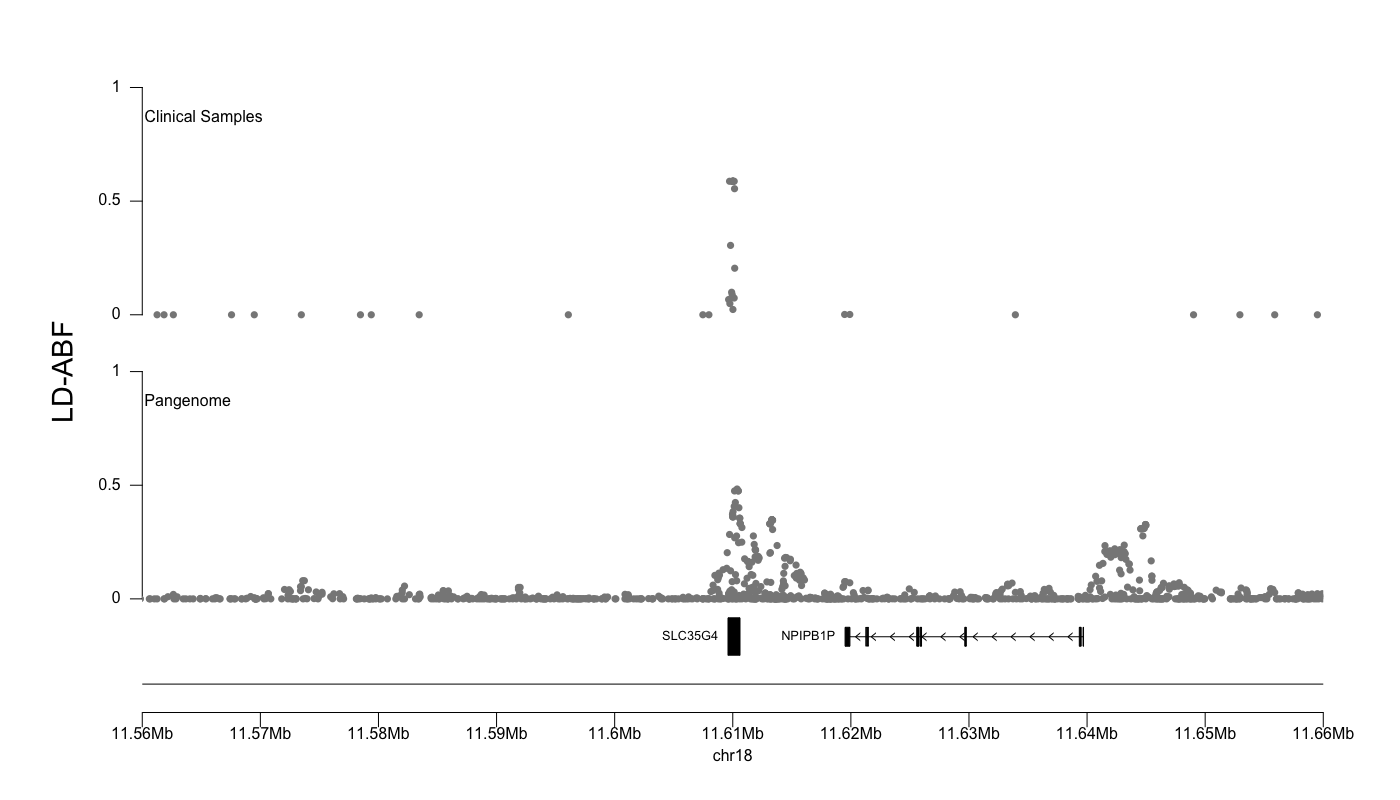 |
| --- |
| **Supplemental Figure 13**  **Exploring Possible Selection Signals in SLC35G4 in AFR Samples from CHOP Trios and the Pangenome Prior to Mappability Filters.** Both samples show a peak in LD density within the solute carrier gene. In an earlier run before applying mappability filters, the third strongest LD-ABF selection signal in the AFR samples was SLC35G4, which encodes for a putative solute carrier. SLC35G4 was identified as a top 100 peak by three of the four methods as well. To our knowledge the strong selection signal in SLC35G4 is not previously reported in the literature. The finding was further supported by the Pangenome analysis with long read sequencing data that likely was not hindered by the same mappability concerns and corroborated by other methods before filtering. So, we caution this is possibly an untrustworthy signal because it’s in a difficult region that we filter out, but this also highlights the potential that using existing filters may remove real signatures of selection. SLC35G4 belongs to the solute carrier family of genes, several other genes in this family have been found to be under selection (Bitarello et al. 2018; Cheng and Degiorgio 2019; Palamara et al. 2018; Tennessen and Duraisingh 2021). Although minimally studied to date, SLC35G4 has recently been described as a potential neoantigen in prostate cancer (Chen et al. 2020). |

| 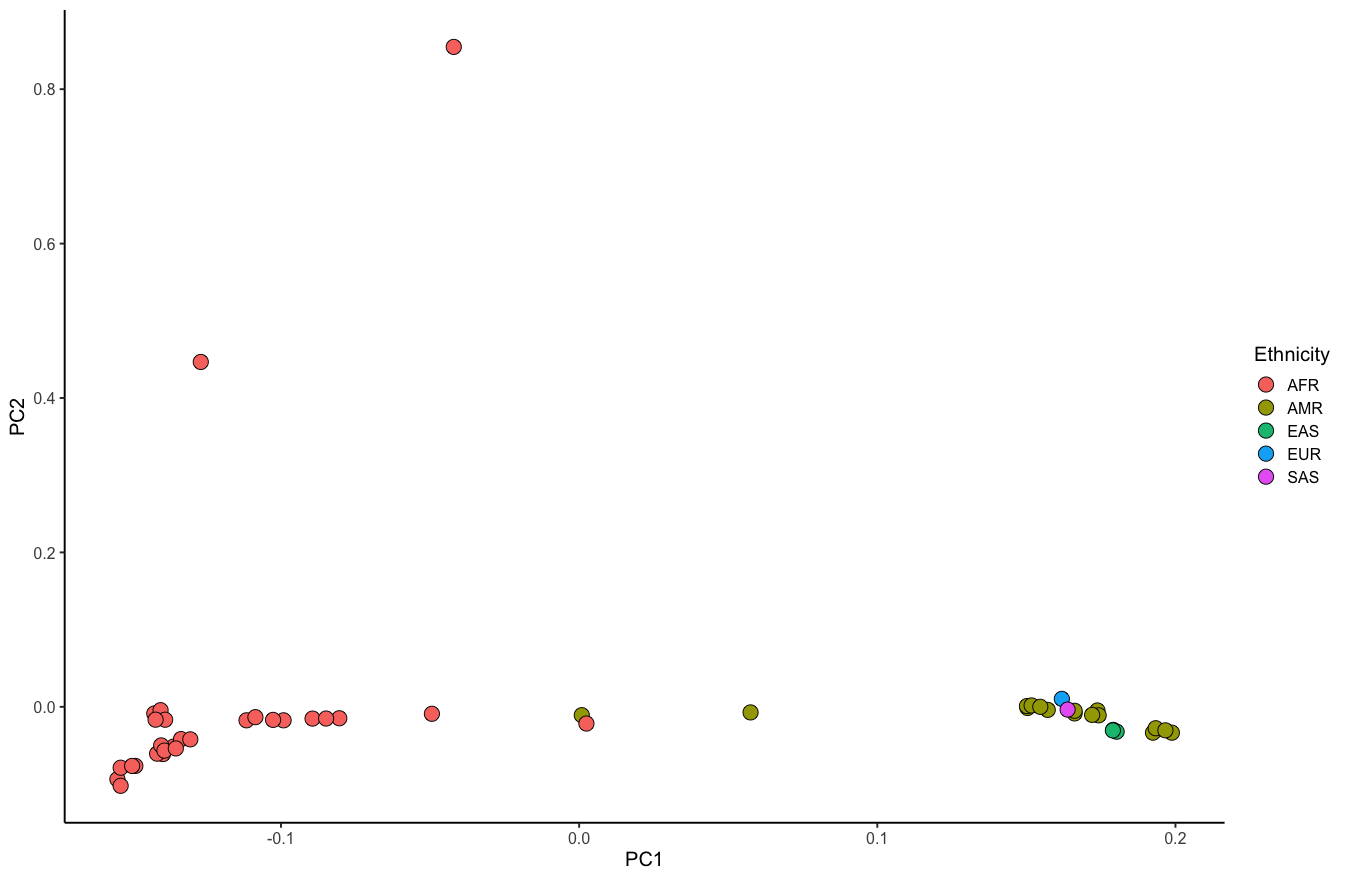 |
| --- |
| **Supplemental Figure 14** **PCA analysis of Pangenome samples including the two outlier African samples before they were removed.** |

| 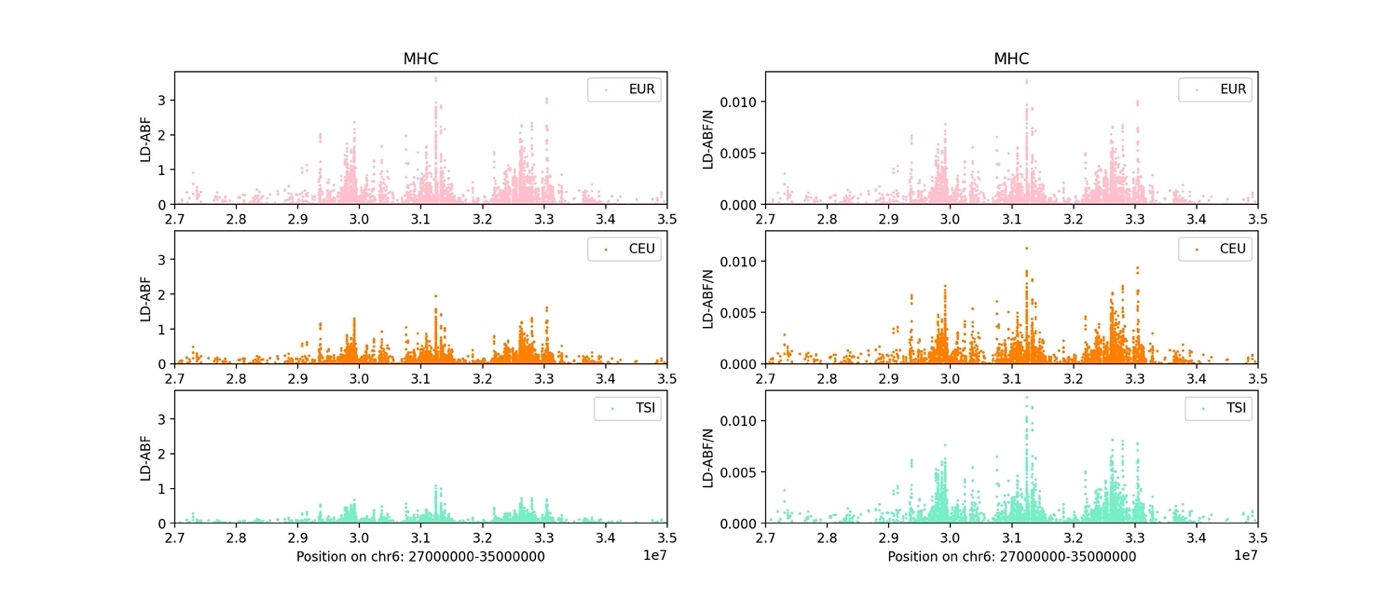  **A**  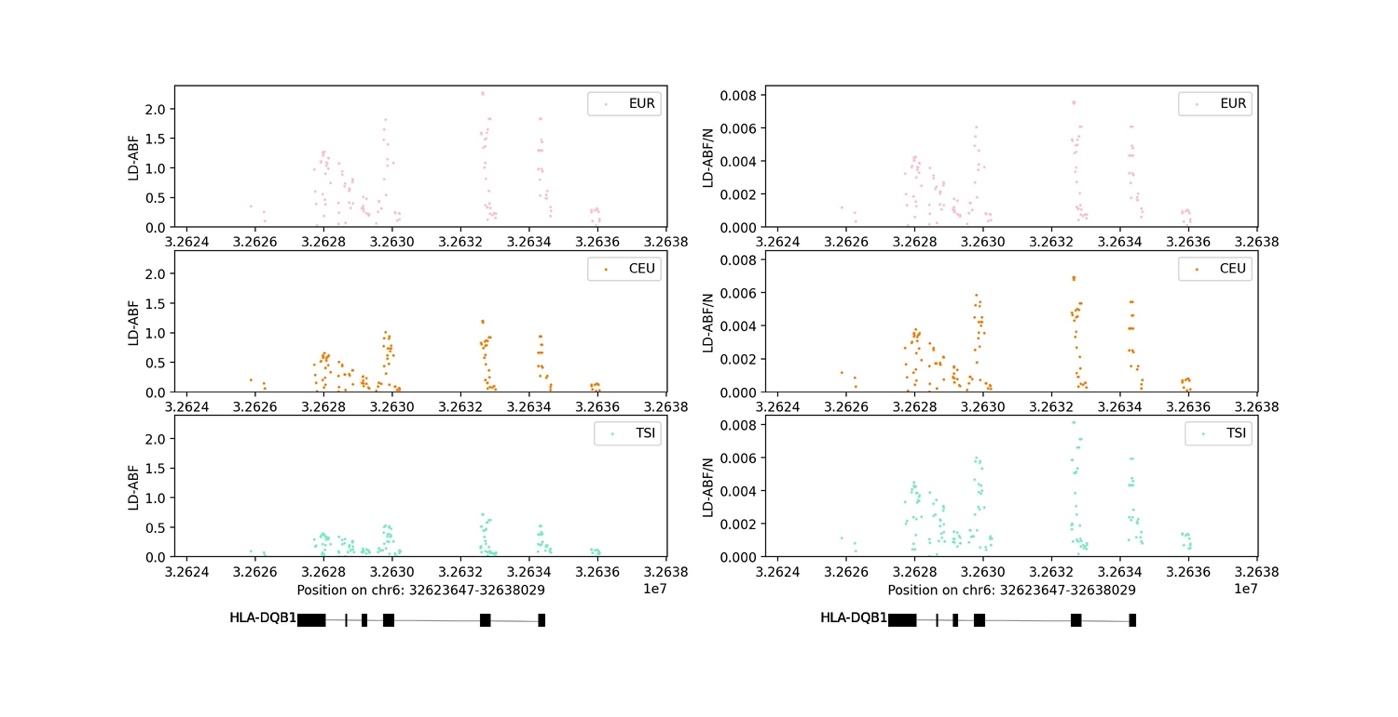  **D**  **C** |
| --- |
| **Supplemental Figure 15 LD-ABF selection scan across EUR clinical samples and subpopulations in the MHC and in HLA-DQA1.** For the EUR samples the first 10 principal components (PCs) were calculated, K-nearest neighbors clustering algorithm was run to group samples by their best matching subpopulation in 1KGP. This resulted in clustering of 88 Italian and 173 north western EUR, the remain set were 2 FIN, and the rest were outliers with apparent strong admixture so they could not be grouped into discrete subpopulations. The scan is shown across the MHC (A and B) and then zoomed in on HLA-DQB1 (C and D) which had the strongest signal within the HLA genes. Since the population sizes are different the right plots (B and D) are scaled by the within population sample size, N. The two subpopulations appear to match up closely to each other and the fit with the entire European sample. |

#

**B**

| Time | Allele Freq | Statistic | Precision | Recall | F1 | AUC |
| --- | --- | --- | --- | --- | --- | --- |
| Older | 25 | LD-ABF | 0.948 | 0.899 | 0.923 | 0.981 |
|  |  | D_ng_ | 0.947 | 0.900 | 0.923 | 0.982 |
|  |  | $\beta$ | 0.943 | 0.827 | 0.881 | 0.964 |
|  |  | $\beta_{2,std}$ | 0.946 | 0.862 | 0.902 | 0.975 |
|  |  | HKA | 0.918 | 0.559 | 0.694 | 0.850 |
|  |  | Tajima’s D | 0.900 | 0.452 | 0.601 | 0.893 |
|  |  | B2 | 0.935 | 0.722 | 0.815 | 0.919 |
|  | 50 | LD-ABF | 0.949 | 0.917 | 0.933 | 0.983 |
|  |  | D_ng_ | 0.946 | 0.869 | 0.905 | 0.974 |
|  |  | $\beta$ | 0.943 | 0.824 | 0.879 | 0.964 |
|  |  | $\beta_{2,std}$ | 0.944 | 0.838 | 0.888 | 0.969 |
|  |  | HKA | 0.909 | 0.498 | 0.643 | 0.830 |
|  |  | Tajima’s D | 0.942 | 0.805 | 0.868 | 0.963 |
|  |  | B2 | 0.938 | 0.759 | 0.839 | 0.927 |
|  | 75 | LD-ABF | 0.947 | 0.888 | 0.916 | 0.979 |
|  |  | D_ng_ | 0.948 | 0.894 | 0.920 | 0.980 |
|  |  | $\beta$ | 0.943 | 0.823 | 0.879 | 0.959 |
|  |  | $\beta_{2,std}$ | 0.945 | 0.850 | 0.895 | 0.972 |
|  |  | HKA | 0.909 | 0.497 | 0.642 | 0.817 |
|  |  | Tajima’s D | 0.895 | 0.427 | 0.578 | 0.889 |
|  |  | B2 | 0.935 | 0.718 | 0.812 | 0.914 |
| Young | 25 | LD-ABF | 0.929 | 0.655 | 0.768 | 0.934 |
|  |  | D_ng_ | 0.930 | 0.659 | 0.771 | 0.933 |
|  |  | $\beta$ | 0.914 | 0.532 | 0.673 | 0.887 |
|  |  | $\beta_{2,std}$ | 0.920 | 0.573 | 0.706 | 0.909 |
|  |  | HKA | 0.814 | 0.216 | 0.341 | 0.671 |
|  |  | Tajima’s D | 0.831 | 0.246 | 0.379 | 0.821 |
|  |  | B2 | 0.875 | 0.349 | 0.499 | 0.771 |
|  | 50 | LD-ABF | 0.934 | 0.711 | 0.808 | 0.944 |
|  |  | D_ng_ | 0.924 | 0.609 | 0.734 | 0.924 |
|  |  | $\beta$ | 0.916 | 0.543 | 0.681 | 0.897 |
|  |  | $\beta_{2,std}$ | 0.916 | 0.540 | 0.680 | 0.905 |
|  |  | HKA | 0.838 | 0.259 | 0.396 | 0.684 |
|  |  | Tajima’s D | 0.922 | 0.589 | 0.719 | 0.919 |
|  |  | B2 | 0.895 | 0.425 | 0.577 | 0.795 |
|  | 75 | LD-ABF | 0.925 | 0.621 | 0.743 | 0.928 |
|  |  | D_ng_ | 0.927 | 0.631 | 0.750 | 0.926 |
|  |  | $\beta$ | 0.906 | 0.484 | 0.631 | 0.880 |
|  |  | $\beta_{2,std}$ | 0.915 | 0.537 | 0.677 | 0.904 |
|  |  | HKA | 0.806 | 0.205 | 0.327 | 0.662 |
|  |  | Tajima’s D | 0.812 | 0.216 | 0.341 | 0.816 |
|  |  | B2 | 0.878 | 0.358 | 0.509 | 0.772 |
| Recent | 25 | LD-ABF | 0.453 | 0.041 | 0.074 | 0.628 |
|  |  | D_ng_ | 0.450 | 0.041 | 0.074 | 0.604 |
|  |  | $\beta$ | 0.390 | 0.032 | 0.059 | 0.496 |
|  |  | $\beta_{2,std}$ | 0.411 | 0.035 | 0.064 | 0.546 |
|  |  | HKA | 0.603 | 0.076 | 0.135 | 0.507 |
|  |  | Tajima’s D | 0.583 | 0.070 | 0.125 | 0.627 |
|  |  | B2 | 0.647 | 0.092 | 0.160 | 0.581 |
|  | 50 | LD-ABF | 0.697 | 0.115 | 0.197 | 0.674 |
|  |  | D_ng_ | 0.587 | 0.071 | 0.127 | 0.633 |
|  |  | $\beta$ | 0.528 | 0.056 | 0.101 | 0.511 |
|  |  | $\beta_{2,std}$ | 0.490 | 0.048 | 0.087 | 0.530 |
|  |  | HKA | 0.633 | 0.086 | 0.151 | 0.521 |
|  |  | Tajima’s D | 0.754 | 0.154 | 0.255 | 0.717 |
|  |  | B2 | 0.649 | 0.092 | 0.162 | 0.569 |
|  | 75 | LD-ABF | 0.626 | 0.083 | 0.147 | 0.625 |
|  |  | D_ng_ | 0.622 | 0.082 | 0.144 | 0.617 |
|  |  | $\beta$ | 0.611 | 0.079 | 0.139 | 0.533 |
|  |  | $\beta_{2,std}$ | 0.596 | 0.073 | 0.130 | 0.531 |
|  |  | HKA | 0.623 | 0.081 | 0.143 | 0.514 |
|  |  | Tajima’s D | 0.346 | 0.027 | 0.049 | 0.545 |
|  |  | B2 | 0.576 | 0.068 | 0.122 | 0.538 |

# Supplementary Tables

**Supplemental Table 1 Comparing methods for detecting balancing selection alleles versus neutral alleles in simulated scenarios of variable equilibrium frequency and mutation timing.** Other than the AUC the statistics were calculated at a FPR of 5%. This corresponds to the same sets of simulations found in Supplemental Figure 2 similar to previous studies (Siewert and Voight 2017, 2020).

| Window Size | Statistic | Precision | Recall | F1 | AUC |
| --- | --- | --- | --- | --- | --- |
| 100 | LD-ABF | 0.896 | 0.432 | 0.583 | 0.741 |
|  | D_ng_ | 0.893 | 0.418 | 0.569 | 0.709 |
|  | $\beta$ | 0.892 | 0.415 | 0.566 | 0.658 |
|  | $\beta_{2,std}$ | 0.892 | 0.412 | 0.563 | 0.718 |
|  | HKA | 0.666 | 0.099 | 0.173 | 0.560 |
|  | Tajima’s D | 0.912 | 0.518 | 0.661 | 0.888 |
|  | B2 | 0.837 | 0.258 | 0.394 | 0.711 |
| 500 | LD-ABF | 0.936 | 0.727 | 0.818 | 0.932 |
|  | D_ng_ | 0.924 | 0.607 | 0.733 | 0.915 |
|  | $\beta$ | 0.917 | 0.556 | 0.692 | 0.873 |
|  | $\beta_{2,std}$ | 0.920 | 0.575 | 0.708 | 0.892 |
|  | HKA | 0.815 | 0.221 | 0.347 | 0.654 |
|  | Tajima’s D | 0.916 | 0.548 | 0.686 | 0.905 |
|  | B2 | 0.888 | 0.398 | 0.550 | 0.788 |
| 1000 | LD-ABF | 0.938 | 0.756 | 0.837 | 0.952 |
|  | D_ng_ | 0.931 | 0.679 | 0.786 | 0.935 |
|  | $\beta$ | 0.921 | 0.585 | 0.716 | 0.905 |
|  | $\beta_{2,std}$ | 0.923 | 0.597 | 0.725 | 0.917 |
|  | HKA | 0.799 | 0.199 | 0.319 | 0.655 |
|  | Tajima’s D | 0.921 | 0.580 | 0.711 | 0.915 |
|  | B2 | 0.890 | 0.403 | 0.555 | 0.790 |
| 5000 | LD-ABF | 0.928 | 0.644 | 0.761 | 0.949 |
|  | D_ng_ | 0.914 | 0.535 | 0.675 | 0.925 |
|  | $\beta$ | 0.907 | 0.486 | 0.633 | 0.883 |
|  | $\beta_{2,std}$ | 0.908 | 0.496 | 0.641 | 0.901 |
|  | HKA | 0.572 | 0.067 | 0.120 | 0.516 |
|  | Tajima’s D | 0.903 | 0.464 | 0.613 | 0.891 |
|  | B2 | 0.889 | 0.401 | 0.552 | 0.790 |

**Supplemental Table 2 Comparison of methods ability to detect balancing alleles across various window size in simulation.** Other than the AUC the statistics were calculated at a FPR of 5%. All test statistics were calculated using window size of 100, 500, 1,000 or 5,000 base pairs. The corresponding simulations were performed with younger mutations (100,000 generations back) at an equilibrium frequency of 50 and a selection coefficient of 10^-4^. True positives are taken from the 2,000 balancing selection simulations for each plot and a random common neutral mutation (with MAF>5%) is used from each of the 2,000 neutral simulations to compare as a false positive. This corresponds to the same sets of simulations found in Supplemental Figure 3.

| Pop | Chr | Start | End | LD- ABF | Gene | Gene Category | # Genes  within  100Kb | # Genes  within  1Mb |
| --- | --- | --- | --- | --- | --- | --- | --- | --- |
| AFR | 11 | 5373251 | 5373251 | *0.69* | OR51B6 | Olfactory Receptor Family | 4 | 50 |
|  | 6 | 33037080 | 33037082 | *0.59* | HLA-DPA1 | Major Histocompatibility Complex, Class II | 3 | 48 |
|  | 1 | 158725194 | 158725194 | *0.57* | OR6K6 | Olfactory Receptor Family | 4 | 26 |
|  | 20 | 1895963 | 1896100 | *0.56* | SIRPA | Signal regulatory protein | 2 | 15 |
|  | 11 | 4790671 | 4790671 | *0.54* | MMP26, OR51F1 | Olfactory Receptor Family | 2 | 40 |
|  | 13 | 93969248 | 93969473 | *0.46* | GPC6 | Glypican | 0 | 3 |
|  | 22 | 22869123 | 22869218 | *0.45* | ZNF280A | Zinc Finger Protein | 4 | 12 |
|  | 6 | 31380091 | 31380091 | *0.43* | MICA | Major Histocompatibility Complex, Class I | 3 | 82 |
|  | 11 | 7817852 | 7817959 | *0.43* | OR5P2 | Olfactory Receptor Family | 2 | 19 |
|  | 19 | 37488197 | 37488197 | *0.40* | ZNF568 | Zinc fingers | 1 | 25 |
| AMR | 20 | 1895963 | 1895963 | *0.93* | SIRPA | Signal regulatory protein | 2 | 15 |
|  | 6 | 31237802 | 31237802 | *0.81* | HLA-C | Major Histocompatibility Complex, Class I | 2 | 73 |
|  | 11 | 5443887 | 5443887 | *0.69* | OR51B5, OR51Q1 | Olfactory Receptor Family | 4 | 49 |
|  | 11 | 7817852 | 7817856 | *0.65* | OR5P2 | Olfactory Receptor Family | 2 | 19 |
|  | 6 | 33037412 | 33037424 | *0.64* | HLA-DPA1 | Major Histocompatibility Complex, Class II | 3 | 48 |
|  | 1 | 24201448 | 24201448 | *0.53* | CNR2 | Cannabinoid receptor | 3 | 24 |
|  | 13 | 93969248 | 93969473 | *0.53* | GPC6 | Glypican | 0 | 3 |
|  | 22 | 22869123 | 22869218 | *0.52* | ZNF280A | Zinc Finger Protein | 4 | 12 |
|  | 19 | 37488197 | 37488197 | *0.51* | ZNF568 | Zinc fingers | 1 | 25 |
|  | 1 | 248525328 | 248525330 | *0.48* | OR2T4 | Olfactory Receptor Family | 5 | 37 |
| EAS | 20 | 1895984 | 1896060 | *0.40* | SIRPA | Signal regulatory protein | 2 | 15 |
|  | 6 | 33037412 | 33037412 | *0.35* | HLA-DPA1 | Major Histocompatibility Complex, Class II | 3 | 48 |
|  | 6 | 31237802 | 31237802 | *0.26* | HLA-C | Major Histocompatibility Complex, Class I | 2 | 73 |
|  | 11 | 5443887 | 5443887 | *0.26* | OR51B5, OR51Q1 | Olfactory Receptor Family | 4 | 49 |
|  | 1 | 248525328 | 248525330 | *0.26* | OR2T4 | Olfactory Receptor Family | 5 | 37 |
|  | 1 | 89652071 | 89652090 | *0.23* | GBP4 | Guanylate-binding proteins | 2 | 16 |
|  | 14 | 105418234 | 105418235 | *0.23* | AHNAK2 | PDZ domain containing | 3 | 27 |
|  | 22 | 22869123 | 22869218 | *0.23* | ZNF280A | Zinc Finger Protein | 4 | 12 |
|  | 1 | 24201448 | 24201448 | *0.22* | CNR2 | Cannabinoid receptor | 3 | 24 |
|  | 6 | 159654994 | 159654994 | *0.21* | FNDC1 | fibronectin type III domain containing 1 | 2 | 14 |
| EUR | 20 | 1895963 | 1895963 | *3.93* | SIRPA | Signal regulatory protein | 2 | 15 |
|  | 6 | 31237876 | 31237876 | *3.64* | HLA-C | Major Histocompatibility Complex, Class I | 2 | 73 |
|  | 11 | 5443887 | 5443887 | *3.21* | OR51B5, OR51Q1 | Olfactory Receptor Family | 4 | 49 |
|  | 12 | 11244390 | 11244390 | *3.15* | PRH1, PRH1-PRR4, PRH1-TAS2R14, TAS2R43 | Heterogeneous family of proline-rich salivary glycoproteins, taste receptor | 3 | 31 |
|  | 6 | 33037419 | 33037424 | *3.04* | HLA-DPA1 | Major Histocompatibility Complex, Class II | 3 | 48 |
|  | 13 | 93969248 | 93969473 | *2.92* | GPC6 | Glypican | 0 | 3 |
|  | 11 | 244106 | 244167 | *2.88* | PSMD13 | 26S Proteasome, a multicatalytic proteinase | 9 | 36 |
|  | 1 | 24201448 | 24201448 | *2.84* | CNR2 | Cannabinoid receptor | 3 | 24 |
|  | 11 | 7817852 | 7817959 | *2.82* | OR5P2 | Olfactory Receptor Family | 2 | 19 |
|  | 19 | 37488197 | 37488197 | *2.74* | ZNF568 | Zinc fingers | 1 | 25 |
| SAS | 6 | 33037412 | 33037424 | *0.31* | HLA-DPA1 | Major Histocompatibility Complex, Class II | 3 | 48 |
|  | 20 | 1895963 | 1895990 | *0.25* | SIRPA | Signal regulatory protein | 2 | 15 |
|  | 12 | 11244378 | 11244390 | *0.20* | PRH1, PRH1-PRR4, PRH1-TAS2R14, TAS2R43 | Heterogeneous family of proline-rich salivary glycoproteins, taste receptor | 3 | 31 |
|  | 1 | 248525328 | 248525330 | *0.19* | OR2T4 | Olfactory Receptor Family | 5 | 37 |
|  | 11 | 5443887 | 5443887 | *0.17* | OR51B5, OR51Q1 | Olfactory Receptor Family | 4 | 49 |
|  | 1 | 24201448 | 24201448 | *0.17* | CNR2 | Cannabinoid receptor | 3 | 24 |
|  | 13 | 93969248 | 93969473 | *0.16* | GPC6 | Glypican | 0 | 3 |
|  | 2 | 234622061 | 234622110 | *0.15* | UGT1A10, UGT1A5, UGT1A6, UGT1A7, UGT1A8, UGT1A9 | UDP-glucuronosyltransferase | 9 | 22 |
|  | 2 | 231047645 | 231047662 | *0.15* | SP110 | Bromodomain containing | 2 | 8 |
|  | 22 | 22869123 | 22869218 | *0.15* | ZNF280A | Zinc Finger Protein | 4 | 12 |

**Supplemental Table 3** **Top 10 genome wide peaks in balancing selection signal in each clinical sample population.** Peaks reported using 1Mb neighbor hoods with genic context and regional gene density**.** For instances where the exact peak position occurs at multiple variants within a region in perfect LD, the start and end positions are represented here, and each individual variant can be found in the online data.

| Pop | Chr | Start | End | LD- ABF | Gene | Gene Category | # Genes  within  100Kb | # Genes  within  1Mb |
| --- | --- | --- | --- | --- | --- | --- | --- | --- |
| AFR | 11 | 5373251 | 5373251 | *0.69* | OR51B6 | Olfactory Receptor Family | 4 | 50 |
|  | 6 | 33037080 | 33037082 | *0.59* | HLA-DPA1 | Major Histocompatibility Complex, Class II | 3 | 48 |
|  | 1 | 158725194 | 158725194 | *0.57* | OR6K6 | Olfactory Receptor Family | 4 | 26 |
|  | 20 | 1895963 | 1896100 | *0.56* | SIRPA | Signal regulatory protein | 2 | 15 |
|  | 11 | 4790671 | 4790671 | *0.54* | MMP26, OR51F1 | Olfactory Receptor Family | 2 | 40 |
|  | 11 | 5443887 | 5443887 | *0.51* | OR51B5, OR51Q1 | Olfactory Receptor Family | 4 | 49 |
|  | 20 | 1592193 | 1592349 | *0.48* | SIRPB1 | CD molecules | 3 | 23 |
|  | 13 | 93969248 | 93969473 | *0.46* | GPC6 | Glypican | 0 | 3 |
|  | 22 | 22869123 | 22869218 | *0.45* | ZNF280A | Zinc Finger Protein | 4 | 12 |
|  | 6 | 31380091 | 31380091 | *0.43* | MICA | Major Histocompatibility Complex, Class I | 3 | 82 |
| AMR | 20 | 1895963 | 1895963 | *0.93* | SIRPA | Signal regulatory protein | 2 | 15 |
|  | 6 | 31237802 | 31237802 | *0.81* | HLA-C | Major Histocompatibility Complex, Class I | 2 | 73 |
|  | 11 | 5443887 | 5443887 | *0.69* | OR51B5, OR51Q1 | Olfactory Receptor Family | 4 | 49 |
|  | 11 | 5373242 | 5373242 | *0.67* | OR51B6 | Olfactory Receptor Family | 4 | 50 |
|  | 11 | 7817852 | 7817856 | *0.65* | OR5P2 | Olfactory Receptor Family | 2 | 19 |
|  | 6 | 33037412 | 33037424 | *0.64* | HLA-DPA1 | Major Histocompatibility Complex, Class II | 3 | 48 |
|  | 6 | 32610008 | 32610008 | *0.56* | HLA-DQA1 | Histocompatibility complex | 3 | 41 |
|  | 20 | 1592215 | 1592284 | *0.54* | SIRPB1 | CD molecules | 3 | 23 |
|  | 1 | 24201448 | 24201448 | *0.53* | CNR2 | Cannabinoid receptor | 3 | 24 |
|  | 13 | 93969248 | 93969473 | *0.53* | GPC6 | Glypican | 0 | 3 |
| EAS | 20 | 1895984 | 1896060 | *0.40* | SIRPA | Signal regulatory protein | 2 | 15 |
|  | 6 | 33037412 | 33037412 | *0.35* | HLA-DPA1 | Major Histocompatibility Complex, Class II | 3 | 48 |
|  | 20 | 1592215 | 1592312 | *0.30* | SIRPB1 | CD molecules | 3 | 23 |
|  | 6 | 31237802 | 31237802 | *0.26* | HLA-C | Major Histocompatibility Complex, Class I | 2 | 73 |
|  | 11 | 5443887 | 5443887 | *0.26* | OR51B5, OR51Q1 | Olfactory Receptor Family | 4 | 49 |
|  | 1 | 248525328 | 248525330 | *0.26* | OR2T4 | Olfactory Receptor Family | 5 | 37 |
|  | 11 | 5862653 | 5863013 | *0.25* | OR52E6 | 7TM proteins | 4 | 41 |
|  | 1 | 89652071 | 89652090 | *0.23* | GBP4 | Guanylate-binding proteins | 2 | 16 |
|  | 14 | 105418234 | 105418235 | *0.23* | AHNAK2 | PDZ domain containing | 3 | 27 |
|  | 22 | 22869123 | 22869218 | *0.23* | ZNF280A | Zinc Finger Protein | 4 | 12 |
| EUR | 20 | 1895963 | 1895963 | *3.93* | SIRPA | Signal regulatory protein | 2 | 15 |
|  | 6 | 31237876 | 31237876 | *3.64* | HLA-C | Major Histocompatibility Complex, Class I | 2 | 73 |
|  | 11 | 5443887 | 5443887 | *3.21* | OR51B5, OR51Q1 | Olfactory Receptor Family | 4 | 49 |
|  | 11 | 5373242 | 5373242 | *3.16* | OR51B6 | Olfactory Receptor Family | 4 | 50 |
|  | 12 | 11244390 | 11244390 | *3.15* | PRH1, PRH1-PRR4, PRH1-TAS2R14, TAS2R43 | Heterogeneous family of proline-rich salivary glycoproteins, taste receptor | 3 | 31 |
|  | 6 | 33037419 | 33037424 | *3.04* | HLA-DPA1 | Major Histocompatibility Complex, Class II | 3 | 48 |
|  | 20 | 1592312 | 1592312 | *2.95* | SIRPB1 | CD molecules | 3 | 23 |
|  | 13 | 93969248 | 93969473 | *2.92* | GPC6 | Glypican | 0 | 3 |
|  | 11 | 244106 | 244167 | *2.88* | PSMD13 | 26S Proteasome, a multicatalytic proteinase | 9 | 36 |
|  | 1 | 24201448 | 24201448 | *2.84* | CNR2 | Cannabinoid receptor | 3 | 24 |
| SAS | 6 | 33037412 | 33037424 | *0.31* | HLA-DPA1 | Major Histocompatibility Complex, Class II | 3 | 48 |
|  | 20 | 1895963 | 1895990 | *0.25* | SIRPA | Signal regulatory protein | 2 | 15 |
|  | 12 | 11244378 | 11244390 | *0.20* | PRH1, PRH1-PRR4, PRH1-TAS2R14, TAS2R43 | Heterogeneous family of proline-rich salivary glycoproteins, taste receptor | 3 | 31 |
|  | 1 | 248525328 | 248525330 | *0.19* | OR2T4 | Olfactory Receptor Family | 5 | 37 |
|  | 6 | 32632628 | 32632628 | *0.17* | HLA-DQB1 | Histocompatibility complex | 3 | 40 |
|  | 11 | 5443887 | 5443887 | *0.17* | OR51B5, OR51Q1 | Olfactory Receptor Family | 4 | 49 |
|  | 1 | 24201448 | 24201448 | *0.17* | CNR2 | Cannabinoid receptor | 3 | 24 |
|  | 11 | 5862780 | 5863013 | *0.16* | OR52E6 | 7TM proteins | 4 | 41 |
|  | 11 | 5373170 | 5373251 | *0.16* | OR51B6 | Olfactory Receptor Family | 5 | 50 |
|  | 20 | 1592215 | 1592312 | *0.16* | SIRPB1 | CD molecules | 3 | 23 |

**Supplemental Table 4 Top 10 genome wide peaks in balancing selection signal in each clinical sample population.** Peaks reported using 100kb neighbor hoods with genic context and regional gene density**.** For instances where the exact peak position occurs at multiple variants within a region in perfect LD, the start and end positions are represented here, and each individual variant can be found in the online data.

|  | LD-ABF | | | | | Dng | | | | | B2 | | | | | TD | | | | |
| --- | --- | --- | --- | --- | --- | --- | --- | --- | --- | --- | --- | --- | --- | --- | --- | --- | --- | --- | --- | --- |
| Genes | AF | AM | EU | EA | SA | AF | AM | EU | EA | SA | AF | AM | EU | EA | SA | AF | AM | EU | EA | SA |
| HLA-DRB5 |  |  |  |  |  |  |  |  |  |  | 6 | 13 | 6 | 9 | 4 |  |  |  |  |  |
| MDC1-AS1 |  |  |  |  |  |  |  |  |  |  | 40 | 8 |  | 12 | 49 |  |  |  |  |  |
| CENPF |  |  |  |  |  |  |  |  |  |  | 23 | 16 | 4 | 16 | 18 |  |  |  |  |  |
| HLA-DRB1 |  |  |  |  |  |  |  |  |  |  | 16 | 5 | 73 | 8 | 9 |  |  |  |  |  |
| FLG-AS1 |  |  |  |  |  |  |  |  |  |  | 2 |  | 46 | 59 |  |  |  |  |  |  |
| SSPOP |  |  |  |  |  |  |  |  |  |  |  | 60 |  |  | 8 |  |  |  |  |  |
| MUC5B |  |  |  |  |  |  |  |  |  |  | 10 | 1 |  |  | 68 |  |  |  |  |  |
| MUC22 |  |  |  |  |  |  |  |  |  |  | 12 |  | 48 | 10 | 16 |  |  |  |  |  |
| MDC1 |  |  |  |  |  |  |  |  |  |  | 40 | 8 |  | 12 | 49 |  |  |  |  |  |
| VARS2 |  |  |  |  |  |  |  |  |  |  | 19 | 37 | 9 | 13 | 14 |  |  |  |  |  |
| PLIN4 |  |  |  |  |  |  |  |  |  |  | 9 | 40 |  |  |  |  |  |  |  |  |
| HLA-DQB1-AS1 |  |  |  |  |  |  |  | 12 | 3 | 3 |  |  |  |  |  |  |  |  |  |  |
| HCG4 |  |  |  |  |  | 9 | 6 | 6 |  |  |  |  |  |  |  |  |  |  |  |  |
| HLA-V |  |  |  |  |  | 9 | 6 | 6 |  |  |  |  |  |  |  |  |  |  |  |  |
| HLA-DRB6 |  |  |  |  |  | 29 | 16 | 26 | 6 | 12 |  |  |  |  |  |  |  |  |  |  |
| OLR1 |  |  |  |  |  |  |  |  |  |  |  |  |  |  |  |  | 4 | 12 | 100 |  |
| MEIG1 |  |  |  |  |  |  |  |  |  |  |  |  |  |  |  | 8 |  | 52 |  |  |
| LDC1P |  |  |  |  |  |  |  |  |  |  |  |  |  |  |  | 17 |  | 3 |  |  |
| CCSER1 |  |  |  |  |  |  |  |  |  |  |  |  |  |  |  |  | 8 | 8 | 82 | 20 |
| MICA | 10 | 17 | 32 | 22 |  | 38 | 9 | 61 | 40 |  |  |  |  |  |  |  |  |  |  |  |
| HLA-H |  |  | 83 |  |  | 8 | 8 | 8 | 9 | 32 |  |  |  |  |  |  |  |  |  |  |
| HLA-DPB1 |  |  |  |  |  | 2 | 2 |  | 2 |  |  |  |  |  |  |  |  |  | 2 |  |
| PYROXD2 |  |  |  |  |  | 50 | 24 | 52 |  | 20 |  |  |  |  |  |  |  |  |  | 9 |
| SPATA3 |  |  |  |  |  |  |  |  | 86 | 55 |  |  |  |  |  | 46 |  |  | 37 | 10 |
| TYMS |  |  |  |  |  |  |  |  | 86 |  |  |  |  |  |  |  |  |  | 5 | 4 |
| MYO9B |  |  |  |  |  |  | 93 | 93 | 85 |  |  |  |  |  |  | 1 |  |  | 10 |  |
| LINC00668 |  |  |  |  |  |  |  |  |  | 90 |  |  |  |  |  | 47 | 15 |  | 31 | 8 |
| SPTB |  |  |  |  |  | 14 |  |  | 17 |  |  |  |  |  |  | 9 |  |  | 91 |  |
| SPINK5 |  |  |  |  |  |  | 83 | 97 |  | 67 |  |  |  |  |  |  | 85 | 90 | 50 | 5 |
| MGST3 |  |  |  |  |  |  |  |  | 85 |  |  |  |  |  |  | 7 |  |  | 10 |  |
| ENOSF1 |  |  |  |  |  |  |  |  | 86 |  |  |  |  |  |  |  |  |  | 5 | 4 |
| TRAF3IP3 |  |  |  |  |  | 70 |  |  | 76 |  |  |  |  |  |  | 5 |  |  |  |  |
| LGALS8 |  |  |  |  |  | 84 |  | 78 | 77 | 57 |  |  |  |  |  | 51 | 7 |  | 7 |  |
| CES1P1 |  |  |  |  |  | 28 | 10 | 16 |  |  |  |  |  |  |  |  |  | 31 |  |  |
| CEP170B |  |  |  |  |  |  | 53 | 65 |  | 58 |  |  |  |  |  |  | 3 | 17 |  | 1 |
| CCDC57 |  |  |  |  |  | 55 |  |  |  |  |  |  |  |  |  | 2 |  |  |  | 6 |
| FLG |  | 55 |  | 42 | 38 |  |  |  |  |  | 2 |  | 46 | 59 |  |  |  |  |  |  |
| RGMA | 43 | 20 | 34 |  | 70 |  |  |  |  |  |  |  |  |  |  | 71 | 18 | 9 |  |  |
| SUMF1 |  |  |  |  |  |  |  |  |  |  |  |  | 96 |  |  |  | 34 | 6 |  |  |
| ZNF280A | 9 | 11 | 17 | 10 | 14 | 45 | 63 | 59 | 55 | 66 | 88 | 62 | 22 | 52 | 83 |  |  |  |  |  |
| OR51F1 | 5 | 26 | 14 |  |  | 24 | 38 | 31 |  |  | 56 | 22 | 38 | 50 |  |  |  |  |  |  |
| PSMD13 | 41 | 32 | 9 | 23 | 19 | 25 | 15 | 15 | 35 | 16 | 45 | 39 | 24 | 39 | 48 |  |  |  |  |  |
| HLA-A | 40 | 24 | 21 | 21 | 33 | 4 | 7 | 3 | 5 | 8 | 5 | 3 | 3 | 1 | 1 |  |  |  |  |  |
| OR2T4 | 18 | 15 | 76 | 6 | 4 | 27 | 54 | 34 | 22 | 30 | 44 | 66 |  | 57 | 89 |  |  |  |  |  |
| HLA-DQA1 |  | 7 |  |  |  |  | 4 |  |  |  | 15 | 7 |  |  |  |  |  |  |  |  |
| OR5P2 | 11 | 5 | 12 |  |  | 20 | 12 | 25 | 29 |  | 36 | 36 | 19 | 48 | 50 |  |  |  |  |  |
| HLA-DQB1 | 33 |  | 25 | 26 | 5 | 23 |  | 12 | 3 | 3 |  |  | 7 | 4 | 5 |  |  |  |  |  |
| AHNAK2 | 77 |  | 73 | 9 | 89 | 58 | 94 |  | 21 | 44 | 1 | 2 | 10 | 3 | 2 |  |  |  |  |  |
| TAS2R43 | 44 | 16 | 5 | 70 | 3 | 11 | 25 | 9 | 51 | 7 | 8 | 14 | 23 | 15 | 11 |  |  |  |  |  |
| HLA-B | 48 | 18 | 11 |  | 18 | 6 | 5 | 5 |  | 4 | 13 | 10 | 5 | 5 | 7 |  |  |  |  |  |
| HLA-DPA1 | 2 | 6 | 6 | 2 | 1 |  |  | 7 |  | 2 | 14 | 11 | 1 | 11 | 12 |  |  |  |  |  |
| SIRPB1 | 7 | 8 | 7 | 3 | 10 | 13 | 41 | 23 | 19 | 23 | 26 | 42 | 40 | 27 | 27 |  |  |  |  |  |
| MMP26 | 5 | 26 | 14 |  |  | 24 | 38 | 31 |  |  | 56 | 22 | 38 | 50 |  |  |  |  |  |  |
| OR6K6 | 3 |  |  |  |  | 10 | 18 |  |  |  | 31 | 17 |  |  |  |  |  |  |  |  |
| OR51Q1 | 6 | 3 | 3 | 5 | 6 | 26 | 32 | 32 | 24 | 41 | 74 |  |  | 38 |  |  |  |  |  |  |
| OR51B6 | 1 | 4 | 4 |  | 9 | 3 | 13 | 10 | 12 | 11 | 25 | 34 | 21 | 20 | 17 |  |  |  |  |  |
| MYO3A |  |  | 49 |  | 66 | 44 | 35 | 18 | 20 | 24 |  |  |  |  |  | 89 |  | 1 | 6 | 28 |
| ESYT2 |  |  |  | 71 |  |  |  |  | 11 |  |  |  |  |  |  | 92 |  |  | 4 |  |
| HLA-G |  | 80 | 57 | 90 | 77 |  |  |  | 13 | 21 |  |  |  |  |  |  | 5 | 49 | 1 | 7 |
| FCER2 |  |  | 66 |  | 97 | 7 |  | 2 |  | 6 |  |  |  |  |  | 3 |  |  |  |  |
| ACSF3 | 27 | 38 | 42 | 19 | 20 | 33 | 29 | 27 | 30 | 26 |  |  |  |  |  | 18 |  |  |  | 3 |
| GPC6 | 8 | 10 | 8 | 15 | 11 | 41 | 59 | 50 | 53 | 59 |  |  |  |  |  | 26 | 17 | 4 | 60 |  |
| DMBT1 |  |  | 71 |  | 82 | 19 | 14 | 13 | 10 | 10 |  |  |  |  |  |  |  | 86 | 20 | 23 |
| RAB36 |  |  |  |  |  | 15 | 11 | 11 | 14 | 15 |  |  | 65 |  |  | 29 | 1 | 2 | 8 | 2 |
| HRNR |  |  |  | 98 | 79 |  |  |  |  |  |  |  | 92 | 87 | 73 |  | 10 |  |  |  |
| PRH1-PRR4 | 44 | 16 | 5 | 27 | 3 | 11 | 25 | 9 | 51 | 7 | 8 | 14 | 23 | 15 | 11 |  |  | 73 |  |  |
| HLA-L |  | 72 |  | 63 | 62 |  | 27 |  | 25 | 36 |  | 83 | 67 | 44 | 74 |  |  |  | 3 | 30 |
| OR52E6 | 12 | 12 | 18 | 7 | 8 | 47 | 44 | 41 | 41 | 35 | 68 | 48 | 39 | 58 | 62 |  |  |  | 46 | 97 |
| GBP4 | 38 | 31 | 15 | 8 | 24 | 31 | 48 | 44 | 44 | 47 | 70 | 50 | 18 | 46 | 60 |  |  | 67 |  |  |
| PRH1 | 44 | 16 | 5 | 27 | 3 | 11 | 25 | 9 | 51 | 7 | 8 | 14 | 23 | 15 | 11 |  |  | 73 |  |  |
| COL5A1 | 29 | 45 | 44 | 24 | 29 |  |  | 24 | 84 | 18 |  | 100 | 49 |  | 93 | 43 | 90 | 10 | 15 | 31 |
| HLA-C | 23 | 2 | 2 | 4 | 16 | 1 | 1 | 1 | 1 | 1 | 7 | 6 | 11 |  |  |  | 16 |  | 22 |  |
| OR51B5 | 1 | 3 | 3 | 5 | 6 | 3 | 13 | 10 | 12 | 11 | 25 | 34 | 21 | 20 | 17 |  |  |  | 89 |  |
| PRH1-TAS2R14 | 44 | 16 | 5 | 27 | 3 | 11 | 25 | 9 | 51 | 7 | 8 | 14 | 23 | 15 | 11 |  |  | 73 |  |  |
| HCG17 |  | 72 |  | 63 | 62 |  | 27 |  | 25 | 36 |  | 83 | 67 | 44 | 74 |  |  |  | 3 | 30 |
| MUC16 | 89 | 43 | 41 | 16 | 90 | 56 |  | 76 | 63 |  | 4 | 4 | 2 | 6 | 6 |  |  | 55 | 25 |  |
| PSCA | 52 | 29 | 35 | 73 | 34 | 22 | 30 | 20 |  | 17 |  | 92 | 55 |  |  | 4 | 6 | 5 |  |  |
| SIRPA | 4 | 1 | 1 | 1 | 2 | 5 | 3 | 4 | 4 | 5 | 21 | 46 | 35 | 14 | 19 |  | 51 |  | 36 |  |
| CNR2 | 14 | 9 | 10 | 11 | 7 | 45 | 40 | 21 | 23 | 13 | 73 | 51 | 28 | 47 | 57 |  | 2 | 7 | 35 |  |

**Supplemental Table 5 Comparison of statistics top 10 peaks of selection signal from genome wide scan of clinical samples.** Above are genes where at least one of the four methods LD-ABF, D_ng_, B2, or Tajima’s D has a top 10 selection signal identified in it where the number in each cell corresponds to the rank of that peak using a peak finding of 100Kb windows.

|  | LD-ABF | | | | | Dng | | | | | B2 | | | | | TD | | | | |
| --- | --- | --- | --- | --- | --- | --- | --- | --- | --- | --- | --- | --- | --- | --- | --- | --- | --- | --- | --- | --- |
| Genes | AF | AM | EU | EA | SA | AF | AM | EU | EA | SA | AF | AM | EU | EA | SA | AF | AM | EU | EA | SA |
| CENPF |  |  |  |  |  |  |  |  |  |  | 17 | 11 | 4 | 10 | 13 |  |  |  |  |  |
| HLA-DRB1 |  |  |  |  |  |  |  |  |  |  |  | 5 |  |  |  |  |  |  |  |  |
| HLA-DQB1 |  |  |  |  |  |  |  |  |  |  |  |  |  | 4 |  |  |  |  |  |  |
| FLG-AS1 |  |  |  |  |  |  |  |  |  |  | 2 |  | 35 | 40 |  |  |  |  |  |  |
| OR10AG1 |  |  |  |  |  |  |  |  |  |  | 60 |  | 9 |  |  |  |  |  |  |  |
| HLA-B |  |  |  |  |  |  |  |  |  |  |  |  | 5 |  |  |  |  |  |  |  |
| SSPOP |  |  |  |  |  |  |  |  |  |  |  | 46 |  | 86 | 6 |  |  |  |  |  |
| MUC5B |  |  |  |  |  |  |  |  |  |  | 10 | 1 |  |  | 50 |  |  |  |  |  |
| PLIN4 |  |  |  |  |  |  |  |  |  |  | 9 | 31 |  |  |  |  |  |  |  |  |
| HCG4 |  |  |  |  |  |  | 4 |  |  |  |  |  |  |  |  |  |  |  |  |  |
| HLA-V |  |  |  |  |  |  | 4 |  |  |  |  |  |  |  |  |  |  |  |  |  |
| HLA-DRB6 |  |  |  |  |  | 23 | 11 | 21 | 5 | 10 |  |  |  |  |  |  |  |  |  |  |
| MICA | 8 |  |  |  |  |  |  |  |  |  |  |  |  |  |  |  |  |  |  |  |
| OR51Q1 |  | 3 | 3 | 4 | 5 |  |  |  |  |  |  |  |  |  |  |  |  |  |  |  |
| HLA-G |  |  |  |  |  |  |  |  |  |  |  |  |  |  |  |  | 5 | 47 | 1 | 7 |
| CCSER1 |  |  |  |  |  |  |  |  |  |  |  |  |  |  |  |  | 8 | 8 | 75 | 20 |
| MDC1-AS1 |  |  |  |  |  |  |  |  | 80 |  | 32 | 7 |  | 7 | 35 |  |  |  |  |  |
| MDC1 |  |  |  |  |  |  |  |  | 80 |  | 32 | 7 |  | 7 | 35 |  |  |  |  |  |
| PYROXD2 |  |  |  |  |  | 39 | 18 | 40 |  | 18 |  |  |  |  |  |  |  |  |  | 9 |
| TYMS |  |  |  |  |  |  |  | 80 | 71 | 86 |  |  |  |  |  |  |  |  | 4 | 4 |
| OLR1 |  |  |  |  |  |  | 81 | 85 |  |  |  |  |  |  |  |  | 4 | 12 | 91 |  |
| MEIG1 |  |  |  |  |  |  |  |  | 89 |  |  |  |  |  |  | 8 |  | 49 |  |  |
| MYO9B |  |  |  |  |  |  | 70 | 69 | 70 |  |  |  |  |  |  | 1 |  |  | 9 |  |
| LINC00668 |  |  |  |  |  |  |  | 86 |  | 66 |  |  |  |  |  | 46 | 15 |  | 29 | 8 |
| SPTB |  |  |  |  |  | 10 |  |  | 14 |  |  |  |  |  |  | 9 |  |  | 82 |  |
| MGST3 |  |  |  |  |  | 85 |  |  | 70 |  |  |  |  |  |  | 7 |  |  | 9 |  |
| LDC1P |  |  |  |  |  |  |  | 92 |  | 100 |  |  |  |  |  | 17 |  | 3 |  |  |
| ENOSF1 |  |  |  |  |  |  |  | 80 | 71 | 86 |  |  |  |  |  |  |  |  | 4 | 4 |
| TRAF3IP3 |  |  |  |  |  | 58 |  |  | 64 |  |  |  |  |  |  | 5 |  |  |  |  |
| LGALS8 |  |  |  |  |  | 68 | 87 | 56 | 65 | 42 |  |  |  |  |  | 50 | 7 |  | 6 |  |
| CES1P1 |  |  |  |  |  | 22 | 5 | 12 |  |  |  |  |  |  |  |  |  | 29 |  |  |
| GAA |  |  |  |  |  |  |  |  | 79 |  |  |  |  |  |  |  |  |  | 10 |  |
| CEP170B |  |  |  |  |  |  | 41 | 49 |  |  |  |  |  |  |  |  | 3 | 17 |  | 1 |
| CCDC57 |  |  |  |  |  | 44 |  |  |  |  |  |  |  |  |  | 2 |  |  |  | 6 |
| HLA-DRB5 |  | 75 |  |  |  |  |  |  |  |  | 6 |  | 6 |  | 4 |  |  |  |  |  |
| MYH3 |  | 83 |  | 100 | 76 |  |  |  |  |  | 31 | 16 | 10 | 20 | 17 |  |  |  |  |  |
| CMYA5 |  |  |  | 72 |  |  |  |  |  |  | 29 | 20 | 55 | 11 | 10 |  |  |  |  |  |
| SUMF1 |  |  |  |  |  |  |  |  |  |  |  |  | 68 |  |  |  | 34 | 6 |  |  |
| ALPK2 | 14 | 29 | 26 | 57 | 19 | 63 |  | 98 |  | 93 | 35 | 10 | 61 | 14 | 27 |  |  |  |  |  |
| ZNF280A | 7 | 8 | 14 | 8 | 10 | 35 | 51 | 45 | 44 | 49 | 67 | 47 | 17 | 35 | 61 |  |  |  |  |  |
| OR51F1 | 5 | 17 | 11 |  |  | 19 | 29 | 26 |  |  | 45 | 17 | 30 | 33 |  |  |  |  |  |  |
| PSMD13 | 33 | 22 | 7 | 19 | 13 | 20 | 10 | 11 | 29 | 14 | 36 | 30 | 19 |  | 34 |  |  |  |  |  |
| FLG |  | 42 |  | 35 | 30 | 91 |  |  |  |  | 2 |  | 35 | 40 |  |  |  |  |  |  |
| HLA-A | 32 | 15 | 17 | 18 | 25 | 4 |  | 3 | 4 | 6 | 5 | 3 | 3 | 1 | 1 |  |  |  |  |  |
| SP110 | 13 | 62 | 13 | 11 | 9 | 30 | 34 | 29 | 32 |  |  |  | 53 |  |  |  |  |  |  |  |
| OR2T4 | 15 | 10 | 57 | 5 | 4 | 21 | 42 | 28 | 18 | 26 |  | 51 |  | 39 |  |  |  |  |  |  |
| OR5P2 | 9 | 4 | 9 |  |  | 16 | 7 | 20 | 23 |  | 28 | 28 | 14 | 31 | 36 |  |  |  |  |  |
| OR51B5 | 1 | 3 | 3 | 4 | 5 | 3 | 8 | 7 | 10 | 9 | 19 | 26 | 16 | 13 | 12 |  |  |  |  |  |
| UGT1A5 | 64 | 38 |  | 33 | 8 | 12 | 15 | 35 | 28 | 21 | 30 | 21 |  | 37 | 28 |  |  |  |  |  |
| AHNAK2 | 60 | 97 | 54 | 7 | 67 | 47 |  |  | 17 | 34 | 1 | 2 | 8 | 3 | 2 |  |  |  |  |  |
| UGT1A6 | 64 | 38 |  | 33 | 8 | 12 | 15 | 35 | 28 | 21 | 30 | 21 |  | 37 | 28 |  |  |  |  |  |
| TAS2R43 | 36 | 11 | 4 |  | 3 | 8 | 19 | 6 | 41 | 5 | 8 | 9 | 18 | 9 | 8 |  |  |  |  |  |
| HLA-DPA1 | 2 | 5 | 5 | 2 | 1 |  |  | 5 |  | 2 | 12 |  | 1 |  | 9 |  |  |  |  |  |
| MMP26 | 5 | 17 | 11 |  |  | 19 | 29 | 26 |  |  | 45 | 17 | 30 | 33 |  |  |  |  |  |  |
| OR6K6 | 3 |  |  |  |  | 7 | 13 |  |  |  | 24 | 12 |  |  |  |  |  |  |  |  |
| OR51B6 | 1 |  |  |  |  | 3 | 8 | 7 | 10 | 9 | 19 | 26 | 16 | 13 | 12 |  |  |  |  |  |
| MYO3A |  |  | 34 |  | 51 | 34 | 27 | 14 | 16 | 20 |  |  |  |  |  | 84 |  | 1 | 5 | 28 |
| ESYT2 |  |  |  | 55 |  |  |  |  | 9 |  |  |  |  |  |  | 86 |  |  | 3 |  |
| RGMA | 35 | 13 | 23 |  | 54 |  | 80 | 89 |  | 98 |  |  |  |  |  | 68 | 18 | 9 |  |  |
| FCER2 |  |  | 48 |  | 74 | 6 |  | 2 |  | 4 |  |  |  |  |  | 3 |  |  |  |  |
| GPC6 | 6 | 7 | 6 | 12 | 7 | 32 | 47 | 39 | 42 | 43 |  |  |  |  |  | 26 | 17 | 4 | 56 |  |
| DMBT1 |  |  | 52 |  | 63 | 15 | 9 | 9 | 8 | 8 |  |  |  |  |  |  |  | 82 | 18 | 23 |
| HLA-DPB1 |  |  |  |  |  | 2 | 2 |  | 2 |  |  | 29 |  |  |  |  |  |  | 2 |  |
| SPATA3 |  |  |  |  |  |  | 86 | 97 | 71 | 40 |  | 80 |  |  |  | 45 |  |  | 35 | 10 |
| SPINK5 |  |  |  |  |  |  | 62 | 73 |  | 50 |  |  | 78 | 90 | 99 |  | 77 | 86 | 48 | 5 |
| HRNR | 77 |  |  |  |  |  |  |  |  |  |  | 82 |  |  | 55 |  | 10 |  |  |  |
| PRH1-PRR4 | 36 | 11 | 4 | 22 | 3 | 8 | 19 | 6 | 41 | 5 | 8 | 9 | 18 | 9 | 8 |  |  | 69 |  |  |
| UGT1A7 | 64 | 38 | 85 | 33 | 8 | 12 | 15 | 35 | 28 | 21 | 30 | 21 |  | 37 | 28 |  |  | 53 |  |  |
| ZNF568 | 10 | 9 | 10 | 15 | 11 | 53 | 50 | 33 | 58 | 45 | 65 | 45 | 21 | 36 | 47 |  | 84 | 19 |  | 79 |
| GBP4 | 30 | 21 | 12 | 6 | 17 | 25 | 36 | 34 | 35 | 37 | 55 | 37 | 13 | 29 | 44 |  |  | 64 |  |  |
| M1 | 12 | 12 | 16 | 23 | 18 | 40 | 46 | 46 | 56 | 55 | 56 | 15 | 9 | 70 | 26 | 66 | 70 | 34 | 43 | 27 |
| UGT1A8 | 64 | 38 | 85 | 33 | 8 | 12 | 15 | 35 | 28 | 21 | 30 | 21 |  | 37 | 28 |  |  | 53 |  |  |
| PRH1 | 36 | 11 | 4 | 22 | 3 | 8 | 19 | 6 | 41 | 5 | 8 | 9 | 18 | 9 | 8 |  |  | 69 |  |  |
| COL5A1 | 22 | 34 | 30 | 20 | 22 | 89 |  | 19 | 69 | 16 |  | 74 | 37 | 83 | 69 | 42 | 81 | 10 | 13 | 30 |
| HLA-C |  | 2 | 2 | 3 | 12 | 1 | 1 | 1 | 1 | 1 | 7 | 6 |  |  |  |  | 16 |  | 20 |  |
| ACSF3 | 21 | 28 | 29 | 16 | 14 | 27 | 22 | 22 | 24 | 22 |  |  |  | 91 |  | 18 |  |  |  | 3 |
| FNDC1 | 41 |  |  | 10 |  | 57 |  | 70 | 51 |  |  | 44 | 20 | 59 | 56 |  |  |  | 52 |  |
| UGT1A10 | 64 | 38 | 85 | 33 | 8 | 12 | 15 | 35 | 28 | 21 | 30 | 21 |  | 37 | 28 |  |  | 53 |  |  |
| PRH1-TAS2R14 | 36 | 11 | 4 | 22 | 3 | 8 | 19 | 6 | 41 | 5 | 8 | 9 | 18 | 9 | 8 |  |  | 69 |  |  |
| UGT1A9 | 64 | 38 | 85 | 33 | 8 | 12 | 15 | 35 | 28 | 21 | 30 | 21 |  | 37 | 28 |  |  | 53 |  |  |
| MUC16 | 68 | 32 | 28 | 13 | 68 | 45 | 77 | 55 | 52 |  | 4 | 4 | 2 | 5 | 5 |  |  | 52 | 23 |  |
| PSCA | 43 | 20 | 24 | 56 | 26 | 18 | 23 | 16 |  | 15 |  | 69 | 40 | 81 | 88 | 4 | 6 | 5 |  |  |
| SIRPA | 4 | 1 | 1 | 1 | 2 | 5 | 3 | 4 | 3 | 3 | 16 |  | 29 | 8 | 14 |  |  |  | 34 |  |
| CNR2 | 11 | 6 | 8 | 9 | 6 | 35 | 31 | 17 | 19 | 11 | 57 | 38 | 23 | 30 | 41 |  | 2 | 7 | 33 |  |
| RAB36 |  | 86 | 92 | 97 | 78 | 11 | 6 | 8 | 11 | 13 |  | 76 | 47 | 78 | 93 | 29 | 1 | 2 | 7 | 2 |

**Supplemental Table 6** **Comparison of statistics top 10 peaks of selection signal from genome wide scan of clinical samples.** Above are genes where at least one of the four methods LD-ABF, D_ng_, B2, or Tajima’s D has a top 10 selection signal identified in it where the number in each cell corresponds to the rank of that peak using a peak finding of 1Mb windows.

|  | LD-ABF | | | | | Dng | | | | | B2 | | | | | TD | | | | |
| --- | --- | --- | --- | --- | --- | --- | --- | --- | --- | --- | --- | --- | --- | --- | --- | --- | --- | --- | --- | --- |
| Genes | AF | AM | EU | EA | SA | AF | AM | EU | EA | SA | AF | AM | EU | EA | SA | AF | AM | EU | EA | SA |
| *AADACL3* |  |  |  | 75 |  |  |  |  |  |  |  |  |  |  |  |  |  |  |  |  |
| *ARHGEF19* |  |  |  |  | 100 |  |  |  |  |  |  |  |  |  |  |  |  |  |  |  |
| *CFAP61* |  |  | 67 |  |  |  |  |  |  |  |  |  |  |  |  |  |  |  |  |  |
| *CRNKL1* | 85 |  | 67 |  | 57 |  |  |  |  |  |  |  |  |  |  |  |  |  |  |  |
| *KCNQ2* |  |  |  | 89 |  |  |  |  |  |  |  |  |  |  |  |  |  |  |  |  |
| *MRGPRX4* |  |  |  | 67 |  |  |  |  |  |  |  |  |  |  |  |  |  |  |  |  |
| *OR52Z1* | 96 |  |  |  | 91 |  |  |  |  |  |  |  |  |  |  |  |  |  |  |  |
| *PAX2* | 74 |  |  |  |  |  |  |  |  |  |  |  |  |  |  |  |  |  |  |  |
| *PCARE* |  |  | 84 | 58 | 69 |  |  |  |  |  |  |  |  |  |  |  |  |  |  |  |
| *SNHG14^a^* | 70 | 61 | 61 | 46 | 61 |  |  |  |  |  |  |  |  |  |  |  |  |  |  |  |
| *SNRPN^a^* | 70 | 61 | 61 | 46 | 61 |  |  |  |  |  |  |  |  |  |  |  |  |  |  |  |
| *CCDC50** | 92 | 89 |  |  |  |  |  |  |  |  |  |  |  |  |  |  |  |  |  |  |
| *FAM214A** |  |  |  | 78 |  |  |  |  |  |  |  |  |  |  |  |  |  |  |  |  |
| *LRRC32** |  | 76 | 88 |  |  |  |  |  |  |  |  |  |  |  |  |  |  |  |  |  |
| *OR13G1** |  |  |  |  | 85 |  |  |  |  |  |  |  |  |  |  |  |  |  |  |  |
| *CYP4F2* |  |  |  |  | 86 |  |  |  |  |  |  |  |  |  |  |  |  |  |  |  |
| *KRTAP7-1* |  |  |  |  |  |  |  |  |  |  |  |  |  |  |  |  |  | 78 |  | 87 |
| *HLA-H* |  |  | 83 |  |  | 8 | 8 | 8 | 9 | 32 |  |  |  |  |  |  |  |  |  |  |
| *KRTAP10-9* | 61 | 64 | 74 | 60 | 49 |  |  |  |  | 89 |  |  |  |  |  |  |  |  |  |  |
| *TRMT9B* | 75 | 90 | 75 | 59 | 48 |  |  |  | 98 |  |  |  |  |  |  |  |  |  |  |  |
| *CMYA5* |  |  |  | 93 |  |  |  |  |  |  | 37 | 25 | 77 | 17 | 13 |  |  |  |  |  |
| *KLHDC7A* |  |  |  |  | 93 |  |  |  |  |  |  | 65 |  |  |  |  |  |  |  |  |
| *MYH3* |  |  |  |  | 99 |  |  |  |  |  | 39 | 21 | 14 | 31 | 24 |  |  |  |  |  |
| *OR10G9* |  |  |  | 92 |  |  |  |  |  |  | 32 | 64 |  |  | 51 |  |  |  |  |  |
| *ZNF45* |  | 65 | 51 |  | 47 |  |  |  |  |  |  |  | 59 |  |  |  |  |  |  |  |
| *ZNF778* | 65 |  |  |  |  |  |  |  |  |  |  |  | 54 |  | 69 |  |  |  |  |  |
| *ADGRF2* |  |  |  | 86 |  |  |  |  |  |  |  |  |  |  |  |  | 39 | 51 |  |  |
| *HCG20* | 34 | 40 | 43 | 32 | 53 |  |  |  |  |  |  |  |  |  |  | 84 | 95 | 28 | 78 |  |
| *ONECUT2* |  | 74 |  | 51 |  |  |  |  |  |  |  |  |  |  |  |  | 28 |  | 87 |  |
| *PLEKHG4B* |  |  |  |  | 81 |  |  |  |  |  |  |  |  |  |  |  | 88 |  |  |  |
| *QRICH2* | 47 | 69 | 72 | 29 | 72 |  |  |  |  |  |  |  |  |  |  |  |  |  | 71 |  |
| *SELENOO* |  |  |  | 83 | 94 |  |  |  |  |  |  |  |  |  |  |  |  |  |  | 96 |
| *OR1S1* | 28 | 19 | 28 | 28 | 25 | 81 | 100 |  | 78 |  |  |  | 98 |  |  |  |  |  |  |  |
| *OR2T4* | 18 | 15 | 76 | 6 | 4 | 27 | 54 | 34 | 22 | 30 | 44 | 66 |  | 57 | 89 |  |  |  |  |  |
| *PGAP6* |  | 54 | 50 | 94 | 37 | 93 | 55 | 54 |  | 28 |  |  | 100 |  |  |  |  |  |  |  |
| *PLEC* |  |  | 93 |  |  |  |  | 38 |  |  |  | 18 | 41 | 92 |  |  |  |  |  |  |
| *COL5A1* | 29 | 45 | 44 | 24 | 29 |  |  | 24 | 84 | 18 |  | 100 | 49 |  | 93 | 43 | 90 | 10 | 15 | 31 |
| *HCG17* |  | 72 |  | 63 | 62 |  | 27 |  | 25 | 36 |  | 83 | 67 | 44 | 74 |  |  |  | 3 | 30 |

**Supplemental Table 7 New signals of balancing selection that fall within genes not previously noted based on genome wide scan of clinical samples.** The set of new selection signals not previously found in these genes before with their corresponding rank for each statistic using 100Kb peak finding listed for the given statistic and corresponding population. The *denotes a gene that corresponds to new but, found using LD-ABF with the other peak finding window size of 1Mb. Genes marked with the subscript “a” were also recognized as part of the top 100 peaks using methods other than LD-ABF, where peak detection was conducted with a different window size of 1Mb.

| Gene | HGNC ID (gene) | Group |
| --- | --- | --- |
| ALPK2 | HGNC:20565 | I-set domain containing |
| CD200R1 | HGNC:24235 | C2-set domain containing |
| HLA-A | HGNC:4931 | C1-set domain containing |
| HLA-B | HGNC:4932 | C1-set domain containing |
| HLA-C | HGNC:4933 | C1-set domain containing |
| HLA-DPA1 | HGNC:4938 | C1-set domain containing |
| HLA-DQA1 | HGNC:4942 | C1-set domain containing |
| HLA-DQB1 | HGNC:4944 | C1-set domain containing |
| HLA-DRB5 | HGNC:4953 | C1-set domain containing |
| HLA-G | HGNC:4964 | C1-set domain containing |
| IL1RL1 | HGNC:5998 | I-set domain containing |
| LILRA1 | HGNC:6602 | Activating leukocyte immunoglobulin like receptors |
| LILRB2 | HGNC:6606 | Inhibitory leukocyte immunoglobulin like receptors |
| MICA | HGNC:7090 | C1-set domain containing |
| SIGLEC16 | HGNC:24851 | C2-set domain containing, I-set domain containing, Sialic acid binding Ig like lectins, V-set domain containing |
| SIRPA | HGNC:9662 | C1-set domain containing, V-set domain containing |

**Supplemental Table 8 HGNC defined immunoglobulin superfamily** **genes that were found in the top 100 balancing selection peaks across the CHOP trio samples**. This includes both the set of genes restricting to a 1Mb window and 100Kb window for defining peaks.

| Pop | Chrom | ID | LD-ABF | | Gene | | Disease/Trat | Sequence Context |
| --- | --- | --- | --- | --- | --- | --- | --- | --- |
| EAS | 20 | rs17855611 | *0.26* | SIRPA | | Blood protein levels | | missense variant |
|  | 6 | rs1126506 | *0.14* | HLA | | Anti-rubella virus IgG levels | | splice region variant |
|  | 6 | rs9260151 | *0.14* | MHC, HLA-A | | C-peptide levels in type I diabetes | | noncoding transcript exon variant |
|  | 6 | rs34794906 | *0.13* | HLA-C | | Reticulocyte count | | synonymous variant |
|  | 6 | rs2858331 | *0.13* | HLA-DQA2 | | IgE levels | | regulatory region variant |
|  | 6 | rs2516703 | *0.13* | HCG17 | | Itch intensity from mosquito bite | | intron variant |
|  | 9 | rs8176743 | *0.13* | ABO | | End-stage coagulation, Intraocular pressure, Mean corpuscular volume, Mean corpuscular hemoglobin, Blood protein levels | | missense variant |
|  | 9 | rs8176749 | *0.13* | ABO | | Tumor biomarkers, Urinary metabolites (H-NMR features), Mean corpuscular volume, Venous thromboembolism, Mean corpuscular hemoglobin concentration | | synonymous variant |
|  | 9 | rs8176749 | *0.13* | ABO, Y_RNA, LCN1P2 | | Blood protein levels | | synonymous variant |
|  | 9 | rs8176746 | *0.13* | ABO | | Mean corpuscular hemoglobin concentration, Mean corpuscular volume, Blood protein levels | | missense variant |
|  | 9 | rs8176746 | *0.13* | NR | | Hemoglobin, Red cell distribution width | | missense variant |
|  | 1 | rs4525 | *0.12* | F5 | | Blood protein levels | | missense variant |
|  | 1 | rs4524 | *0.12* | F5 | | Venous thromboembolism | | missense variant |
|  | 9 | rs8176747 | *0.12* | ABO | | Platelet count, Reticulocyte count, Blood protein levels, Intraocular pressure | | missense variant |
|  | 9 | rs8176741 | *0.12* | ABO | | Elevated serum carcinoembryonic antigen levels, Intraocular pressure | | synonymous variant |
|  | 9 | rs8176741 | *0.12* | RALGDS | | Blood protein levels in cardiovascular risk | | synonymous variant |
|  | 22 | rs5771225 | *0.12* | SELO | | Late-onset Alzheimer's disease | | missense variant |
|  | 11 | rs5006884 | *0.11* | OR51B6 | | Fetal hemoglobin levels | | missense variant |
|  | 6 | rs1050451 | *0.11* | HLA-B, HLA-C | | IgG galactosylation phenotypes (multivariate analysis) | | missense variant |
| SAS | 6 | rs1126506 | *0.17* | HLA | | Anti-rubella virus IgG levels | | splice region variant |
|  | 6 | rs9277354 | *0.17* | HLA-DPB1 | | Antineutrophil cytoplasmic antibody-associated vasculitis | | frameshift variant |
|  | 6 | rs9277356 | *0.17* | HLA-DPB1 | | Response to hepatitis B vaccine | | missense variant |
|  | 11 | rs5006884 | *0.16* | OR51B6 | | Fetal hemoglobin levels | | missense variant |
|  | 20 | rs17855611 | *0.10* | SIRPA | | Blood protein levels | | missense variant |
|  | 10 | rs2249694 | *0.10* | CYP2E1 | | Obesity-related traits | | intron variant |
|  | 6 | rs9260151 | *0.10* | MHC, HLA-A | | C-peptide levels in type I diabetes | | noncoding transcript exon variant |
|  | 6 | rs2516703 | *0.09* | HCG17 | | Itch intensity from mosquito bite | | intron variant |
|  | 6 | rs2894204 | *0.09* | HLA-C | | Waist-hip ratio | | intron variant |
|  | 9 | rs8176749 | *0.08* | ABO | | Tumor biomarkers, Urinary metabolites (H-NMR features), Mean corpuscular volume, Mean corpuscular hemoglobin concentration | | synonymous variant |
|  | 9 | rs8176749 | *0.08* | ABO, Y_RNA, LCN1P2 | | Blood protein levels | | synonymous variant |
|  | 9 | rs8176746 | *0.08* | ABO | | Mean corpuscular hemoglobin concentration, Mean corpuscular volume, Blood protein levels | | missense variant |
|  | 9 | rs8176746 | *0.08* | NR | | Hemoglobin, Red cell distribution width | | missense variant |
|  | 9 | rs8176743 | *0.08* | ABO | | End-stage coagulation, Intraocular pressure, Mean corpuscular volume, Mean corpuscular hemoglobin, Blood protein levels | | missense variant |
|  | 1 | rs4525 | *0.08* | F5 | | Blood protein levels | | missense variant |
|  | 6 | rs1042151 | *0.08* | HLA-DPB1 | | Aspirin exacerbated respiratory disease in asthmatics, Severe aplastic anemia | | missense variant |
|  | 1 | rs2992753 | *0.08* | KLHDC7A | | LDL cholesterol | | missense variant |
|  | 22 | rs5771225 | *0.08* | SELO | | Late-onset Alzheimer's disease | | missense variant |

**Supplemental Table 9 top Balancing selection GWAS significant SNPs with strong signals of selection in the top 99.9% looking at the CHOP trios for the EAS and SAS populations (continuation of Table 3).**

| *Population* | *Gene* | *POS* | *ID* | *LD-ABF* | *DISEASE/TRAIT* | *CONTEXT* | *PUBMEDID* |
| --- | --- | --- | --- | --- | --- | --- | --- |
| *European American* | DQA1 | 32606756 | rs9272535 | 198.97 | Red blood cell count | Missense | 27863252 |
| *European American* | DQA1 | 32606756 | rs9272535 | 198.97 | Chronic lymphocytic leukemia | Missense | 21131588 |
| *European American* | DQB1 | 32632659 | rs9274390 | 160.36 | Autism spectrum disorder or schizophrenia | Missense | 28540026 |
| *European American* | DRB1 | 32556601 | rs28724212 | 159.45 | Autism spectrum disorder or schizophrenia | Intronic | 28540026 |
| *European American* | DQB1 | 32628538 | rs201043192 | 133.09 | Lung function (low FEV1 vs high FEV1) | Intronic | 26423011 |
| *European American* | DRB1 | 32550322 | rs9269853 | 128.65 | Alzheimer's disease (late onset) | Intronic | 30617256 |
| *European American* | DRB1 | 32552095 | rs17885382 | 124.85 | Asparaginase hypersensitivity in acute lymphoblastic leukemia | Missense | 25987655 |
| *European American* | DQA1 | 32606878 | rs9272544 | 123.38 | Granulocyte percentage of myeloid white cells | Missense | 27863252 |
| *European American* | DRB1 | 32554129 | rs9270074 | 114.73 | Autism spectrum disorder or schizophrenia | Intronic | 28540026 |
| *European American* | DQA1 | 32608858 | rs4455710 | 110.42 | Squamous cell carcinoma | Intronic | 26829030 |
| *European American* | DQB1 | 32632887 | rs201184533 | 110.13 | Asthma | Intronic | 30929738 |
| *African American* | DQA1 | 32606756 | rs9272535 | 32.30 | Red blood cell count | Missense | 27863252 |
| *African American* | DQA1 | 32606756 | rs9272535 | 32.30 | Chronic lymphocytic leukemia | Missense | 21131588 |
| *African American* | DQB1 | 32632659 | rs9274390 | 25.27 | Autism spectrum disorder or schizophrenia | Missense | 28540026 |
| *African American* | DQB1 | 32628538 | rs201043192 | 19.30 | Lung function (low FEV1 vs high FEV1) | Intronic | 26423011 |
| *African American* | DQB1 | 32632887 | rs201184533 | 19.04 | Asthma | Intronic | 30929738 |
| *African American* | DQB1 | 32632832 | rs9274407 | 16.98 | Drug-induced liver injury (amoxicillin-clavulanate) | Missense | 21570397 |
| *African American* | DRB1 | 32550322 | rs9269853 | 16.96 | Alzheimer's disease (late onset) | Intronic | 30617256 |
| *African American* | DQA1 | 32608858 | rs4455710 | 16.74 | Squamous cell carcinoma | Intronic | 26829030 |
| *African American* | DRB1 | 32554129 | rs9270074 | 16.72 | Autism spectrum disorder or schizophrenia | Intronic | 28540026 |
| *African American* | DRB1 | 32556601 | rs28724212 | 14.76 | Autism spectrum disorder or schizophrenia | Intronic | 28540026 |
| *African American* | DQA1 | 32606878 | rs9272544 | 14.02 | Granulocyte percentage of myeloid white cells | Missense | 27863252 |

**Supplemental Table 10** **top balancing selection signals in 17^th^ IHIW samples at GWAS significant SNPs.** Signals of balancing selection found in HLA genes within European American and African American populations from 17^th^ IHIW samples that occur at known GWAS significant SNPs.

|  | Top 99.9% Percentile | Sample Size (N) | Percentile/N |
| --- | --- | --- | --- |
| AFR | 0.227 | 54 | 0.0042 |
| EUR | 1.461 | 301 | 0.0049 |
| AMR | 0.281 | 64 | 0.0044 |
| EAS | 0.114 | 28 | 0.0041 |
| SAS | 0.080 | 21 | 0.0038 |

**Supplemental Table 11 The percentile for the LD-ABF test statistics in top 99.9% in clinical populations.** To establish a rule of thumb for a LD-ABF threshold taking the LD-ABF/N > 0.005 is a reasonable threshold based on the clinical trios’ scan where this is more restrictive than looking at the top 99.9%/N across the populations.
